# Supplementary material for: Genetic Correlates of Psychological Responses to the COVID-19 Crisis in Young Adult Twins in Great Britain
Source: Behav Genet. 2021 Feb 24;51(2):110–24. doi: 10.1007/s10519-021-10050-2 (PMC7902241; doi:10.1007/s10519-021-10050-2)
Supplement: Supplementary file 1 — Supplementary file1 (PDF 2192 KB) [file 10519_2021_10050_MOESM1_ESM.pdf]

Supporting information – Genetic correlates of psychological responses to the COVID-19 crisis in young adult twins in Great Britain

Kaili Rimfeld, Margherita Malanchini, Andrea G. Allegrini, Amy E. Packer, Andrew McMillan, Rachel Ogden, Louise Webster, Nicholas G. Shakeshaft, Kerry L. Schofield, Jean-Baptiste Pingault, Argyris Stringaris, Sophie von Stumm, Robert Plomin

Supplementary Tables 6

Supplementary Table 1. Descriptive statistics at T1 for one twin randomly selected from each pair. 6

Supplementary Table 2. Descriptive statistics at T2 for one twin randomly selected from each pair. 8

Supplementary Table 3. Results of F test; variance differences between T1 and T2. a) full sample; b) males and females 11

Supplementary Table 4. Descriptive statistics for T1 to T2 differences for one twin randomly selected from each pair. 14

Supplementary Table 5. Descriptive statistics for T1 for one twin randomly selected from each pair broken down by zygosity. 16

Supplementary Table 6. Descriptive statistics for T2 for one twin randomly selected from each pair broken down by zygosity. 18

Supplementary Table 7. Descriptive statistics for T1 to T2 differences for one twin randomly selected from each pair broken down by zygosity. 20

Supplementary Table 8. Descriptive statistics at T1 for the twin that was not randomly selected from each pair in the main analyses. 22

Supplementary Table 9. Descriptive statistics at T2 for twin two randomly selected from each pair. 25

Supplementary Table 10. Descriptive statistics for T1 to T2 differences for twin two randomly selected from each pair. 27

Supplementary Table 11. Phenotypic correlations between T1 and T2 for the whole sample and split by males and females. 30

Supplementary Table 12. Measures, test-retest reliabilities and references. 31

Supplementary Table 13. Multiple regression analysis of phenotypic moderation of sex on T2 responses. 34

Supplementary Table 14. Multiple regression analysis of phenotypic moderation of SES on T2 responses. 35

Supplementary Table 15. Multiple regression analysis of phenotypic moderation of job loss/financial difficulties on T2 responses. 37

Supplementary Table 16. Multiple regression analysis of phenotypic moderation of garden access on T2 responses. 38

Supplementary Table 17. Multiple regression analysis of phenotypic moderation of living conditions (number of rooms: people in home during the lockdown) on T2 responses. 40

Supplementary Table 18. Multiple regression analysis of phenotypic moderation of having been tested or suspected of having COVID-19 on T2 responses. 41

Supplementary Table 19. Multiple regression analysis of phenotypic moderation of COVID-19 symptoms on T2 responses. 43

Supplementary Table 20. Multiple regression analysis of phenotypic moderation of COVID-19 symptoms (2 or more *vs.* none) on T2 responses. 44

Supplementary Table 21. Multiple regression analysis of phenotypic moderation of living conditions (twins together *vs.* apart) on T2 responses. 46

Supplementary Table 22. Multiple regression analysis of phenotypic moderation of living conditions (living alone) on T2 responses. 47

Supplementary Table 23. Multiple regression analysis of phenotypic moderation of COVID-19 impact on family health on T2 responses. 49

Supplementary Table 24. Multiple regression analysis of phenotypic moderation of COVID-19 impact on family financial situation on T2 responses. 50

Supplementary Table 25. Multiple regression analysis of phenotypic moderation of COVID-19 impact on family health and financial situation on T2 responses. 52

Supplementary Table 26. Multiple regression analysis of phenotypic moderation of family death due to COVID-19 on T2 responses. 53

Supplementary Table 27. Multiple regression analysis of phenotypic moderation of worries of being infected on T2 responses. 55

Supplementary Table 28. Multiple regression analysis of phenotypic moderation of worries of family or friends being infected on T2 responses. 56

Supplementary Table 29. Multiple regression analysis of phenotypic moderation of worries of physical health being influenced by COVID-19 on T2 responses. 58

Supplementary Table 30. Multiple regression analysis of phenotypic moderation of worries of mental/emotional health being influenced by COVID-19 on T2 responses. 59

Supplementary Table 31. Multiple regression analysis of phenotypic moderation of change in sleep habits on T2 responses. 61

Supplementary Table 32. Multiple regression analysis of phenotypic moderation of having children (continuous variable) on T2 responses. 62

Supplementary Table 33. Multiple regression analysis of phenotypic moderation of having children (yes/no) on T2 responses. 64

Supplementary Table 34. Twin intraclass correlations and Falconer ACE estimates for T1............ 65

Supplementary Table 35. Twin intraclass correlations and Falconer ACE estimates for T2............ 67

Supplementary Table 36. Twin intraclass correlations and Falconer ACE estimates for T2 change.68

Supplementary Table 37. Whole sample model fitting results for univariate analyses of additive genetic (A), shared environmental (C), and non-shared environmental (E) components of variance for variables (95% confidence intervals in parentheses). 69

Supplementary Table 38. Bivariate Cholesky decomposition estimating the aetiology of the association between T1 (A1, C1 and E1) and T2 (A2, C2 and E2), for variable in the relationships theme (95% confidence intervals in parentheses). 71

Supplementary Table 39. Bivariate Cholesky decomposition estimating the aetiology of the association between T1 (A1, C1 and E1) and T2 (A2, C2 and E2), for variables in the thoughts and attitudes theme (95% confidence intervals in parentheses). 71

Supplementary Table 40. Bivariate Cholesky decomposition estimating the aetiology of the association between T1 (A1, C1 and E1) and T2 (A2, C2 and E2), for variables in the substance use theme (95% confidence intervals in parentheses). 73

Supplementary Table 41. Bivariate Cholesky decomposition estimating the aetiology of the association between T1 (A1, C1 and E1) and T2 (A2, C2 and E2), for variables in the wellbeing theme (95% confidence intervals in parentheses). 73

Supplementary Table 42. Bivariate Cholesky decomposition estimating the aetiology of the association between T1 (A1, C1 and E1) and T2 (A2, C2 and E2), for variables in the behaviours theme (95% confidence intervals in parentheses). 75

Supplementary Table 43. Bivariate model fit indices 76

Supplementary Table 44. For twins living together: T1 twin intraclass correlations and model fitting results for univariate analyses of additive genetic (A), shared environmental (C), and non-shared environmental (E) components of variance (95% confidence intervals in parentheses). 77

Supplementary Table 45. For twins living together: T2 twin intraclass correlations and model fitting results for univariate analyses of additive genetic (A), shared environmental (C), and non-shared environmental (E) components of variance (95% confidence intervals in parentheses). 79

Supplementary Table 46. For twins living together: T2 change twin intraclass correlations and model fitting results for univariate analyses of additive genetic (A), shared environmental (C), and non-shared environmental (E) components of variance (95% confidence intervals in parentheses). 80

Supplementary Table 47. For twins living apart: T1 twin intraclass correlations and model fitting results for univariate analyses of additive genetic (A), shared environmental (C), and non-shared environmental (E) components of variance (95% confidence intervals in parentheses). 82

Supplementary Table 48. For twins living apart: T2 twin intraclass correlations and model fitting results for univariate analyses of additive genetic (A), shared environmental (C), and non-shared environmental (E) components of variance (95% confidence intervals in parentheses). 83

Supplementary Table 49. For twins living apart: T2 change twin intraclass correlations and model fitting results for univariate analyses of additive genetic (A), shared environmental (C), and non-shared environmental (E) components of variance (95% confidence intervals in parentheses). 85

Supplementary Table 50. Male model fitting results for univariate analyses of additive genetic (A), shared environmental (C), and non-shared environmental (E) components of variance (95% confidence intervals in parentheses). 87

Supplementary Table 51. Female model fitting results for univariate analyses of additive genetic (A), shared environmental (C), and non-shared environmental (E) components of variance (95% confidence intervals in parentheses). 88

Supplementary Table 52. Model fitting results for univariate analyses of additive genetic (A), shared environmental (C), and non-shared environmental (E) components of variance (95% confidence intervals in parentheses) for variables corrected for variation in SES. 90

Supplementary Table 53. Model fitting results for univariate analyses of additive genetic (A), shared environmental (C), and non-shared environmental (E) components of variance (95% confidence intervals in parentheses) for variables corrected for variation in job/financial difficulties. 92

Supplementary Table 54. Model fitting results for univariate analyses of additive genetic (A), shared environmental (C), and non-shared environmental (E) components of variance (95% confidence intervals in parentheses) for variables corrected for variation in garden access during the lockdown. 93

Supplementary Table 55. Model fitting results for univariate analyses of additive genetic (A), shared environmental (C), and non-shared environmental (E) components of variance (95% confidence intervals in parentheses) for variables corrected for variation in living conditions (number of rooms: people in home during the lockdown). 95

Supplementary Table 56. Model fitting results for univariate analyses of additive genetic (A), shared environmental (C), and non-shared environmental (E) components of variance (95% confidence intervals in parentheses) for variables corrected for variation in having been tested or suspected of having COVID-19. 96

Supplementary Table 57. Model fitting results for univariate analyses of additive genetic (A), shared environmental (C), and non-shared environmental (E) components of variance for variables (95% confidence intervals in parentheses) corrected for variation in number of COVID-19 symptoms. 97

Supplementary Table 58. Model fitting results for univariate analyses of additive genetic (A), shared environmental (C), and non-shared environmental (E) components of variance for variables (95% confidence intervals in parentheses) for variables corrected for variation in number of COVID-19 symptoms (two or more) vs none. 99

Supplementary Table 59. Model fitting results for univariate analyses of additive genetic (A), shared environmental (C), and non-shared environmental (E) components of variance for variables (95% confidence intervals in parentheses) for variables corrected for impact of COVID-19 on family health. 100

Supplementary Table 60. Model fitting results for univariate analyses of additive genetic (A), shared environmental (C), and non-shared environmental (E) components of variance for variables (95% confidence intervals in parentheses) for variables corrected for financial impact of COVID-19 on family. 102

Supplementary Table 61. Model fitting results for univariate analyses of additive genetic (A), shared environmental (C), and non-shared environmental (E) components of variance for variables (95% confidence intervals in parentheses) for variables corrected for health and financial impact of COVID-19 on family. 103

Supplementary Table 62. Model fitting results for univariate analyses of additive genetic (A), shared environmental (C), and non-shared environmental (E) components of variance for variables (95% confidence intervals in parentheses) for variables corrected for worries of being infected. 104

Supplementary Table 63. Model fitting results for univariate analyses of additive genetic (A), shared environmental (C), and non-shared environmental (E) components of variance for variables

(95% confidence intervals in parentheses) for variables corrected for worries of friends or family being infected. 106

Supplementary Table 64. Model fitting results for univariate analyses of additive genetic (A), shared environmental (C), and non-shared environmental (E) components of variance for variables (95% confidence intervals in parentheses) for variables corrected for worries of mental/emotional health being influenced by COVID-19. 107

Supplementary Table 65. Model fitting results for univariate analyses of additive genetic (A), shared environmental (C), and non-shared environmental (E) components of variance for variables (95% confidence intervals in parentheses) for variables corrected for worries of physical health being influenced by COVID-19. 109

Supplementary Table 66. Model fitting results for univariate analyses of additive genetic (A), shared environmental (C), and non-shared environmental (E) components of variance for variables (95% confidence intervals in parentheses) for variables corrected for change in sleep habits. 110

Supplementary Figures 112

Supplementary Figure 1. Variance in T2 change scores. 112

Supplementary Figure 2. Shared environmental (rC) and nonshared environmental correlations (rE). 113

Supplementary Figure 3. The Bivariate Cholesky decomposition. 114

Supplementary Tables

Supplementary Table 1. Descriptive statistics at T1 for one twin randomly selected from each pair.

Construct	All					Males					Females					ANOVA[a]		
	N	Min	Max	*M*	*SD*	*n*	Min	Max	*M*	*SD*	*n*	Min	Max	*M*	*SD*	Sex	*p*	R^2
Love and relationships	2548	1.00	5.00	3.57	1.21	846	1.00	5.00	3.39	1.19	1702	1.00	5.00	3.66	1.21	28.09	<.001	.01
Verbal peer victimisation	2349	0.00	8.00	2.01	2.52	765	0.00	8.00	2.43	2.73	1584	0.00	8.00	1.80	2.39	29.43	<.001	.01
Cyber peer victimisation	2349	0.00	8.00	0.56	1.41	765	0.00	8.00	0.52	1.40	1584	0.00	8.00	0.58	1.42	0.94	.333	.00
Physical peer victimisation	2349	0.00	8.00	0.19	0.79	765	0.00	8.00	0.32	0.99	1584	0.00	8.00	0.12	0.66	23.75	<.001	.01
Achievement motivation	2553	0.00	16.00	10.06	2.92	849	0.00	16.00	9.88	3.16	1704	0.00	16.00	10.15	2.78	4.46	.035	.00
Importance of Relationships	2553	0.00	20.00	15.84	3.59	849	0.00	20.00	14.59	3.96	1704	0.00	20.00	16.47	3.22	144.17	<.001	.06
Purpose in Life	2552	1.00	5.00	3.45	0.81	849	1.00	5.00	3.38	0.84	1703	1.00	5.00	3.49	0.79	9.58	.002	.00
Healthcare	2549	1.00	5.00	4.58	0.70	847	1.00	5.00	4.48	0.79	1702	1.00	5.00	4.63	0.64	21.34	<.001	.01
Community satisfaction	2539	1.00	5.00	3.60	0.75	842	1.00	5.00	3.59	0.73	1697	1.00	5.00	3.61	0.76	0.36	.552	.00
Attitudes towards money	2490	1.00	5.00	3.05	0.91	836	1.00	5.00	3.14	0.93	1654	1.00	5.00	3.00	0.90	13.90	<.001	.01

Alcohol frequency	2283	0.00	4.00	1.90	0.99	739	0.00	4.00	2.04	1.02	1544	0.00	4.00	1.83	0.97	21.00	<.001	.01
Alcohol quantity	2081	0.00	4.00	2.41	1.26	683	0.00	4.00	2.41	1.27	1398	0.00	4.00	2.41	1.26	0.00	.963	.00
Alcohol (frequency x quantity)	2283	0.00	16.00	4.67	3.68	739	0.00	16.00	5.01	3.86	1544	0.00	16.00	4.51	3.58	8.88	.003	.00
Ever smoked	2389	0.00	1.00	0.54	0.50	771	0.00	1.00	0.53	0.50	1618	0.00	1.00	0.54	0.50	0.59	.444	.00
Smoking frequency	265	1.00	4.00	1.31	0.55	79	1.00	3.00	1.39	0.63	186	1.00	4.00	1.27	0.52	2.17	.143	.01
Ever vaped	2392	0.00	1.00	0.24	0.43	771	0.00	1.00	0.27	0.44	1621	0.00	1.00	0.22	0.42	5.48	.019	.00
Vaping frequency	278	1.00	4.00	1.71	1.17	106	1.00	4.00	1.90	1.27	172	1.00	4.00	1.59	1.09	4.32	.039	.02
Ever used cannabis	2356	0.00	1.00	0.48	0.50	752	0.00	1.00	0.52	0.50	1604	0.00	1.00	0.46	0.50	6.30	.012	.00
Cannabis smoking frequency	398	1.00	5.00	1.57	0.78	164	1.00	5.00	1.71	0.89	234	1.00	5.00	1.48	0.69	7.68	.006	.02
Conduct problems	2522	0.00	8.00	1.51	1.24	841	0.00	8.00	1.53	1.22	1681	0.00	8.00	1.50	1.25	0.37	.541	.00
Emotional problems	2522	0.00	10.00	3.62	2.72	841	0.00	10.00	2.66	2.45	1681	0.00	10.00	4.10	2.72	180.80	<.001	.06
Hyperactivity	2522	0.00	10.00	3.30	2.20	841	0.00	10.00	3.33	2.21	1681	0.00	10.00	3.29	2.19	0.15	.703	.00
Peer problems	2522	0.00	10.00	2.15	1.83	841	0.00	10.00	2.14	1.73	1681	0.00	10.00	2.16	1.88	0.14	.713	.00

Prosocial behaviour	2522	0.00	10.00	7.70	1.89	841	1.00	10.00	7.01	2.01	1681	0.00	10.00	8.04	1.72	160.07	<.001	.07
General anxiety	2382	0.00	40.00	7.56	7.47	765	0.00	40.00	5.96	6.82	1617	0.00	40.00	8.31	7.65	56.86	<.001	.02
Depression	2520	0.00	16.00	4.39	4.12	840	0.00	16.00	3.65	3.68	1680	0.00	16.00	4.77	4.28	46.28	<.001	.02
Self-harm	2371	0.00	4.00	0.21	0.70	762	0.00	4.00	0.16	0.62	1609	0.00	4.00	0.23	0.73	4.85	.028	.00
Physical activity	2528	1.00	5.00	2.80	1.09	837	1.00	5.00	2.90	1.10	1691	1.00	5.00	2.75	1.09	10.43	.001	.00
Media use	2522	0.00	15.00	7.63	2.12	835	0.00	15.00	7.89	2.20	1687	0.00	15.00	7.51	2.07	17.27	<.001	.01
Volunteering	2519	0.00	8.00	2.80	2.10	840	0.00	8.00	2.64	2.03	1679	0.00	8.00	2.89	2.13	8.19	.004	.00

Note: Raw scores are used.

[a] F and p values are reported with Welch's correction (Welch, 1951) as the assumption of homogeneity of variance was violated for several variables.

Supplementary Table 2. Descriptive statistics at T2 for one twin randomly selected from each pair.

**Construct**	**All**					**Males**					**Females**					**ANOVA**[a]		
	N	Min	Max	*M*	*SD*	*n*	Min	Max	*M*	*SD*	*n*	Min	Max	*M*	*SD*	Sex	*p*	R^2
Love and relationships	2042	1.00	5.00	3.68	1.09	633	1.00	5.00	3.57	1.10	1409	1.00	5.00	3.73	1.08	10.61	.001	.01
Verbal peer victimisation	2082	0.00	8.00	0.58	1.48	646	0.00	8.00	0.87	1.85	1436	0.00	8.00	0.45	1.26	22.13	<.001	.01
Cyber peer victimisation	2082	0.00	8.00	0.10	0.55	646	0.00	6.00	0.12	0.62	1436	0.00	8.00	0.09	0.52	1.32	.251	.00

Physical peer victimisation	2082	0.00	8.00	0.04	0.39	646	0.00	6.00	0.05	0.48	1436	0.00	8.00	0.04	0.34	0.65	.421	.00
Achievement motivation	2079	0.00	16.00	8.78	3.36	644	0.00	16.00	8.85	3.46	1435	0.00	16.00	8.75	3.31	0.22	.642	.00
Importance of Relationships	2081	0.00	20.00	15.42	3.71	646	0.00	20.00	14.09	3.98	1435	1.00	20.00	16.03	3.41	109.80	<.001	.06
Purpose in Life	2081	1.00	5.00	3.47	0.81	646	1.00	5.00	3.44	0.83	1435	1.00	5.00	3.48	0.80	0.63	.428	.00
Healthcare	2081	1.00	5.00	4.79	0.59	646	1.00	5.00	4.70	0.71	1435	1.00	5.00	4.84	0.51	16.23	<.001	.01
Community satisfaction	2081	1.00	5.00	3.55	0.70	646	1.40	5.00	3.52	0.67	1435	1.00	5.00	3.56	0.71	1.45	.228	.00
Attitudes towards money	2081	1.00	5.00	3.16	0.90	646	1.00	5.00	3.27	0.94	1435	1.00	5.00	3.12	0.88	9.22	.002	.00
Alcohol frequency	2070	0.00	4.00	2.00	1.27	643	0.00	4.00	2.07	1.33	1427	0.00	4.00	1.96	1.24	2.15	.143	.00
Alcohol quantity	1679	0.00	4.00	1.86	1.26	515	0.00	4.00	2.03	1.27	1164	0.00	4.00	1.79	1.25	9.57	.002	.01
Alcohol (frequency x quantity)	2069	0.00	16.00	4.07	4.26	642	0.00	16.00	4.52	4.53	1427	0.00	16.00	3.86	4.12	7.12	.008	.00
Ever smoked	2071	0.00	1.00	0.47	0.50	643	0.00	1.00	0.49	0.50	1428	0.00	1.00	0.46	0.50	0.47	.495	.00
Smoking frequency	266	1.00	4.00	1.33	0.61	74	1.00	4.00	1.26	0.55	192	1.00	4.00	1.36	0.63	0.78	.379	.00
Ever vaped	2072	0.00	1.00	0.21	0.40	643	0.00	1.00	0.25	0.43	1429	0.00	1.00	0.19	0.39	7.20	.007	.00
Vaping frequency	75	1.00	4.00	2.92	1.23	28	1.00	4.00	3.04	1.11	47	1.00	4.00	2.85	1.30	0.43	.515	.01

Ever used cannabis	2050	0.00	1.00	0.47	0.50	633	0.00	1.00	0.51	0.50	1417	0.00	1.00	0.46	0.50	4.08	.044	.00
Cannabis smoking frequency	148	1.00	5.00	1.64	1.04	56	1.00	5.00	1.73	1.18	92	1.00	5.00	1.58	0.95	0.15	.700	.00
Conduct problems	2069	0.00	7.00	1.51	1.19	643	0.00	7.00	1.50	1.24	1426	0.00	7.00	1.52	1.17	0.93	.336	.00
Emotional problems	2069	0.00	10.00	3.16	2.70	643	0.00	10.00	2.03	2.29	1426	0.00	10.00	3.67	2.72	199.44	<.001	.08
Hyperactivity	2069	0.00	10.00	4.22	2.26	643	0.00	10.00	4.05	2.25	1426	0.00	10.00	4.30	2.26	6.32	.012	.00
Peer problems	2069	0.00	10.00	2.24	1.66	643	0.00	8.00	2.37	1.62	1426	0.00	10.00	2.19	1.68	3.98	.046	.00
Prosocial behaviour	2069	0.00	10.00	6.90	1.95	643	0.00	10.00	6.15	1.99	1426	1.00	10.00	7.24	1.84	118.18	<.001	.06
General anxiety	2069	0.00	40.00	8.88	7.79	643	0.00	35.00	6.42	6.68	1426	0.00	40.00	9.99	8.00	108.97	<.001	.05
Depression	2069	0.00	16.00	4.51	4.01	643	0.00	16.00	3.33	3.43	1426	0.00	16.00	5.03	4.14	91.36	<.001	.04
Self-harm	2044	0.00	4.00	0.06	0.32	636	0.00	2.00	0.03	0.19	1408	0.00	4.00	0.07	0.37	11.70	.001	.00
Physical activity	2068	1.00	5.00	2.63	1.09	643	1.00	5.00	2.63	1.10	1425	1.00	5.00	2.63	1.09	0.01	.924	.00
Media use	2068	0.00	15.00	7.12	2.36	643	0.00	15.00	7.28	2.49	1425	0.00	15.00	7.05	2.30	2.86	.091	.00
Volunteering	2068	0.00	8.00	1.09	1.33	643	0.00	8.00	1.03	1.36	1425	0.00	8.00	1.12	1.31	2.37	.124	.00

Note: Raw scores are used.

[a] *F and p values are reported with Welch's correction (Welch, 1951) as the assumption of homogeneity of variance was violated for several variables.*

Supplementary Table 3. Results of F test; variance differences between T1 and T2. a) full sample; b) males and females

a)

Construct	All						
	T1		T2				
	M	*SD*	*M*	*SD*	*F*	*Direction*	*p*
Love and relationships	3.57	1.21	3.68	1.09	1.23	D	1.38E-06
Verbal peer victimisation	2.01	2.52	0.58	1.48	2.95	D	2.20E-16
Cyber peer victimisation	0.56	1.41	0.1	0.55	6.61	D	2.20E-16
Physical peer victimisation	0.19	0.79	0.04	0.39	3.97	D	2.20E-16
Achievement motivation	10.06	2.92	8.78	3.36	0.76	I	6.96E-11
Importance of Relationships	15.84	3.59	15.42	3.71	0.93	I	9.05E-02
Purpose in Life	3.45	0.81	3.47	0.81	1.00	D	9.65E-01
Healthcare	4.58	0.7	4.79	0.59	1.59	D	
Community satisfaction	3.6	0.75	3.55	0.7	1.13	D	3.41E-03
Attitudes towards money	3.05	0.91	3.16	0.9	1.02	D	6.39E-01
Alcohol frequency	1.9	0.99	2	1.27	0.62	I	2.20E-16
Alcohol quantity	2.41	1.26	1.86	1.26	0.62	I	2.20E-16
Alcohol (frequency x quantity)	4.67	3.68	4.07	4.26	0.76	I	2.06E-10
Ever smoked	0.54	0.5	0.47	0.5	1.00	D	9.94E-01
Smoking frequency	1.31	0.55	1.33	0.61	0.82	I	1.20E-01
Ever vaped	0.24	0.43	0.21	0.4	1.13	D	3.52E-03
Vaping frequency	1.71	1.17	2.92	1.23	0.91	I	5.63E-01
Ever used cannabis	0.48	0.5	0.47	0.5	1.00	D	9.47E-01

Cannabis smoking frequency	1.57	0.78	1.64	1.04	0.66	I	2.23E-03
Conduct problems	1.51	1.24	1.51	1.19	1.12	D	8.38E-03
Emotional problems	3.62	2.72	3.16	2.7	1.01	D	7.83E-01
Hyperactivity	3.3	2.2	4.22	2.26	0.94	I	1.75E-01
Peer problems	2.15	1.83	2.24	1.66	1.21	I	8.84E-06
Prosocial behaviour	7.7	1.89	6.9	1.95	0.94	I	1.17E-01
General anxiety	7.56	7.47	8.88	7.79	0.92	I	5.88E-02
Depression	4.39	4.12	4.51	4.01	1.06	D	1.53E-01
Self-harm	0.21	0.7	0.06	0.32	4.65	D	2.20E-16
Physical activity	2.8	1.09	2.63	1.09	1.00	D	9.80E-01
Media use	7.63	2.12	7.12	2.36	0.81	I	7.18E-07
Volunteering	2.8	2.1	1.09	1.33	2.51	D	2.20E-16

b)

**Males**							**Females**						
**T1**		**T2**					**T1**		**T2**				
M	*SD*	*M*	*SD*	*F statistic*	*Direction*	*p*	*M*	*SD*	*M*	*SD*	*F statistic*	*Direction*	*p*
3.39	1.19	3.57	1.1	1.17	D	4.01E-02	3.66	1.21	3.73	1.08	1.25	D	1.85E-05
2.43	2.73	0.87	1.85	2.25	D	2.20E-16	1.8	2.39	0.45	1.26	3.52	D	2.20E-16
0.52	1.4	0.12	0.62	4.76	D	2.20E-16	0.58	1.42	0.09	0.52	7.88	D	2.20E-16
0.32	0.99	0.05	0.48	3.99	D	2.20E-16	0.12	0.66	0.04	0.34	3.68	D	2.20E-16
9.88	3.16	8.85	3.46	0.83	I	1.62E-02	10.15	2.78	8.75	3.31	0.71	I	2.91E-11
14.59	3.96	14.09	3.98	0.97	I	7.13E-01	16.47	3.22	16.03	3.41	0.88	I	1.45E-02
3.38	0.84	3.44	0.83	1.03	D	7.28E-01	3.49	0.79	3.48	0.8	0.98	I	6.85E-01
4.48	0.79	4.7	0.71	1.49	D	2.28E-07	4.63	0.64	4.84	0.51	1.62	D	2.20E-16
3.59	0.73	3.52	0.67	1.17	D	4.49E-02	3.61	0.76	3.56	0.71	1.12	D	2.21E-02

3.14	0.93	3.27	0.94	0.96	I	6.27E-01	3	0.9	3.12	0.88	1.04	D	4.20E-01
2.04	1.02	2.07	1.33	0.60	I	5.53E-11	1.83	0.97	1.96	1.24	0.62	I	2.20E-16
2.41	1.27	2.03	1.27	0.66	I	3.43E-07	2.41	1.26	1.79	1.25	0.60	I	2.20E-16
5.01	3.86	4.52	4.53	0.75	I	2.78E-04	4.51	3.58	3.86	4.12	0.76	I	1.00E-07
0.53	0.5	0.49	0.5	1.00	I	9.95E-01	0.54	0.5	0.46	0.5	1.00	D	9.97E-01
1.39	0.63	1.26	0.55	1.16	D	5.57E-01	1.27	0.52	1.36	0.63	0.69	I	1.38E-02
0.27	0.44	0.25	0.43	1.08	D	3.18E-01	0.22	0.42	0.19	0.39	1.16	D	4.47E-03
1.9	1.27	3.04	1.11	1.33	D	4.05E-01	1.59	1.09	2.85	1.3	0.70	I	1.02E-01
0.52	0.5	0.51	0.5	1.00	I	9.79E-01	0.46	0.5	0.46	0.5	1.00	D	9.42E-01
1.71	0.89	1.73	1.08	0.67	I	7.46E-02	1.48	0.69	1.58	0.95	0.58	I	1.58E-03
1.53	1.22	1.5	1.24	1.04	D	6.53E-01	1.5	1.25	1.52	1.17	1.16	D	3.91E-03
2.66	2.45	2.03	2.29	1.18	D	3.33E-02	4.1	2.72	3.67	2.72	1.00	I	9.67E-01
3.33	2.21	4.05	2.25	0.97	I	6.66E-01	3.29	2.19	4.3	2.26	0.94	I	2.06E-01
2.14	1.73	2.37	1.62	1.13	D	1.20E-01	2.16	1.88	2.19	1.68	1.26	D	1.09E-05
7.01	2.01	6.15	1.99	1.01	D	9.29E-01	8.04	1.72	7.24	1.84	0.88	I	1.01E-02
5.96	6.82	6.42	6.68	1.07	D	4.19E-01	8.31	7.65	9.99	8	0.92	I	8.97E-02
3.65	3.68	3.33	3.43	1.16	D	5.34E-02	4.77	4.28	5.03	4.14	1.08	D	1.36E-01
0.16	0.62	0.03	0.19	10.94	D	2.20E-16	0.23	0.73	0.07	0.37	3.99	D	2.20E-16
2.9	1.1	2.63	1.1	1.02	D	7.89E-01	2.75	1.09	2.63	1.09	0.99	I	7.75E-01
7.89	2.2	7.28	2.49	0.77	I	5.05E-04	7.51	2.07	7.05	2.3	0.82	I	1.11E-04
2.64	2.03	1.03	1.36	2.26	D	2.20E-16	2.89	2.13	1.12	1.31	2.62	D	2.20E-16

Note: F- results of F test, also presents the ratio between T1 and T2 variances; D- decrease; I- increase

Supplementary Table 4. Descriptive statistics for T1 to T2 differences for one twin randomly selected from each pair.

Construct	All						Males						Females						ANOVA[a]		
	N	Min	Max	*M*	*SD*	*d*	*n*	Min	Max	*M*	*SD*	*d*	*n*	Min	Max	*M*	*SD*	d	Sex	*p*	R^2
Love and relationships	1832	-4.00	4.00	0.08	1.24	0.06	539	-4.00	4.00	0.16	1.24	0.13	1293	-4.00	4.00	0.05	1.23	0.04	3.05	.081	.00
Verbal peer victimisation	1760	-8.00	8.00	-1.44	2.48	0.58	516	-8.00	8.00	-1.62	2.76	0.59	1244	-8.00	8.00	-1.37	2.34	0.59	3.13	.077	.00
Cyber peer victimisation	1760	-8.00	5.00	-0.43	1.35	0.31	516	-8.00	5.00	-0.35	1.38	0.25	1244	-8.00	5.00	-0.46	1.35	0.34	2.32	.128	.00
Physical peer victimisation	1760	-8.00	8.00	-0.13	0.83	0.15	516	-8.00	6.00	-0.26	1.10	0.23	1244	-7.00	8.00	-0.07	0.67	0.11	12.05	.001	.01
Achievement motivation	1872	-11.00	9.00	-1.32	2.79	0.47	551	-11.00	8.00	-1.13	2.73	0.42	1321	-11.00	9.00	-1.39	2.81	0.50	3.51	.061	.00
Importance of Relationships	1872	-15.00	15.00	-0.49	3.11	0.16	551	-14.00	15.00	-0.69	3.59	0.19	1321	-15.00	14.00	-0.40	2.88	0.14	2.70	.101	.00
Purpose in Life	1871	-3.80	2.80	0.01	0.65	0.01	551	-2.00	2.20	0.03	0.66	0.04	1320	-3.80	2.80	0.00	0.64	0.00	0.67	.412	.00
Healthcare	1869	-4.00	4.00	0.23	0.74	0.32	550	-4.00	4.00	0.22	0.79	0.28	1319	-4.00	4.00	0.24	0.72	0.33	0.23	.629	.00
Community satisfaction	1865	-3.60	3.80	-0.05	0.78	0.07	551	-2.40	2.40	-0.07	0.75	0.09	1314	-3.60	3.80	-0.05	0.79	0.06	0.41	.522	.00
Attitudes towards money	1831	-3.00	4.00	0.12	0.99	0.12	546	-3.00	3.00	0.09	1.01	0.09	1285	-3.00	4.00	0.13	0.98	0.13	0.49	.486	.00

Alcohol frequency	1692	-4.00	4.00	0.21	1.10	0.19	491	-4.00	4.00	0.11	1.14	0.09	1201	-4.00	4.00	0.25	1.08	0.23	5.26	.022	.00
Alcohol quantity	1509	-4.00	4.00	-0.67	1.56	0.43	454	-4.00	4.00	-0.52	1.51	0.34	1055	-4.00	4.00	-0.74	1.58	0.47	4.44	.036	.00
Alcohol (frequency x quantity)	1706	-15.00	16.00	-0.67	4.24	0.16	494	-12.00	12.00	-0.59	4.25	0.14	1212	-15.00	16.00	-0.70	4.23	0.17	0.00	.978	.00
Ever smoked	1780	-1.00	1.00	-0.07	0.36	0.19	516	-1.00	1.00	-0.04	0.36	0.11	1264	-1.00	1.00	-0.08	0.36	0.22	4.02	.045	.00
Smoking frequency	134	-2.00	3.00	0.11	0.63	0.18	25	-2.00	1.00	-0.12	0.53	0.23	109	-1.00	3.00	0.17	0.65	0.26	5.46	.024	.03
Ever vaped	1782	-1.00	1.00	-0.04	0.36	0.10	516	-1.00	1.00	-0.04	0.37	0.10	1266	-1.00	1.00	-0.03	0.35	0.10	0.01	.915	.00
Vaping frequency	36	-1.00	3.00	0.64	0.99	0.65	14	-1.00	3.00	0.50	1.02	0.49	22	0.00	3.00	0.73	0.98	0.74	0.44	.514	.01
Ever used cannabis	1750	-1.00	1.00	-0.02	0.33	0.05	500	-1.00	1.00	-0.02	0.30	0.05	1250	-1.00	1.00	-0.02	0.34	0.05	0.00	1.00	.00
Cannabis smoking frequency	87	-2.00	3.00	-0.30	0.85	0.35	34	-2.00	1.00	-0.29	0.68	0.44	53	-2.00	3.00	-0.30	0.95	0.32	0.00	.965	.00
Conduct problems	1838	-7.00	5.00	-0.01	1.39	0.01	545	-6.00	5.00	-0.06	1.39	0.04	1293	-7.00	5.00	0.01	1.39	0.01	1.06	.304	.00
Emotional problems	1838	-9.00	8.00	-0.44	2.45	0.18	545	-8.00	7.00	-0.59	2.11	0.28	1293	-9.00	8.00	-0.38	2.58	0.15	3.18	.075	.00
Hyperactivity	1838	-7.00	8.00	0.98	2.34	0.42	545	-5.00	8.00	0.83	2.23	0.37	1293	-7.00	8.00	1.05	2.38	0.44	3.52	.061	.00

Peer problems	1838	-6.00	5.00	0.07	1.58	0.05	545	-5.00	4.00	0.18	1.60	0.11	1293	-6.00	5.00	0.03	1.58	0.02	3.40	.066	.00
Prosocial behaviour	1838	-8.00	8.00	-0.84	1.92	0.44	545	-8.00	6.00	-0.89	2.03	0.44	1293	-7.00	8.00	-0.81	1.87	0.44	0.57	.450	.00
General anxiety	1774	-32.00	34.00	1.38	6.90	0.20	513	-32.00	26.00	0.48	6.14	0.08	1261	-27.00	34.00	1.74	7.15	0.24	13.88	.000	.01
Depression	1837	-16.00	15.00	0.20	3.76	0.05	545	-16.00	12.00	-0.21	3.32	0.06	1292	-13.00	15.00	0.37	3.92	0.09	10.51	.001	.00
Self-harm	1742	-4.00	4.00	-0.14	0.66	0.21	504	-4.00	2.00	-0.13	0.62	0.22	1238	-4.00	4.00	-0.14	0.67	0.21	0.07	.793	.00
Physical activity	1846	-4.00	4.00	-0.17	1.12	0.15	545	-3.50	3.00	-0.28	1.08	0.26	1301	-4.00	4.00	-0.12	1.14	0.11	8.41	.004	.00
Media use	1841	-10.00	10.00	-0.48	2.29	0.21	545	-10.00	6.00	-0.59	2.21	0.26	1296	-10.00	10.00	-0.43	2.32	0.18	1.86	.173	.00
Volunteering	1837	-8.00	6.00	-1.80	2.13	0.84	545	-8.00	5.00	-1.77	2.10	0.84	1292	-8.00	6.00	-1.81	2.15	0.84	0.13	.715	.00

Note: Raw scores are used.

[a] F and p values are reported with Welch's correction (Welch, 1951) as the assumption of homogeneity of variance was violated for several variables.

Supplementary Table 5. Descriptive statistics for T1 for one twin randomly selected from each pair broken down by zygosity.

	**T1**									
**Construct**	**MZ**					**DZ**				
	**n**	**Min**	**Max**	**M**	**SD**	**n**	**Min**	**Max**	**M**	**SD**
Love and relationships	987	1.00	5.00	3.60	1.18	1544	1.00	5.00	3.55	1.23
Verbal peer victimisation	911	0.00	8.00	1.78	2.39	1423	0.00	8.00	2.15	2.59
Cyber peer victimisation	911	0.00	8.00	0.52	1.35	1423	0.00	8.00	0.58	1.45

Physical peer victimisation	911	0.00	8.00	0.16	0.73	1423	0.00	8.00	0.20	0.81
Achievement motivation	986	0.00	16.00	10.08	2.91	1550	0.00	16.00	10.05	2.92
Importance of Relationships	986	0.00	20.00	15.91	3.56	1550	0.00	20.00	15.80	3.61
Purpose in Life	985	1.00	5.00	3.51	0.77	1550	1.00	5.00	3.42	0.83
Healthcare	984	1.00	5.00	4.59	0.68	1548	1.00	5.00	4.58	0.71
Community satisfaction	985	1.40	5.00	3.60	0.75	1537	1.00	5.00	3.60	0.75
Attitudes towards money	970	1.00	5.00	3.05	0.90	1504	1.00	5.00	3.05	0.92
Alcohol frequency	882	0.00	4.00	1.83	0.99	1388	0.00	4.00	1.94	0.99
Alcohol quantity	796	0.00	4.00	2.44	1.26	1274	0.00	4.00	2.39	1.27
Alcohol (frequency x quantity)	882	0.00	16.00	4.57	3.69	1388	0.00	16.00	4.74	3.68
Ever smoked	928	0.00	1.00	0.51	0.50	1445	0.00	1.00	0.55	0.50
Smoking frequency	93	1.00	4.00	1.26	0.57	170	1.00	3.00	1.34	0.54
Ever vaped	929	0.00	1.00	0.23	0.42	1447	0.00	1.00	0.24	0.43
Vaping frequency	102	1.00	4.00	1.74	1.21	175	1.00	4.00	1.69	1.15
Ever used cannabis	915	0.00	1.00	0.45	0.50	1425	0.00	1.00	0.50	0.50
Cannabis smoking frequency	143	1.00	5.00	1.50	0.73	253	1.00	5.00	1.61	0.81

Conduct problems	972	0.00	8.00	1.52	1.20	1536	0.00	8.00	1.51	1.27
Emotional problems	972	0.00	10.00	3.60	2.68	1536	0.00	10.00	3.63	2.75
Hyperactivity	972	0.00	10.00	3.22	2.15	1536	0.00	10.00	3.35	2.23
Peer problems	972	0.00	10.00	2.14	1.83	1536	0.00	9.00	2.16	1.84
Prosocial behaviour	972	0.00	10.00	7.67	1.90	1536	0.00	10.00	7.72	1.88
General anxiety	922	0.00	40.00	7.29	7.41	1444	0.00	40.00	7.74	7.52
Depression	971	0.00	16.00	4.29	4.12	1535	0.00	16.00	4.46	4.13
Self-harm	916	0.00	4.00	0.19	0.66	1439	0.00	4.00	0.22	0.72
Physical activity	982	1.00	5.00	2.79	1.08	1529	1.00	5.00	2.81	1.10
Media use	980	0.00	15.00	7.55	2.11	1525	0.00	15.00	7.69	2.12
Volunteering	970	0.00	8.00	2.81	2.10	1535	0.00	8.00	2.81	2.10

Note: Raw scores are used.

Supplementary Table 6. Descriptive statistics for T2 for one twin randomly selected from each pair broken down by zygosity.

	**T2**									
**Construct**	**MZ**					**DZ**				
	**n**	**Min**	**Max**	**M**	**SD**	**n**	**Min**	**Max**	**M**	**SD**
Love and relationships	762	1.00	5.00	3.68	1.07	1162	1.00	5.00	3.67	1.11
Verbal peer victimisation	778	0.00	8.00	0.52	1.36	1182	0.00	8.00	0.61	1.54
Cyber peer victimisation	778	0.00	5.00	0.09	0.47	1182	0.00	8.00	0.11	0.60

Physical peer victimisation	778	0.00	6.00	0.04	0.36	1182	0.00	8.00	0.04	0.42
Achievement motivation	778	0.00	16.00	8.71	3.31	1183	0.00	16.00	8.80	3.37
Importance of Relationships	779	0.00	20.00	15.27	3.82	1183	1.00	20.00	15.52	3.66
Purpose in Life	779	1.00	5.00	3.53	0.77	1183	1.00	5.00	3.43	0.83
Healthcare	779	1.00	5.00	4.78	0.63	1183	1.00	5.00	4.82	0.50
Community satisfaction	779	1.40	5.00	3.54	0.69	1183	1.00	5.00	3.55	0.71
Attitudes towards money	779	1.00	5.00	3.14	0.88	1183	1.00	5.00	3.17	0.91
Alcohol frequency	774	0.00	4.00	1.93	1.26	1178	0.00	4.00	2.06	1.26
Alcohol quantity	619	0.00	4.00	1.84	1.25	972	0.00	4.00	1.86	1.26
Alcohol (frequency x quantity)	774	0.00	16.00	3.89	4.17	1177	0.00	16.00	4.17	4.26
Ever smoked	775	0.00	1.00	0.44	0.50	1177	0.00	1.00	0.48	0.50
Smoking frequency	85	1.00	3.00	1.21	0.49	150	1.00	4.00	1.38	0.66
Ever vaped	775	0.00	1.00	0.18	0.39	1178	0.00	1.00	0.21	0.41
Vaping frequency	34	1.00	4.00	2.65	1.32	41	1.00	4.00	3.15	1.11
Ever used cannabis	764	0.00	1.00	0.43	0.50	1169	0.00	1.00	0.49	0.50
Cannabis smoking frequency	55	1.00	4.00	1.60	0.94	75	1.00	5.00	1.56	1.00

Conduct problems	775	0.00	7.00	1.49	1.11	1175	0.00	7.00	1.49	1.21
Emotional problems	775	0.00	10.00	3.06	2.66	1175	0.00	10.00	3.23	2.73
Hyperactivity	775	0.00	10.00	4.04	2.18	1175	0.00	10.00	4.32	2.31
Peer problems	775	0.00	8.00	2.25	1.63	1175	0.00	10.00	2.20	1.69
Prosocial behaviour	775	1.00	10.00	6.84	1.97	1175	0.00	10.00	6.97	1.94
General anxiety	775	0.00	37.00	8.22	7.37	1175	0.00	40.00	9.28	8.01
Depression	775	0.00	16.00	4.24	3.75	1175	0.00	16.00	4.68	4.13
Self-harm	769	0.00	4.00	0.05	0.29	1156	0.00	4.00	0.06	0.35
Physical activity	775	1.00	5.00	2.68	1.08	1174	1.00	5.00	2.60	1.10
Media use	775	0.00	15.00	7.15	2.38	1174	0.00	15.00	7.12	2.35
Volunteering	775	0.00	8.00	1.04	1.30	1174	0.00	8.00	1.12	1.34

Note: Raw scores are used.

Supplementary Table 7. Descriptive statistics for T1 to T2 differences for one twin randomly selected from each pair broken down by zygosity.

	T2-T1 difference scores											
**Construct**	**MZ**						**DZ**					
	**n**	**Min**	**Max**	**M**	**SD**	***d*[a]**	**n**	**Min**	**Max**	**M**	**SD**	***d*[a]**
Love and relationships	726	-4.00	4.00	0.05	1.21	0.04	1095	-4.00	4.00	0.10	1.26	0.08
Verbal peer victimisation	700	-8.00	8.00	-1.31	2.35	0.56	1050	-8.00	8.00	-1.53	2.56	0.60
Cyber peer victimisation	700	-8.00	5.00	-0.39	1.30	0.30	1050	-8.00	5.00	-0.44	1.38	0.32
Physical peer victimisation	700	-6.00	6.00	-0.11	0.75	0.15	1050	-8.00	8.00	-0.14	0.88	0.16

Achievement motivation	746	-9.00	8.00	-1.33	2.70	0.49	1115	-11.00	9.00	-1.31	2.84	0.46
Importance of Relationships	746	-14.00	14.00	-0.57	3.02	0.19	1115	-15.00	15.00	-0.43	3.17	0.14
Purpose in Life	745	-2.00	2.40	0.03	0.66	0.05	1115	-3.80	2.80	-0.01	0.64	0.01
Healthcare	744	-4.00	4.00	0.21	0.77	0.28	1114	-4.00	4.00	0.24	0.71	0.34
Community satisfaction	741	-2.80	2.60	-0.06	0.75	0.08	1113	-3.60	3.80	-0.05	0.79	0.06
Attitudes towards money	732	-3.00	3.00	0.10	0.94	0.10	1089	-3.00	4.00	0.13	1.03	0.13
Alcohol frequency	669	-4.00	4.00	0.20	1.09	0.18	1014	-4.00	4.00	0.21	1.10	0.19
Alcohol quantity	583	-4.00	4.00	-0.69	1.54	0.45	918	-4.00	4.00	-0.67	1.58	0.42
Alcohol (frequency x quantity)	673	-15.00	16.00	-0.69	4.23	0.16	1023	-14.00	16.00	-0.66	4.25	0.16
Ever smoked	711	-1.00	1.00	-0.06	0.37	0.17	1058	-1.00	1.00	-0.07	0.36	0.20
Smoking frequency	48	-2.00	2.00	0.06	0.60	0.10	84	-1.00	3.00	0.13	0.65	0.20
Ever vaped	711	-1.00	1.00	-0.04	0.36	0.12	1060	-1.00	1.00	-0.03	0.36	0.08
Vaping frequency	17	-1.00	3.00	0.47	0.94	0.50	19	0.00	3.00	0.79	1.03	0.77
Ever used cannabis	692	-1.00	1.00	0.00	0.33	0.01	1048	-1.00	1.00	-0.03	0.32	0.08
Cannabis smoking frequency	35	-2.00	2.00	-0.26	0.82	0.31	52	-2.00	3.00	-0.33	0.88	0.37
Conduct problems	727	-6.00	4.00	-0.04	1.35	0.03	1101	-7.00	5.00	0.01	1.42	0.00

Emotional problems	727	-9.00	7.00	-0.49	2.45	0.20	1101	-8.00	8.00	-0.42	2.45	0.17
Hyperactivity	727	-7.00	8.00	0.92	2.30	0.40	1101	-7.00	8.00	1.02	2.36	0.43
Peer problems	727	-5.00	4.00	0.13	1.60	0.08	1101	-6.00	5.00	0.03	1.57	0.02
Prosocial behaviour	727	-7.00	6.00	-0.82	1.91	0.43	1101	-8.00	8.00	-0.84	1.91	0.44
General anxiety	707	-27.00	30.00	1.02	6.96	0.15	1056	-32.00	34.00	1.58	6.85	0.23
Depression	726	-15.00	15.00	0.09	3.80	0.02	1101	-16.00	14.00	0.24	3.70	0.06
Self-harm	697	-4.00	3.00	-0.12	0.57	0.21	1034	-4.00	4.00	-0.15	0.71	0.22
Physical activity	734	-3.33	4.00	-0.11	1.10	0.10	1101	-4.00	3.50	-0.21	1.13	0.19
Media use	734	-10.00	10.00	-0.38	2.36	0.16	1096	-10.00	7.00	-0.54	2.24	0.24
Volunteering	726	-8.00	5.00	-1.84	2.03	0.91	1101	-8.00	6.00	-1.78	2.19	0.82

Note: Raw scores are used.

[a]Cohen's d for paired samples are reported.

Supplementary Table 8. Descriptive statistics at T1 for the twin that was not randomly selected from each pair in the main analyses.

Construct	All					Males					Females					ANOVA[a]		
	N	Min	Max	*M*	*SD*	*n*	Min	Max	*M*	*SD*	*n*	Min	Max	*M*	*SD*	Sex	*p*	R^2
Love and relationships	2521	1.00	5.00	3.56	1.21	858	1.00	5.00	3.40	1.21	1663	1.00	5.00	3.65	1.20	23.70	<.001	.01
Verbal peer victimisation	2355	0.00	8.00	2.03	2.51	793	0.00	8.00	2.58	2.75	1562	0.00	8.00	1.75	2.33	52.65	<.001	.02
Cyber peer victimisation	2354	0.00	8.00	0.54	1.34	793	0.00	8.00	0.52	1.38	1561	0.00	8.00	0.55	1.32	0.34	.560	.00
Physical peer victimisation	2354	0.00	8.00	0.22	0.86	793	0.00	8.00	0.42	1.21	1561	0.00	7.00	0.12	0.60	40.97	<.001	.03

Achievement motivation	2533	0.00	16.00	10.16	2.90	864	0.00	16.00	10.28	3.08	1669	0.00	16.00	10.10	2.80	2.07	.151	.00
Importance of Relationships	2533	0.00	20.00	16.00	3.43	864	0.00	20.00	15.02	3.73	1669	0.00	20.00	16.51	3.14	101.10	<.001	.04
Purpose in Life	2532	1.00	5.00	3.45	0.81	864	1.00	5.00	3.37	0.82	1668	1.00	5.00	3.49	0.80	12.06	.001	.00
Healthcare	2527	1.00	5.00	4.57	0.71	862	1.00	5.00	4.45	0.81	1665	1.00	5.00	4.64	0.65	32.85	<.001	.01
Community satisfaction	2509	1.00	5.00	3.62	0.75	853	1.00	5.00	3.58	0.73	1656	1.00	5.00	3.64	0.75	3.38	.066	.00
Attitudes towards money	2436	1.00	5.00	3.04	0.91	825	1.00	5.00	3.16	0.92	1611	1.00	5.00	2.97	0.89	24.86	<.001	.01
Alcohol frequency	2295	0.00	4.00	1.91	1.01	774	0.00	4.00	2.13	1.02	1521	0.00	4.00	1.80	0.99	56.10	<.001	.02
Alcohol quantity	2082	0.00	4.00	2.49	1.26	723	0.00	4.00	2.63	1.25	1359	0.00	4.00	2.42	1.26	13.17	<.001	.01
Alcohol (frequency x quantity)	2294	0.00	16.00	4.90	3.87	773	0.00	16.00	5.68	4.00	1521	0.00	16.00	4.50	3.74	47.12	<.001	.02
Ever smoked	2389	0.00	1.00	0.56	0.50	802	0.00	1.00	0.58	0.49	1587	0.00	1.00	0.55	0.50	2.62	.106	.00
Smoking frequency	302	1.00	4.00	1.30	0.52	114	1.00	4.00	1.35	0.58	188	1.00	3.00	1.27	0.48	1.73	.190	.01
Ever vaped	2388	0.00	1.00	0.25	0.43	799	0.00	1.00	0.30	0.46	1589	0.00	1.00	0.22	0.42	16.39	<.001	.01
Vaping frequency	303	1.00	4.00	1.66	1.16	108	1.00	4.00	1.79	1.25	195	1.00	4.00	1.59	1.10	1.88	.172	.01

Ever used cannabis	2364	0.00	1.00	0.49	0.50	793	0.00	1.00	0.56	0.50	1571	0.00	1.00	0.45	0.50	24.86	<.001	.01
Cannabis smoking frequency	415	1.00	5.00	1.49	0.71	184	1.00	5.00	1.58	0.76	231	1.00	5.00	1.42	0.65	4.92	.027	.01
Conduct problems	2503	0.00	9.00	1.50	1.28	853	0.00	9.00	1.61	1.31	1650	0.00	8.00	1.44	1.27	9.37	.002	.00
Emotional problems	2503	0.00	10.00	3.59	2.67	853	0.00	10.00	2.81	2.51	1650	0.00	10.00	4.00	2.66	121.61	<.001	.04
Hyperactivity	2503	0.00	10.00	3.20	2.15	853	0.00	10.00	3.42	2.13	1650	0.00	10.00	3.08	2.16	14.58	<.001	.01
Peer problems	2503	0.00	10.00	2.14	1.79	853	0.00	8.00	2.13	1.75	1650	0.00	10.00	2.14	1.81	0.02	.904	.00
Prosocial behaviour	2503	0.00	10.00	7.71	1.84	853	0.00	10.00	7.11	1.94	1650	1.00	10.00	8.02	1.70	133.50	<.001	.05
General anxiety	2386	0.00	40.00	7.40	7.36	798	0.00	37.00	5.84	6.56	1588	0.00	40.00	8.18	7.60	60.90	<.001	.02
Depression	2502	0.00	16.00	4.45	4.11	853	0.00	16.00	3.89	3.76	1649	0.00	16.00	4.74	4.25	26.14	<.001	.01
Self-harm	2383	0.00	4.00	0.19	0.66	799	0.00	4.00	0.11	0.52	1584	0.00	4.00	0.22	0.72	18.63	<.001	.01
Physical activity	2492	1.00	5.00	2.83	1.06	841	1.00	5.00	3.01	1.11	1651	1.00	5.00	2.74	1.03	32.93	<.001	.01
Media use	2481	0.00	15.00	7.63	2.12	836	0.00	14.00	8.01	2.12	1645	0.00	15.00	7.44	2.10	40.44	<.001	.02
Volunteering	2502	0.00	8.00	2.87	2.10	853	0.00	8.00	2.75	2.08	1649	0.00	8.00	2.93	2.11	4.11	.043	.00

Note: Raw scores are used.

[a] F and p values are reported with Welch's correction (Welch, 1951) as the assumption of homogeneity of variance was violated for several variables.

Supplementary Table 9. Descriptive statistics at T2 for twin two randomly selected from each pair.

Construct	All					Males					Females					ANOVA[a]		
	N	Min	Max	*M*	*SD*	*n*	Min	Max	*M*	*SD*	*n*	Min	Max	*M*	*SD*	Sex	*p*	R^2
Love and relationships	1966	1.00	5.00	3.66	1.10	612	1.00	5.00	3.56	1.13	1354	1.00	5.00	3.71	1.09	7.37	.007	.00
Verbal peer victimisation	2005	0.00	8.00	0.54	1.38	619	0.00	8.00	0.79	1.77	1386	0.00	8.00	0.43	1.15	20.60	<.001	.01
Cyber peer victimisation	2005	0.00	8.00	0.11	0.60	619	0.00	8.00	0.12	0.67	1386	0.00	8.00	0.10	0.56	0.71	.400	.00
Physical peer victimisation	2004	0.00	6.67	0.03	0.28	618	0.00	5.00	0.04	0.31	1386	0.00	6.67	0.03	0.26	0.43	.512	.00
Achievement motivation	1992	0.00	16.00	8.64	3.33	615	0.00	16.00	9.00	3.43	1377	0.00	16.00	8.49	3.27	9.77	.002	.01
Importance of Relationships	1992	0.00	20.00	15.43	3.69	614	0.00	20.00	14.59	3.90	1378	0.00	20.00	15.81	3.53	44.23	<.001	.02
Purpose in Life	1997	1.00	5.00	3.51	0.78	617	1.00	5.00	3.48	0.82	1380	1.00	5.00	3.52	0.76	1.29	.257	.00
Healthcare	1997	1.00	5.00	4.80	0.58	617	1.00	5.00	4.71	0.69	1380	1.00	5.00	4.83	0.51	16.61	<.001	.01
Community satisfaction	1997	1.00	5.00	3.56	0.69	617	1.20	5.00	3.51	0.68	1380	1.00	5.00	3.58	0.70	4.29	.039	.00
Attitudes towards money	1997	1.00	5.00	3.18	0.92	617	1.00	5.00	3.24	0.99	1380	1.00	5.00	3.15	0.89	4.56	.033	.00
Alcohol frequency	1993	0.00	4.00	2.00	1.24	615	0.00	4.00	2.12	1.28	1378	0.00	4.00	1.95	1.22	7.91	.005	.00

Alcohol quantity	1628	0.00	4.00	1.87	1.24	507	0.00	4.00	2.12	1.26	1121	0.00	4.00	1.76	1.21	28.90	<.001	.02
Alcohol (frequency x quantity)	1991	0.00	16.00	4.03	4.10	614	0.00	16.00	4.77	4.46	1377	0.00	16.00	3.70	3.88	26.21	<.001	.01
Ever smoked	1983	0.00	1.00	0.47	0.50	613	0.00	1.00	0.51	0.50	1370	0.00	1.00	0.45	0.50	4.53	.033	.00
Smoking frequency	259	1.00	3.00	1.28	0.53	79	1.00	3.00	1.29	0.54	180	1.00	3.00	1.27	0.53	0.07	.793	.00
Ever vaped	1985	0.00	1.00	0.20	0.40	614	0.00	1.00	0.26	0.44	1371	0.00	1.00	0.18	0.38	16.82	<.001	.01
Vaping frequency	79	1.00	4.00	3.10	1.14	36	2.00	4.00	3.50	0.78	43	1.00	4.00	2.77	1.29	9.71	.003	.10
Ever used cannabis	1970	0.00	1.00	0.46	0.50	609	0.00	1.00	0.54	0.50	1361	0.00	1.00	0.42	0.49	22.70	<.001	.01
Cannabis smoking frequency	116	1.00	5.00	1.58	0.89	50	1.00	5.00	1.74	1.01	66	1.00	5.00	1.45	0.77	2.79	.098	.03
Conduct problems	1989	0.00	7.00	1.46	1.17	615	0.00	6.00	1.43	1.07	1374	0.00	7.00	1.47	1.21	0.43	.513	.00
Emotional problems	1989	0.00	10.00	3.06	2.61	615	0.00	10.00	2.09	2.33	1374	0.00	10.00	3.49	2.62	141.97	<.001	.06
Hyperactivity	1989	0.00	10.00	4.08	2.19	615	0.00	10.00	4.00	2.31	1374	0.00	10.00	4.12	2.14	1.20	.273	.00
Peer problems	1989	0.00	10.00	2.21	1.65	615	0.00	10.00	2.36	1.58	1374	0.00	9.00	2.14	1.68	8.02	.005	.00
Prosocial behaviour	1989	0.00	10.00	6.95	2.03	615	0.00	10.00	6.17	2.09	1374	1.00	10.00	7.30	1.89	131.69	<.001	.07

General anxiety	1989	0.00	40.00	8.50	7.39	615	0.00	37.00	6.19	6.47	1374	0.00	40.00	9.53	7.54	101.61	<.001	.04
Depression	1989	0.00	16.00	4.20	3.89	615	0.00	16.00	3.33	3.36	1374	0.00	16.00	4.59	4.04	52.86	<.001	.02
Self-harm	1968	0.00	4.00	0.04	0.24	605	0.00	4.00	0.02	0.21	1363	0.00	4.00	0.04	0.26	4.93	.027	.00
Physical activity	1986	1.00	5.00	2.66	1.10	615	1.00	5.00	2.61	1.12	1371	1.00	5.00	2.68	1.09	1.90	.169	.00
Media use	1986	0.00	15.00	6.98	2.32	615	0.00	15.00	7.21	2.45	1371	0.00	14.00	6.87	2.24	8.34	.004	.00
Volunteering	1986	0.00	8.00	1.10	1.30	615	0.00	8.00	1.00	1.27	1371	0.00	8.00	1.15	1.31	5.95	.015	.00

[a] F and p values are reported with Welch's correction (Welch, 1951) as the assumption of homogeneity of variance was violated for several variables.

Supplementary Table 10. Descriptive statistics for T1 to T2 differences for twin two randomly selected from each pair.

**Construct**	**All**						**Males**						**Females**						**ANOVA**[a]		
	N	Min	Max	*M*	*SD*	*d*	*n*	Min	Max	*M*	*SD*	*d*	*n*	Min	Max	*M*	*SD*	d	Sex	*p*	R^2
Love and relationships	1853	-4.00	4.00	0.07	1.20	0.06	573	-3.33	4.00	0.13	1.24	0.10	1280	-4.00	4.00	0.04	1.19	0.04	1.93	.165	.00
Verbal peer victimisation	1778	-8.00	8.00	-1.44	2.44	0.59	536	-8.00	8.00	-1.70	2.68	0.63	1242	-8.00	8.00	-1.33	2.31	0.57	7.68	.006	.00
Cyber peer victimisation	1778	-8.00	8.00	-0.40	1.31	0.30	536	-8.00	8.00	-0.34	1.36	0.25	1242	-8.00	6.00	-0.42	1.28	0.33	1.43	.232	.00
Physical peer victimisation	1776	-7.00	5.00	-0.17	0.84	0.21	535	-6.00	5.00	-0.35	1.18	0.29	1241	-7.00	3.00	-0.10	0.62	0.15	21.78	<.001	.02

Achievement motivation	1875	-14.00	10.00	-1.52	2.93	0.52	577	-12.00	10.00	-1.35	2.97	0.46	1298	-14.00	8.00	-1.59	2.91	0.55	2.60	.107	.00
Importance of Relationships	1875	-16.00	13.00	-0.61	3.06	0.20	576	-13.00	10.00	-0.50	3.38	0.15	1299	-16.00	13.00	-0.66	2.91	0.23	0.95	.330	.00
Purpose in Life	1879	-2.40	2.80	0.04	0.67	0.06	579	-2.20	2.40	0.09	0.70	0.12	1300	-2.40	2.80	0.02	0.65	0.03	3.45	.064	.00
Healthcare	1877	-4.00	4.00	0.21	0.73	0.28	578	-4.00	4.00	0.26	0.80	0.32	1299	-4.00	4.00	0.18	0.70	0.26	3.57	.059	.00
Community satisfaction	1873	-3.00	3.60	-0.07	0.76	0.10	574	-2.80	2.20	-0.05	0.74	0.07	1299	-3.00	3.60	-0.08	0.77	0.11	0.69	.406	.00
Attitudes towards money	1823	-4.00	4.00	0.16	1.02	0.15	555	-4.00	4.00	0.09	1.16	0.07	1268	-3.00	3.00	0.19	0.94	0.20	3.15	.076	.00
Alcohol frequency	1715	-4.00	4.00	0.20	1.05	0.19	518	-4.00	3.00	0.06	1.04	0.05	1197	-4.00	4.00	0.26	1.05	0.25	14.25	<.001	.01
Alcohol quantity	1403	-4.00	4.00	-0.56	1.45	0.39	432	-4.00	4.00	-0.51	1.48	0.34	971	-4.00	4.00	-0.58	1.43	0.41	0.78	.378	.00
Alcohol (frequency x quantity)	1714	-16.00	16.00	-0.58	4.00	0.14	517	-16.00	13.00	-0.76	4.29	0.18	1197	-16.00	16.00	-0.50	3.87	0.13	1.46	.228	.00
Ever smoked	1788	-1.00	1.00	-0.08	0.37	0.22	539	-1.00	1.00	-0.06	0.34	0.19	1249	-1.00	1.00	-0.09	0.38	0.24	2.15	.143	.00
Smoking frequency	154	-1.00	2.00	0.04	0.55	0.07	48	-1.00	1.00	0.02	0.64	0.03	106	-1.00	2.00	0.05	0.50	0.09	0.06	.801	.00
Ever vaped	1791	-1.00	1.00	-0.04	0.36	0.12	540	-1.00	1.00	-0.06	0.38	0.16	1251	-1.00	1.00	-0.03	0.35	0.10	1.95	.163	.00

Vaping frequency	52	-5.00	3.00	0.75	1.57	0.48	23	-2.00	3.00	0.96	1.36	0.70	29	-3.00	3.00	0.59	1.72	0.34	0.75	.391	.01
Ever used cannabis	1757	-1.00	1.00	-0.02	0.34	0.05	528	-1.00	1.00	-0.02	0.33	0.06	1229	-1.00	1.00	-0.02	0.35	0.04	0.04	.841	.00
Cannabis smoking frequency	74	-2.00	4.00	-0.07	0.96	0.07	33	-2.00	4.00	0.15	1.20	0.13	41	-2.00	1.00	-0.24	0.66	0.37	2.87	.097	.04
Conduct problems	1858	-8.00	6.00	-0.01	1.43	0.01	571	-8.00	4.00	-0.16	1.44	0.11	1287	-6.00	6.00	0.06	1.43	0.04	8.99	.003	.00
Emotional problems	1858	-9.00	10.00	-0.55	2.51	0.22	571	-8.00	7.00	-0.69	2.38	0.29	1287	-9.00	10.00	-0.48	2.57	0.19	2.80	.095	.00
Hyperactivity	1858	-7.00	9.00	1.01	2.31	0.43	571	-7.00	7.00	0.72	2.34	0.31	1287	-6.00	9.00	1.13	2.29	0.49	12.52	<.001	.01
Peer problems	1858	-7.00	5.00	0.09	1.62	0.06	571	-5.00	5.00	0.29	1.63	0.18	1287	-7.00	5.00	0.01	1.61	0.00	11.97	.001	.01
Prosocial behaviour	1858	-8.00	8.00	-0.81	1.96	0.41	571	-8.00	8.00	-1.00	2.09	0.48	1287	-8.00	6.00	-0.73	1.89	0.39	6.86	.009	.00
General anxiety	1789	-31.00	37.00	1.04	6.75	0.15	537	-24.00	29.00	0.36	5.57	0.06	1252	-31.00	37.00	1.33	7.18	0.18	9.48	.002	.00
Depression	1857	-15.00	15.00	-0.19	3.87	0.05	571	-13.00	14.00	-0.54	3.57	0.15	1286	-15.00	15.00	-0.04	3.98	0.01	7.19	.007	.00
Self-harm	1771	-4.00	2.00	-0.15	0.62	0.24	529	-4.00	1.00	-0.11	0.52	0.20	1242	-4.00	2.00	-0.17	0.66	0.26	4.63	.032	.00
Physical activity	1852	-3.83	3.50	-0.15	1.11	0.14	566	-3.83	3.50	-0.36	1.13	0.31	1286	-3.67	3.33	-0.06	1.09	0.06	26.55	<.001	.01
Media use	1845	-10.00	8.00	-0.62	2.25	0.27	563	-10.00	8.00	-0.80	2.32	0.35	1282	-10.00	8.00	-0.54	2.22	0.24	5.29	.022	.00
Volunteering	1854	-8.00	7.00	-1.86	2.15	0.86	571	-8.00	6.00	-1.83	2.12	0.86	1283	-8.00	7.00	-1.87	2.17	0.86	0.10	.753	.00

[a] *F and p values are reported with Welch's correction (Welch, 1951) as the assumption of homogeneity of variance was violated for several variables.*

Supplementary Table 11. Phenotypic correlations between T1 and T2 for the whole sample and split by males and females.

Construct	All			Males			Females		
	r	*p*	*n*	*r*	*p*	*n*	*r*	*p*	*n*
Love and relationships	.43	<.001	1832	.42	<.001	539	.43	<.001	1293
Achievement motivation	.61	<.001	1872	.66	<.001	551	.58	<.001	1321
Importance of Relationships	.61	<.001	1872	.58	<.001	551	.63	<.001	1321
Purpose in Life	.68	<.001	1871	.69	<.001	551	.67	<.001	1320
Healthcare	.31	<.001	1869	.39	<.001	550	.27	<.001	1319
Community satisfaction	.43	<.001	1865	.44	<.001	551	.42	<.001	1314
Attitudes towards money	.39	<.001	1831	.41	<.001	546	.38	<.001	1285
Alcohol (frequency x quantity)	.44	<.001	1700	.49	<.001	490	.42	<.001	1210
Conduct problems	.32	<.001	1838	.30	<.001	545	.33	<.001	1293
Emotional problems	.56	<.001	1838	.59	<.001	545	.55	<.001	1293
Hyperactivity	.45	<.001	1838	.49	<.001	545	.43	<.001	1293
Peer problems	.58	<.001	1838	.54	<.001	545	.59	<.001	1293
Prosocial behaviour	.46	<.001	1838	.49	<.001	545	.45	<.001	1293
General anxiety	.57	<.001	1774	.57	<.001	513	.58	<.001	1261
Depression	.56	<.001	1837	.55	<.001	545	.56	<.001	1292
Physical activity	.47	<.001	1846	.51	<.001	545	.45	<.001	1301
Media use	.47	<.001	1841	.57	<.001	545	.43	<.001	1296
Volunteering	.29	<.001	1837	.29	<.001	545	.29	<.001	1292

Note: Scores were corrected for mean age and sex differences (see Methods).

Supplementary Table 12. Measures, test-retest reliabilities and references.

Theme	Variable Name	Scale/item[a]	No. of items	Test-retest[b]	Reference[c]
**Crisis questionnaire**	Multiple items	Items about home environment, life changes, and physical health	41	-	Adapted from the The CoRonavIruS Health Impact Survey (CRISIS; https://github.com/nimh-mbdu/CRISIS)
**Relationships**	Love and relationships	CLAS - Love and Relationships	3	0.71	Lavallee, Hatch, Michalos, and McKinley (2007)
	Verbal peer victimisation	Peer victimisation	4	Kept original scale	Mynard, H., and Joseph (2000)
	Cyber peer victimisation	Peer victimisation	4	Kept original scale	Mynard, H., and Joseph (2000)
	Physical peer victimisation	Peer victimisation	4	Kept original scale	Mynard, H., and Joseph (2000)
**Thoughts and attitudes**	Importance of Relationships	GOALS - self-fulfillment	5	0.73	Pöhlmann and Brunstein (1997)
	Achievement Motivation	GOALS - relationships	4	0.69	Pöhlmann and Brunstein (1997)
	Purpose in Life	Purpose in Life	5	0.82	Crumbaugh and Maholick (1964)
	Healthcare	BSA Democracy and Government (single item about attitudes towards universal healthcare)	1	0.47	Taken from the NatCen Social Research's British Social Attitudes (BSA) Survey (http://natcen.ac.uk/our-research/research/british-social-attitudes/).
	Community satisfaction	CLAS Life Satisfaction Scale - Community.	5	0.5	Lavallee, Hatch, Michalos, and McKinley (2007)

	Attitudes towards money	OECD Financial Literacy: General Money Attitude and Behaviour	1	0.73	Adapted from sections of the OECD instrument for measuring financial literacy: https://www.oecd.org/finance/financial-education/49319977.pdf
**Substance use**	Alcohol frequency	Alcohol use	1	0.71	Adapted from items 1-10 of the AUDIT scale: https://www.drugabuse.gov/sites/default/files/files/AUDIT.pdf
	Alcohol quantity	Alcohol use	1	0.71	Adapted from items 1-10 of the AUDIT scale: https://www.drugabuse.gov/sites/default/files/files/AUDIT.pdf
	Alcohol (frequency x quantity)	Alcohol use	1	0.71	Adapted from items 1-10 of the AUDIT scale: https://www.drugabuse.gov/sites/default/files/files/AUDIT.pdf
	Ever smoked [d]	Smoking (inc. vaping)	1	0.67	Adapted from Heatherton, Kozlowski, Frecker, and Fagerstrom (1991)
	Smoking frequency [d]	Smoking (inc. vaping)	1	0.67	Adapted from Heatherton, Kozlowski, Frecker, and Fagerstrom (1991)
	Ever vaped [d]	Smoking (inc. vaping)	1	0.67	Adapted from Heatherton, Kozlowski, Frecker, and Fagerstrom (1991)
	Vaping frequency [d]	Smoking (inc. vaping)	1	0.67	Adapted from Heatherton, Kozlowski, Frecker, and Fagerstrom (1991)
	Ever used cannabis [d]	Cannabis use	1	Kept original scale	Legleye, Piontek, and Kraus, (2011)
	Cannabis smoking frequency [d]	Cannabis use	1	Kept original scale	Legleye, Piontek, and Kraus, (2011)
**Wellbeing**	Conduct problems	SDQ - Conduct problems	5	0.7	Goodman (1997)
	Emotional problems	SDQ - Emotional problems	5	0.8	Goodman (1997)
	Hyperactivity	SDQ - Hyperactivity	5	0.71	Goodman (1997)

	Peer problems	SDQ - Peer problems	5	0.8	Goodman (1997)
	Prosocial behaviour	SDQ - Prosocial behaviour	5	-	Goodman (1997)
	General anxiety	General Anxiety: the Severity Measure for Generalized Anxiety Disorder	10	Kept the original scale	Craske et al. (2013)
	Depression	Short Mood and Feeling Questionnaire (SMFQ)	8	Kept the original scale	Angold et al. (1995)
	Self-harm	CASE - Self-harm	1	0.56	Adapted from Madge et al. (2008)
	Physical activity	Activity questionnaire	3	Kept the original scale	Questions devised by TEDS researchers.
**Behaviours**	Media use	Media and Technology Usage and Attitudes Scale	4	.73 (video games sub-scale)<br>.82 (social media sub-scale)	Rosen, Whaling, Carrier, Cheever, & Rokkum (2013)
	Volunteering	Volunteering	3	.84	Adapted from page 42 of the ALSPAC questionnaire "It's all about you" at age 20: http://www.bristol.ac.uk/media-library/sites/alspac/migrated/documents/ques-yp20-all-about-you.pdf

[a] More information about the variables and the references can be found in the TEDS data dictionary (**http://www.teds.ac.uk/datadictionary/studies/measures/21yr_measures.htm**).
[b] Test-retest reliability figures are from T1 questionnaire construction.
[c] Note that measures were shortened and adapted from the referenced measures.
[d] Smoking and vaping measures were not included in the present analyses because too few twins endorsed them.

Supplementary Table 13. Multiple regression analysis of phenotypic moderation of sex on T2 responses.

Construct		Unstandardised Coefficients		Standardised Coefficients				95% Confidence Interval for B	
		B	SE	Beta	*t*	*p*	$p_{corrected}$	Lower bound	Upper bound
Love and relationships	Main effect of moderator	.02	.05	.01	0.32	.753	1.00	-.08	.11
	Interaction with T1	.00	.05	.00	0.02	.982	1.00	-.09	.09
Achievement motivation	Main effect of moderator	-.04	.04	-.02	-1.10	.272	1.00	-.12	.04
	Interaction with T1	.03	.04	.02	0.81	.419	1.00	-.04	.10
Importance of Relationships	Main effect of moderator	-.11	.04	-.05	-2.72	.007	1.00	-.19	-.03
	Interaction with T1	-.06	.04	-.04	-1.54	.123	1.00	-.13	.02
Purpose in Life	Main effect of moderator	-.03	.04	-.01	-0.68	.500	1.00	-.10	.05
	Interaction with T1	.00	.04	.00	-0.03	.973	1.00	-.07	.07
Healthcare	Main effect of moderator	.01	.05	.00	0.19	.850	1.00	-.08	.10
	Interaction with T1	.16	.04	.10	3.65	.000	1.00	.08	.25
Community satisfaction	Main effect of moderator	.02	.05	.01	0.42	.672	1.00	-.07	.11
	Interaction with T1	.02	.05	.01	0.34	.732	1.00	-.08	.11
Attitudes towards money	Main effect of moderator	.01	.05	.00	0.15	.884	1.00	-.08	.10
	Interaction with T1	.05	.05	.03	1.16	.248	1.00	-.04	.14
Alcohol (frequency x quantity)	Main effect of moderator	-.01	.05	-.01	-0.24	.814	1.00	-.11	.08
	Interaction with T1	.08	.05	.04	1.56	.120	1.00	-.02	.17
Conduct problems	Main effect of moderator	-.01	.05	-.01	-0.30	.765	1.00	-.11	.08
	Interaction with T1	.00	.05	.00	-0.08	.935	1.00	-.11	.10
Emotional problems	Main effect of moderator	-.04	.04	-.02	-0.88	.377	1.00	-.12	.05
	Interaction with T1	.00	.05	.00	-0.02	.988	1.00	-.09	.09
Hyperactivity	Main effect of moderator	.00	.05	.00	.09	.926	1.00	-.09	.10
	Interaction with T1	.07	.05	.03	1.38	.168	1.00	-.03	.16
Peer problems	Main effect of moderator	-.05	.04	-.02	-1.08	.281	1.00	-.13	.04
	Interaction with T1	-.04	.04	-.02	-1.02	.306	1.00	-.13	.04
	Main effect of moderator	.03	.05	.02	0.73	.463	1.00	-.06	.12

Prosocial behaviour	Interaction with T1	.02	.04	.01	0.35	.724	1.00	-.07	.10
General anxiety	Main effect of moderator	-.03	.04	-.01	-0.65	.514	1.00	-.12	.06
	Interaction with T1	-.05	.05	-.02	-1.03	.304	1.00	-.14	.04
Depression	Main effect of moderator	-.07	.04	-.03	-1.61	.108	1.00	-.15	.02
	Interaction with T1	-.03	.05	-.02	-0.66	.509	1.00	-.12	.06
Physical activity	Main effect of moderator	.04	.05	.02	0.85	.394	1.00	-.05	.13
	Interaction with T1	.05	.04	.03	1.12	.263	1.00	-.04	.14
Media use	Main effect of moderator	.00	.05	.00	0.07	.944	1.00	-.09	.09
	Interaction with T1	.14	.04	.08	3.25	.001	1.00	.06	.23
Volunteering	Main effect of moderator	.01	.05	.01	0.28	.783	1.00	-.08	.11
	Interaction with T1	.02	.05	.01	0.36	.720	1.00	-.08	.11

Note: Scores were corrected for mean age and sex differences (see Methods). Bonferroni correction for multiple comparisons was applied to obtain corrected p values.

Supplementary Table 14. Multiple regression analysis of phenotypic moderation of SES on T2 responses.

**Construct**		Unstandardised Coefficients		Standardised Coefficients				95% Confidence Interval for B	
		B	SE	Beta	*t*	*p*	$p_{corrected}$	Lower bound	Upper bound
Love and relationships	Main effect of moderator	-.02	.02	-.02	-1.00	.319	1.00	-.07	.02
	Interaction with T1	-.02	.02	-.02	-0.84	.399	1.00	-.06	.03
Achievement motivation	Main effect of moderator	.02	.02	.02	1.03	.301	1.00	-.02	.06
	Interaction with T1	.02	.02	.02	1.03	.304	1.00	-.02	.06
Importance of Relationships	Main effect of moderator	.07	.02	.07	3.84	.000	.876	.04	.11
	Interaction with T1	.00	.02	.00	0.04	.967	1.00	-.04	.04
Purpose in Life	Main effect of moderator	.00	.02	.00	-0.18	.856	1.00	-.04	.03
	Interaction with T1	-.03	.02	-.03	-1.75	.080	1.00	-.07	.00
Healthcare	Main effect of moderator	.03	.02	.03	1.46	.145	1.00	-.01	.08
	Interaction with T1	-.06	.02	-.06	-2.77	.006	1.00	-.11	-.02

Community satisfaction	Main effect of moderator	.00	.02	.00	-0.02	.984	1.00	-.04	.04
	Interaction with T1	-.01	.02	-.01	-0.53	.597	1.00	-.05	.03
Attitudes towards money	Main effect of moderator	-.02	.02	-.02	-0.91	.364	1.00	-.07	.02
	Interaction with T1	.01	.02	.01	0.33	.739	1.00	-.04	.05
Alcohol (frequency x quantity)	Main effect of moderator	.00	.02	.00	0.16	.873	1.00	-.04	.05
	Interaction with T1	-.01	.02	-.01	-0.56	.573	1.00	-.06	.03
Conduct problems	Main effect of moderator	-.05	.02	-.06	-2.40	.017	1.00	-.10	-.01
	Interaction with T1	-.02	.02	-.02	-0.93	.352	1.00	-.07	.03
Emotional problems	Main effect of moderator	-.03	.02	-.03	-1.47	.143	1.00	-.07	.01
	Interaction with T1	-.01	.02	-.01	-0.55	.584	1.00	-.05	.03
Hyperactivity	Main effect of moderator	.04	.02	.03	1.58	.114	1.00	-.01	.08
	Interaction with T1	-.01	.02	-.01	-.46	.649	1.00	-.05	.03
Peer problems	Main effect of moderator	-.05	.02	-.05	-2.42	.015	1.00	-.09	-.01
	Interaction with T1	-.03	.02	-.03	-1.36	.174	1.00	-.07	.01
Prosocial behaviour	Main effect of moderator	-.02	.02	-.02	-0.83	.405	1.00	-.06	.02
	Interaction with T1	.03	.02	.03	1.34	.182	1.00	-.01	.07
General anxiety	Main effect of moderator	-.03	.02	-.03	-1.28	.200	1.00	-.07	.01
	Interaction with T1	-.04	.02	-.04	-1.82	.069	1.00	-.08	.00
Depression	Main effect of moderator	-.03	.02	-.03	-1.58	.115	1.00	-.07	.01
	Interaction with T1	-.01	.02	-.01	-0.36	.720	1.00	-.05	.03
Physical activity	Main effect of moderator	.06	.02	.06	2.76	.006	1.00	.02	.10
	Interaction with T1	.02	.02	.02	0.83	.407	1.00	-.03	.06
Media use	Main effect of moderator	-.04	.02	-.04	-1.64	.101	1.00	-.08	.01
	Interaction with T1	.02	.02	.02	0.87	.384	1.00	-.02	.06
Volunteering	Main effect of moderator	-.06	.02	-.06	-2.69	.007	1.00	-.11	-.02
	Interaction with T1	-.03	.02	-.04	-1.42	.156	1.00	-.08	.01

Note: Scores were corrected for mean age and sex differences (see Methods). Bonferroni correction for multiple comparisons was applied to obtain corrected p values.

Supplementary Table 15. Multiple regression analysis of phenotypic moderation of job loss/financial difficulties on T2 responses.

Construct		Unstandardised Coefficients		Standardised Coefficients				95% Confidence Interval for B	
		B	SE	Beta	*t*	*p*	$p_{corrected}$	Lower bound	Upper bound
Love and relationships	Main effect of moderator	-.06	.02	-.06	-2.92	.004	1.00	-.10	-.02
	Interaction with T1	-.01	.02	-.02	-0.73	.465	1.00	-.05	.02
Achievement motivation	Main effect of moderator	.01	.02	.01	0.33	.740	1.00	-.03	.04
	Interaction with T1	-.01	.02	-.01	-0.71	.477	1.00	-.05	.02
Importance of Relationships	Main effect of moderator	.01	.02	.01	0.52	.606	1.00	-.03	.05
	Interaction with T1	-.01	.02	-.01	-0.64	.524	1.00	-.04	.02
Purpose in Life	Main effect of moderator	-.12	.02	-.12	-6.90	.000	.000	-.15	-.09
	Interaction with T1	.04	.02	.04	2.45	.015	1.00	.01	.08
Healthcare	Main effect of moderator	.02	.02	.02	1.01	.312	1.00	-.02	.06
	Interaction with T1	-.03	.02	-.03	-1.33	.185	1.00	-.06	.01
Community satisfaction	Main effect of moderator	-.05	.02	-.05	-2.25	.024	1.00	-.09	-.01
	Interaction with T1	-.02	.02	-.02	-0.77	.442	1.00	-.06	.02
Attitudes towards money	Main effect of moderator	-.07	.02	-.07	-3.03	.002	1.00	-.11	-.02
	Interaction with T1	.01	.02	.01	0.48	.635	1.00	-.03	.05
Alcohol (frequency x quantity)	Main effect of moderator	.03	.02	.03	1.28	.202	1.00	-.02	.08
	Interaction with T1	.00	.02	.00	0.00	.997	1.00	-.04	.04
Conduct problems	Main effect of moderator	.08	.02	.08	3.47	.001	1.00	.03	.12
	Interaction with T1	-.03	.02	-.04	-1.69	.092	1.00	-.07	.01
Emotional problems	Main effect of moderator	.13	.02	.13	6.73	.000	.000	.09	.17
	Interaction with T1	.01	.02	.01	0.36	.722	1.00	-.03	.04
Hyperactivity	Main effect of moderator	.12	.02	.12	5.41	.000	.000	.08	.16
	Interaction with T1	-.03	.02	-.03	-1.31	.189	1.00	-.07	.01
Peer problems	Main effect of moderator	.04	.02	.04	2.01	.045	1.00	.00	.08
	Interaction with T1	.01	.02	.01	0.28	.778	1.00	-.03	.04
	Main effect of moderator	.01	.02	.01	0.27	.785	1.00	-.03	.05

Prosocial behaviour	Interaction with T1	-.08	.02	-.09	-4.20	.000	.187	-.12	-.04
General anxiety	Main effect of moderator	.11	.02	.11	5.42	.000	.000	.07	.16
	Interaction with T1	.00	.02	.00	-0.11	.914	1.00	-.04	.03
Depression	Main effect of moderator	.12	.02	.12	6.09	.000	.000	.08	.16
	Interaction with T1	.02	.02	.02	1.24	.213	1.00	-.01	.06
Physical activity	Main effect of moderator	.03	.02	.03	1.28	.200	1.00	-.01	.07
	Interaction with T1	.02	.02	.02	0.92	.355	1.00	-.02	.05
Media use	Main effect of moderator	.04	.02	.04	1.74	.082	1.00	.00	.08
	Interaction with T1	.00	.02	.00	-0.16	.877	1.00	-.04	.04
Volunteering	Main effect of moderator	.04	.02	.04	1.71	.087	1.00	-.01	.08
	Interaction with T1	-.02	.02	-.02	-0.75	.454	1.00	-.06	.03

Note: Scores were corrected for mean age and sex differences (see Methods). Bonferroni correction for multiple comparisons was applied to obtain corrected p values.

Supplementary Table 16. Multiple regression analysis of phenotypic moderation of garden access on T2 responses.

**Construct**		Unstandardised Coefficients		Standardised Coefficients				95% Confidence Interval for B	
		B	SE	Beta	*t*	*p*	$p_{corrected}$	Lower bound	Upper bound
Love and relationships	Main effect of moderator	-.03	.02	-.03	-1.31	.190	1.00	-.07	.01
	Interaction with T1	-.02	.02	-.02	-0.77	.440	1.00	-.06	.02
Achievement motivation	Main effect of moderator	-.01	.02	-.01	-0.64	.522	1.00	-.05	.02
	Interaction with T1	.03	.02	.03	1.41	.159	1.00	-.01	.06
Importance of Relationships	Main effect of moderator	-.04	.02	-.05	-2.47	.014	1.00	-.08	-.01
	Interaction with T1	.02	.02	.02	1.15	.250	1.00	-.01	.06
Purpose in Life	Main effect of moderator	.03	.02	.03	1.74	.081	1.00	.00	.06
	Interaction with T1	-.03	.02	-.03	-1.72	.085	1.00	-.06	.00
Healthcare	Main effect of moderator	-.04	.02	-.04	-1.80	.071	1.00	-.08	.00
	Interaction with T1	-.03	.03	-.02	-1.06	.288	1.00	-.08	.02

Community satisfaction	Main effect of moderator	.12	.02	.12	5.85	.000	.000	.08	.16
	Interaction with T1	.04	.02	.04	2.14	.033	1.00	.00	.09
Attitudes towards money	Main effect of moderator	.01	.02	.01	0.46	.645	1.00	-.03	.05
	Interaction with T1	.01	.02	.01	0.54	.588	1.00	-.03	.05
Alcohol (frequency x quantity)	Main effect of moderator	-.02	.02	-.02	-0.97	.332	1.00	-.07	.02
	Interaction with T1	.04	.02	.04	1.69	.090	1.00	-.01	.08
Conduct problems	Main effect of moderator	.00	.02	.00	0.16	.870	1.00	-.04	.05
	Interaction with T1	.04	.02	.04	1.64	.102	1.00	-.01	.08
Emotional problems	Main effect of moderator	-.04	.02	-.04	-1.90	.058	1.00	-.07	.00
	Interaction with T1	.02	.02	.02	0.91	.363	1.00	-.02	.05
Hyperactivity	Main effect of moderator	-.05	.02	-.05	-2.33	.020	1.00	-.09	-.01
	Interaction with T1	.02	.02	.02	1.15	.252	1.00	-.02	.06
Peer problems	Main effect of moderator	.00	.02	.00	-0.14	.889	1.00	-.04	.03
	Interaction with T1	.01	.02	.01	0.73	.467	1.00	-.02	.05
Prosocial behaviour	Main effect of moderator	.03	.02	.03	1.47	.141	1.00	-.01	.07
	Interaction with T1	.02	.02	.02	0.87	.383	1.00	-.02	.06
General anxiety	Main effect of moderator	-.04	.02	-.04	-1.81	.071	1.00	-.08	.00
	Interaction with T1	.00	.02	.00	-0.04	.970	1.00	-.04	.04
Depression	Main effect of moderator	-.04	.02	-.04	-2.03	.042	1.00	-.08	.00
	Interaction with T1	.03	.02	.03	1.75	.080	1.00	.00	.07
Physical activity	Main effect of moderator	.00	.02	.00	0.15	.879	1.00	-.04	.04
	Interaction with T1	.00	.02	.00	-0.07	.942	1.00	-.04	.04
Media use	Main effect of moderator	-.04	.02	-.04	-1.99	.046	1.00	-.08	.00
	Interaction with T1	-.03	.02	-.03	-1.51	.131	1.00	-.07	.01
Volunteering	Main effect of moderator	.02	.02	.02	1.10	.271	1.00	-.02	.07
	Interaction with T1	.02	.02	.02	0.73	.464	1.00	-.03	.06

Note: Scores were corrected for mean age and sex differences (see Methods). Bonferroni correction for multiple comparisons was applied to obtain corrected p values.

Supplementary Table 17. Multiple regression analysis of phenotypic moderation of living conditions (number of rooms: people in home during the lockdown) on T2 responses.

Construct		Unstandardised Coefficients		Standardised Coefficients				95% Confidence Interval for B	
		B	SE	Beta	*t*	*p*	$p_{corrected}$	Lower bound	Upper bound
Love and relationships	Main effect of moderator	.00	.02	.00	-0.12	.905	1.00	-.04	.04
	Interaction with T1	.00	.02	.00	-0.07	.947	1.00	-.04	.04
Achievement motivation	Main effect of moderator	-.01	.02	-.01	-0.64	.524	1.00	-.05	.02
	Interaction with T1	-.01	.02	-.01	-0.40	.691	1.00	-.04	.03
Importance of Relationships	Main effect of moderator	.00	.02	.00	0.02	.985	1.00	-.04	.04
	Interaction with T1	.00	.02	.01	0.27	.786	1.00	-.03	.04
Purpose in Life	Main effect of moderator	-.01	.02	-.01	-0.47	.638	1.00	-.04	.03
	Interaction with T1	.00	.02	.00	0.22	.822	1.00	-.03	.04
Healthcare	Main effect of moderator	.01	.02	.01	0.58	.560	1.00	-.03	.05
	Interaction with T1	.01	.02	.02	0.73	.464	1.00	-.02	.05
Community satisfaction	Main effect of moderator	.03	.02	.03	1.45	.148	1.00	-.01	.07
	Interaction with T1	-.04	.02	-.04	-2.02	.043	1.00	-.08	.00
Attitudes towards money	Main effect of moderator	.00	.02	-.01	-0.23	.815	1.00	-.05	.04
	Interaction with T1	.01	.02	.01	0.44	.663	1.00	-.03	.05
Alcohol (frequency x quantity)	Main effect of moderator	-.02	.02	-.02	-0.99	.320	1.00	-.06	.02
	Interaction with T1	-.01	.02	-.01	-0.57	.568	1.00	-.06	.03
Conduct problems	Main effect of moderator	-.05	.02	-.05	-2.35	.019	1.00	-.09	-.01
	Interaction with T1	.00	.02	.00	-0.08	.940	1.00	-.04	.04
Emotional problems	Main effect of moderator	-.02	.02	-.02	-0.83	.408	1.00	-.05	.02
	Interaction with T1	-.04	.02	-.04	-1.98	.048	1.00	-.08	.00
Hyperactivity	Main effect of moderator	-.03	.02	-.03	-1.26	.206	1.00	-.07	.01
	Interaction with T1	-.06	.02	-.06	-2.78	.005	1.00	-.10	-.02
Peer problems	Main effect of moderator	.05	.02	.05	2.44	.015	1.00	.01	.08
	Interaction with T1	.01	.02	.01	0.43	.667	1.00	-.03	.05

Prosocial behaviour	Main effect of moderator	-.02	.02	-.02	-0.89	.372	1.00	-.06	.02
	Interaction with T1	.00	.02	.00	0.18	.858	1.00	-.04	.04
General anxiety	Main effect of moderator	-.05	.02	-.05	-2.74	.006	1.00	-.09	-.02
	Interaction with T1	-.02	.02	-.02	-0.92	.356	1.00	-.05	.02
Depression	Main effect of moderator	.00	.02	.00	0.09	.925	1.00	-.04	.04
	Interaction with T1	-.06	.02	-.05	-2.83	.005	1.00	-.10	-.02
Physical activity	Main effect of moderator	.00	.02	.00	-0.14	.892	1.00	-.04	.04
	Interaction with T1	-.03	.02	-.03	-1.44	.151	1.00	-.07	.01
Media use	Main effect of moderator	.02	.02	.02	1.14	.253	1.00	-.02	.06
	Interaction with T1	.01	.02	.01	0.35	.726	1.00	-.03	.05
Volunteering	Main effect of moderator	-.04	.02	-.04	-1.96	.051	1.00	-.09	.00
	Interaction with T1	-.04	.02	-.04	-2.00	.046	1.00	-.09	.00

Note: Scores were corrected for mean age and sex differences (see Methods). Bonferroni correction for multiple comparisons was applied to obtain corrected p values.

Supplementary Table 18. Multiple regression analysis of phenotypic moderation of having been tested or suspected of having COVID-19 on T2 responses.

**Construct**		Unstandardised Coefficients		Standardised Coefficients				95% Confidence Interval for B	
		B	SE	Beta	*t*	*p*	$p_{corrected}$	Lower bound	Upper bound
Love and relationships	Main effect of moderator	-.03	.02	-.03	-1.45	.147	1.00	-.07	.01
	Interaction with T1	-.02	.02	-.02	-1.04	.298	1.00	-.06	.02
Achievement motivation	Main effect of moderator	.01	.02	.01	0.61	.540	1.00	-.02	.05
	Interaction with T1	.00	.02	.00	-0.05	.960	1.00	-.04	.04
Importance of Relationships	Main effect of moderator	.03	.02	.03	1.53	.126	1.00	-.01	.06
	Interaction with T1	-.03	.02	-.02	-1.28	.200	1.00	-.06	.01
Purpose in Life	Main effect of moderator	.02	.02	.02	1.45	.148	1.00	-.01	.06
	Interaction with T1	-.03	.02	-.02	-1.46	.144	1.00	-.06	.01
Healthcare	Main effect of moderator	-.02	.02	-.02	-1.08	.279	1.00	-.06	.02
	Interaction with T1	.03	.02	.03	1.49	.135	1.00	-.01	.07

Community satisfaction	Main effect of moderator	.01	.02	.01	0.33	.742	1.00	-.03	.05
	Interaction with T1	-.02	.02	-.02	-1.12	.261	1.00	-.06	.02
Attitudes towards money	Main effect of moderator	.00	.02	.00	0.08	.940	1.00	-.04	.04
	Interaction with T1	-.02	.02	-.02	-0.85	.396	1.00	-.06	.02
Alcohol (frequency x quantity)	Main effect of moderator	.02	.02	.02	0.74	.460	1.00	-.03	.06
	Interaction with T1	.04	.02	.04	1.81	.070	1.00	.00	.08
Conduct problems	Main effect of moderator	.06	.02	.06	2.72	.007	1.00	.02	.10
	Interaction with T1	.02	.03	.01	0.65	.514	1.00	-.03	.07
Emotional problems	Main effect of moderator	.06	.02	.06	2.95	.003	1.00	.02	.10
	Interaction with T1	.01	.02	.01	0.44	.657	1.00	-.03	.05
Hyperactivity	Main effect of moderator	.04	.02	.04	2.11	.035	1.00	.00	.09
	Interaction with T1	-.05	.02	-.05	-2.37	.018	1.00	-.09	-.01
Peer problems	Main effect of moderator	.01	.02	.01	0.77	.442	1.00	-.02	.05
	Interaction with T1	.02	.02	.02	1.00	.317	1.00	-.02	.05
Prosocial behaviour	Main effect of moderator	.00	.02	.00	-0.01	.988	1.00	-.04	.04
	Interaction with T1	-.03	.02	-.03	-1.45	.147	1.00	-.07	.01
General anxiety	Main effect of moderator	.06	.02	.06	3.03	.002	1.00	.02	.10
	Interaction with T1	-.04	.02	-.03	-1.79	.073	1.00	-.07	.00
Depression	Main effect of moderator	.03	.02	.03	1.36	.175	1.00	-.01	.06
	Interaction with T1	.03	.02	.03	1.48	.140	1.00	-.01	.07
Physical activity	Main effect of moderator	-.02	.02	-.02	-1.16	.247	1.00	-.06	.02
	Interaction with T1	-.02	.02	-.02	-1.00	.316	1.00	-.06	.02
Media use	Main effect of moderator	.01	.02	.01	0.27	.788	1.00	-.03	.05
	Interaction with T1	-.02	.02	-.02	-1.10	.271	1.00	-.06	.02
Volunteering	Main effect of moderator	.04	.02	.04	1.64	.101	1.00	-.01	.08
	Interaction with T1	.00	.02	.00	0.14	.888	1.00	-.04	.05

Note: Scores were corrected for mean age and sex differences (see Methods). Bonferroni correction for multiple comparisons was applied to obtain corrected p values.

Supplementary Table 19. Multiple regression analysis of phenotypic moderation of COVID-19 symptoms on T2 responses.

Construct		Unstandardised Coefficients		Standardised Coefficients				95% Confidence Interval for B	
		B	SE	Beta	*t*	*p*	$p_{corrected}$	Lower bound	Upper bound
Love and relationships	Main effect of moderator	.04	.02	.05	2.11	.035	1.00	.00	.09
	Interaction with T1	.01	.02	.01	0.26	.795	1.00	-.04	.05
Achievement motivation	Main effect of moderator	-.01	.02	-.01	-0.78	.433	1.00	-.05	.02
	Interaction with T1	.00	.02	.00	0.08	.937	1.00	-.03	.04
Importance of Relationships	Main effect of moderator	-.01	.02	-.01	-0.51	.608	1.00	-.04	.03
	Interaction with T1	.04	.02	.04	2.17	.030	1.00	.00	.08
Purpose in Life	Main effect of moderator	.05	.02	.05	2.83	.005	1.00	.02	.08
	Interaction with T1	.01	.02	.01	0.53	.596	1.00	-.02	.04
Healthcare	Main effect of moderator	.01	.02	.01	0.52	.602	1.00	-.03	.05
	Interaction with T1	-.06	.02	-.06	-2.78	.005	1.00	-.10	-.02
Community satisfaction	Main effect of moderator	.03	.02	.03	1.28	.201	1.00	-.01	.07
	Interaction with T1	.04	.02	.04	2.08	.037	1.00	.00	.08
Attitudes towards money	Main effect of moderator	.02	.02	.02	0.98	.329	1.00	-.02	.06
	Interaction with T1	.03	.02	.03	1.22	.221	1.00	-.02	.07
Alcohol (frequency x quantity)	Main effect of moderator	.00	.02	.00	0.18	.860	1.00	-.04	.05
	Interaction with T1	-.03	.02	-.03	-1.42	.155	1.00	-.08	.01
Conduct problems	Main effect of moderator	-.11	.02	-.11	-5.14	.000	.002	-.15	-.07
	Interaction with T1	.01	.02	.01	0.54	.588	1.00	-.03	.06
Emotional problems	Main effect of moderator	-.11	.02	-.11	-5.85	.000	.000	-.15	-.08
	Interaction with T1	-.01	.02	-.01	-0.42	.672	1.00	-.05	.03
Hyperactivity	Main effect of moderator	-.10	.02	-.10	-4.88	.000	.008	-.14	-.06
	Interaction with T1	.05	.02	.05	2.18	.030	1.00	.00	.09
Peer problems	Main effect of moderator	-.03	.02	-.03	-1.51	.131	1.00	-.07	.01
	Interaction with T1	-.01	.02	-.01	-0.54	.587	1.00	-.05	.03
	Main effect of moderator	.01	.02	.01	0.30	.764	1.00	-.03	.05

Prosocial behaviour	Interaction with T1	.03	.02	.03	1.50	.133	1.00	-.01	.07
General anxiety	Main effect of moderator	-.12	.02	-.12	-6.01	.000	.000	-.16	-.08
	Interaction with T1	.01	.02	.01	0.63	.532	1.00	-.03	.05
Depression	Main effect of moderator	-.08	.02	-.07	-3.86	.000	.798	-.11	-.04
	Interaction with T1	-.05	.02	-.05	-2.46	.014	1.00	-.09	-.01
Physical activity	Main effect of moderator	.05	.02	.05	2.24	.025	1.00	.01	.09
	Interaction with T1	.00	.02	.00	0.07	.945	1.00	-.04	.04
Media use	Main effect of moderator	-.03	.02	-.03	-1.31	.191	1.00	-.07	.01
	Interaction with T1	.00	.02	.00	-0.21	.838	1.00	-.04	.04
Volunteering	Main effect of moderator	.00	.02	.00	-0.13	.896	1.00	-.05	.04
	Interaction with T1	.04	.02	.04	1.65	.099	1.00	-.01	.08

Note: Scores were corrected for mean age and sex differences (see Methods). Bonferroni correction for multiple comparisons was applied to obtain corrected p values.

Supplementary Table 20. Multiple regression analysis of phenotypic moderation of COVID-19 symptoms (2 or more *vs.* none) on T2 responses.

**Construct**		Unstandardised Coefficients		Standardised Coefficients				95% Confidence Interval for B	
		B	SE	Beta	*t*	*p*	$p_{corrected}$	Lower bound	Upper bound
Love and relationships	Main effect of moderator	-.02	.02	-.02	-.84	.402	1.00	-.06	.03
	Interaction with T1	-.01	.02	-.01	-0.36	.719	1.00	-.05	.04
Achievement motivation	Main effect of moderator	.02	.02	.02	0.86	.390	1.00	-.02	.06
	Interaction with T1	.00	.02	.00	0.24	.807	1.00	-.03	.04
Importance of Relationships	Main effect of moderator	.01	.02	.01	0.31	.753	1.00	-.03	.05
	Interaction with T1	-.03	.02	-.03	-1.37	.171	1.00	-.07	.01
Purpose in Life	Main effect of moderator	-.01	.02	-.01	-0.55	.579	1.00	-.05	.03
	Interaction with T1	-.03	.02	-.03	-1.39	.164	1.00	-.07	.01
Healthcare	Main effect of moderator	.00	.02	.00	-0.07	.944	1.00	-.04	.04
	Interaction with T1	.02	.02	.03	1.11	.266	1.00	-.02	.07

Community satisfaction	Main effect of moderator	-.01	.02	-.01	-0.64	.520	1.00	-.06	.03
	Interaction with T1	-.01	.02	-.01	-0.53	.595	1.00	-.06	.03
Attitudes towards money	Main effect of moderator	-.03	.02	-.03	-1.17	.243	1.00	-.07	.02
	Interaction with T1	-.04	.02	-.04	-1.80	.071	1.00	-.09	.00
Alcohol (frequency x quantity)	Main effect of moderator	-.01	.02	-.01	-0.49	.627	1.00	-.06	.04
	Interaction with T1	.04	.03	.04	1.75	.081	1.00	-.01	.09
Conduct problems	Main effect of moderator	.08	.02	.09	3.61	.000	1.00	.04	.13
	Interaction with T1	-.01	.03	-.01	-0.34	.734	1.00	-.06	.04
Emotional problems	Main effect of moderator	.11	.02	.11	4.92	.000	.006	.06	.15
	Interaction with T1	.01	.02	.01	0.50	.617	1.00	-.03	.05
Hyperactivity	Main effect of moderator	.10	.02	.10	4.17	.000	.222	.05	.14
	Interaction with T1	-.05	.02	-.05	-2.28	.023	1.00	-.10	-.01
Peer problems	Main effect of moderator	.04	.02	.04	1.83	.067	1.00	.00	.08
	Interaction with T1	.01	.02	.02	0.76	.447	1.00	-.02	.05
Prosocial behaviour	Main effect of moderator	-.01	.02	-.01	-0.25	.799	1.00	-.05	.04
	Interaction with T1	-.04	.02	-.04	-1.58	.115	1.00	-.08	.01
General anxiety	Main effect of moderator	.13	.02	.13	6.01	.000	.000	.09	.17
	Interaction with T1	-.01	.02	-.02	-0.71	.479	1.00	-.06	.03
Depression	Main effect of moderator	.08	.02	.07	3.45	.001	1.00	.03	.12
	Interaction with T1	.04	.02	.04	1.69	.090	1.00	-.01	.08
Physical activity	Main effect of moderator	-.06	.02	-.06	-2.68	.007	1.00	-.10	-.02
	Interaction with T1	-.01	.02	-.02	-0.69	.493	1.00	-.06	.03
Media use	Main effect of moderator	.02	.02	.02	0.75	.456	1.00	-.03	.06
	Interaction with T1	.00	.02	-.01	-0.23	.820	1.00	-.05	.04
Volunteering	Main effect of moderator	.03	.03	.03	1.12	.263	1.00	-.02	.08
	Interaction with T1	-.04	.03	-.04	-1.43	.154	1.00	-.09	.01

Note: Scores were corrected for mean age and sex differences (see Methods). Bonferroni correction for multiple comparisons was applied to obtain corrected p values.

Supplementary Table 21. Multiple regression analysis of phenotypic moderation of living conditions (twins together *vs.* apart) on T2 responses.

**Construct**		Unstandardised Coefficients		Standardised Coefficients				95% Confidence Interval for B	
		B	SE	Beta	*t*	*p*	$p_{corrected}$	Lower bound	Upper bound
Love and relationships	Main effect of moderator	-.09	.02	-.09	-4.22	.000	.171	-.13	-.05
	Interaction with T1	.01	.02	.01	0.61	.543	1.00	-.03	.06
Achievement motivation	Main effect of moderator	-.01	.02	-.01	-0.48	.632	1.00	-.05	.03
	Interaction with T1	.04	.02	.04	2.38	.018	1.00	.01	.08
Importance of Relationships	Main effect of moderator	-.02	.02	-.02	-1.30	.194	1.00	-.06	.01
	Interaction with T1	.05	.02	.05	2.62	.009	1.00	.01	.08
Purpose in Life	Main effect of moderator	.01	.02	.01	0.78	.437	1.00	-.02	.05
	Interaction with T1	.01	.02	.01	0.41	.680	1.00	-.03	.04
Healthcare	Main effect of moderator	-.01	.02	-.02	-0.68	.494	1.00	-.05	.03
	Interaction with T1	.02	.02	.02	0.85	.398	1.00	-.02	.06
Community satisfaction	Main effect of moderator	.07	.02	.07	3.38	.001	1.00	.03	.11
	Interaction with T1	.12	.02	.12	5.55	.000	.000	.08	.16
Attitudes towards money	Main effect of moderator	-.02	.02	-.02	-0.72	.474	1.00	-.06	.03
	Interaction with T1	.03	.02	.03	1.18	.238	1.00	-.02	.07
Alcohol (frequency x quantity)	Main effect of moderator	-.04	.02	-.04	-1.63	.103	1.00	-.08	.01
	Interaction with T1	.05	.02	.05	2.07	.039	1.00	.00	.09
Conduct problems	Main effect of moderator	.04	.02	.04	1.64	.102	1.00	-.01	.08
	Interaction with T1	.06	.02	.06	2.57	.010	1.00	.01	.10
Emotional problems	Main effect of moderator	-.03	.02	-.03	-1.37	.171	1.00	-.07	.01
	Interaction with T1	.04	.02	.04	2.30	.022	1.00	.01	.08
Hyperactivity	Main effect of moderator	-.01	.02	-.01	-.48	.628	1.00	-.05	.03
	Interaction with T1	.00	.02	.00	-.05	.959	1.00	-.04	.04
Peer problems	Main effect of moderator	-.02	.02	-.02	-1.01	.315	1.00	-.06	.02
	Interaction with T1	.01	.02	.01	0.37	.711	1.00	-.03	.05
	Main effect of moderator	-.02	.02	-.02	-1.14	.256	1.00	-.06	.02

Prosocial behaviour	Interaction with T1	.02	.02	.02	1.18	.238	1.00	-.02	.06
General anxiety	Main effect of moderator	.01	.02	.01	0.40	.691	1.00	-.03	.05
	Interaction with T1	.04	.02	.04	2.22	.026	1.00	.01	.08
Depression	Main effect of moderator	-.03	.02	-.03	-1.36	.174	1.00	-.07	.01
	Interaction with T1	.06	.02	.06	2.89	.004	1.00	.02	.10
Physical activity	Main effect of moderator	.03	.02	.03	1.66	.098	1.00	-.01	.07
	Interaction with T1	-.01	.02	-.01	-0.29	.775	1.00	-.05	.03
Media use	Main effect of moderator	-.04	.02	-.04	-1.74	.081	1.00	-.08	.00
	Interaction with T1	.00	.02	.00	0.11	.911	1.00	-.04	.04
Volunteering	Main effect of moderator	-.01	.02	-.01	-0.24	.812	1.00	-.05	.04
	Interaction with T1	.00	.02	.00	-0.19	.848	1.00	-.05	.04

Note: Scores were corrected for mean age and sex differences (see Methods). Bonferroni correction for multiple comparisons was applied to obtain corrected p values.

Supplementary Table 22. Multiple regression analysis of phenotypic moderation of living conditions (living alone) on T2 responses.

Construct		Unstandardised Coefficients		Standardised Coefficients				95% Confidence Interval for B	
		B	SE	Beta	t	p	$p_{corrected}$	Lower bound	Upper bound
Love and relationships	Main effect of moderator	.11	.02	.11	4.78	.000	.013	.07	.16
	Interaction with T1	.01	.02	.01	0.58	.562	1.00	-.03	.05
Achievement motivation	Main effect of moderator	-.01	.02	-.01	-0.71	.476	1.00	-.05	.02
	Interaction with T1	.01	.02	.01	0.55	.585	1.00	-.02	.04
Importance of Relationships	Main effect of moderator	.04	.02	.04	1.79	.074	1.00	.00	.07
	Interaction with T1	.00	.02	.00	0.22	.827	1.00	-.03	.04
Purpose in Life	Main effect of moderator	.03	.02	.03	1.90	.057	1.00	.00	.07
	Interaction with T1	-.01	.02	-.01	-0.55	.584	1.00	-.05	.03
Healthcare	Main effect of moderator	-.01	.02	-.01	-0.38	.703	1.00	-.05	.03
	Interaction with T1	.01	.02	.01	0.53	.597	1.00	-.02	.04

Community satisfaction	Main effect of moderator	.02	.02	.02	0.75	.453	1.00	-.03	.06
	Interaction with T1	.05	.02	.05	2.32	.021	1.00	.01	.09
Attitudes towards money	Main effect of moderator	.05	.02	.05	2.51	.012	1.00	.01	.10
	Interaction with T1	.01	.02	.01	0.31	.758	1.00	-.04	.05
Alcohol (frequency x quantity)	Main effect of moderator	.01	.02	.01	0.36	.720	1.00	-.04	.05
	Interaction with T1	.00	.02	.00	0.16	.876	1.00	-.04	.04
Conduct problems	Main effect of moderator	.03	.02	.03	1.27	.203	1.00	-.01	.07
	Interaction with T1	.01	.02	.01	0.67	.503	1.00	-.03	.06
Emotional problems	Main effect of moderator	-.04	.02	-.04	-2.17	.030	1.00	-.08	.00
	Interaction with T1	.01	.02	.01	0.71	.479	1.00	-.02	.05
Hyperactivity	Main effect of moderator	-.01	.02	-.01	-.55	.585	1.00	-.05	.03
	Interaction with T1	.05	.02	.05	2.34	.019	1.00	.01	.09
Peer problems	Main effect of moderator	-.13	.02	-.13	-6.87	.000	.000	-.17	-.10
	Interaction with T1	-.01	.02	-.01	-0.33	.742	1.00	-.05	.03
Prosocial behaviour	Main effect of moderator	.06	.02	.06	2.77	.006	1.00	.02	.10
	Interaction with T1	.04	.02	.04	1.84	.066	1.00	.00	.08
General anxiety	Main effect of moderator	-.02	.02	-.02	-0.80	.424	1.00	-.06	.02
	Interaction with T1	.01	.02	.01	0.46	.647	1.00	-.03	.05
Depression	Main effect of moderator	-.10	.02	-.09	-4.89	.000	.008	-.13	-.06
	Interaction with T1	.03	.02	.03	1.31	.189	1.00	-.01	.06
Physical activity	Main effect of moderator	-.01	.02	-.01	-0.27	.787	1.00	-.05	.03
	Interaction with T1	.03	.02	.03	1.38	.167	1.00	-.01	.07
Media use	Main effect of moderator	-.01	.02	-.01	-0.63	.531	1.00	-.05	.03
	Interaction with T1	-.01	.02	-.01	-0.38	.704	1.00	-.05	.03
Volunteering	Main effect of moderator	-.03	.02	-.03	-1.32	.186	1.00	-.07	.01
	Interaction with T1	.02	.02	.02	0.80	.421	1.00	-.03	.06

Note: Scores were corrected for mean age and sex differences (see Methods). Bonferroni correction for multiple comparisons was applied to obtain corrected p values.

Supplementary Table 23. Multiple regression analysis of phenotypic moderation of COVID-19 impact on family health on T2 responses.

**Construct**		Unstandardised Coefficients		Standardised Coefficients				95% Confidence Interval for B	
		B	SE	Beta	*t*	*p*	$p_{corrected}$	Lower bound	Upper bound
Love and relationships	Main effect of moderator	.02	.02	.02	.73	.463	1.00	-.03	.06
	Interaction with T1	-.04	.02	-.04	-1.93	.054	1.00	-.08	.00
Achievement motivation	Main effect of moderator	.01	.02	.01	0.45	.651	1.00	-.03	.04
	Interaction with T1	.02	.02	.02	0.88	.381	1.00	-.02	.06
Importance of Relationships	Main effect of moderator	.03	.02	.03	1.57	.117	1.00	-.01	.06
	Interaction with T1	.00	.02	.00	-0.21	.831	1.00	-.04	.03
Purpose in Life	Main effect of moderator	.00	.02	.00	-0.13	.895	1.00	-.04	.03
	Interaction with T1	-.01	.02	-.01	-0.56	.575	1.00	-.05	.03
Healthcare	Main effect of moderator	.00	.02	.00	0.20	.839	1.00	-.04	.05
	Interaction with T1	-.02	.02	-.02	-0.67	.504	1.00	-.07	.03
Community satisfaction	Main effect of moderator	-.03	.02	-.03	-1.41	.159	1.00	-.07	.01
	Interaction with T1	-.02	.02	-.02	-0.93	.351	1.00	-.06	.02
Attitudes towards money	Main effect of moderator	-.02	.02	-.02	-0.91	.366	1.00	-.06	.02
	Interaction with T1	-.02	.02	-.02	-0.76	.446	1.00	-.06	.03
Alcohol (frequency x quantity)	Main effect of moderator	-.01	.02	-.01	-0.44	.663	1.00	-.05	.03
	Interaction with T1	.01	.02	.01	0.34	.736	1.00	-.04	.05
Conduct problems	Main effect of moderator	.01	.02	.01	0.51	.607	1.00	-.03	.05
	Interaction with T1	.08	.02	.08	3.51	.000	1.00	.04	.13
Emotional problems	Main effect of moderator	.05	.02	.05	2.71	.007	1.00	.01	.09
	Interaction with T1	.02	.02	.02	0.87	.384	1.00	-.02	.06
Hyperactivity	Main effect of moderator	.01	.02	.01	.25	.805	1.00	-.04	.05
	Interaction with T1	-.01	.02	-.01	-.67	.504	1.00	-.05	.03

Peer problems	Main effect of moderator	-.02	.02	-.02	-1.24	.217	1.00	-.06	.01
	Interaction with T1	-.01	.02	-.01	-0.34	.736	1.00	-.05	.03
Prosocial behaviour	Main effect of moderator	.04	.02	.05	2.19	.029	1.00	.00	.09
	Interaction with T1	-.05	.02	-.04	-2.15	.032	1.00	-.09	.00
General anxiety	Main effect of moderator	.03	.02	.03	1.50	.135	1.00	-.01	.07
	Interaction with T1	.00	.02	.00	-0.16	.873	1.00	-.04	.04
Depression	Main effect of moderator	.04	.02	.04	1.82	.069	1.00	.00	.07
	Interaction with T1	.01	.02	.01	0.29	.770	1.00	-.03	.04
Physical activity	Main effect of moderator	.02	.02	.02	1.05	.295	1.00	-.02	.06
	Interaction with T1	.00	.02	.00	0.11	.915	1.00	-.04	.04
Media use	Main effect of moderator	.04	.02	.04	1.83	.067	1.00	.00	.08
	Interaction with T1	.01	.02	.01	0.33	.740	1.00	-.03	.05
Volunteering	Main effect of moderator	.05	.02	.05	2.05	.041	1.00	.00	.09
	Interaction with T1	-.01	.02	-.01	-0.38	.700	1.00	-.05	.03

Note: Scores were corrected for mean age and sex differences (see Methods). Bonferroni correction for multiple comparisons was applied to obtain corrected p values.

Supplementary Table 24. Multiple regression analysis of phenotypic moderation of COVID-19 impact on family financial situation on T2 responses.

**Construct**		Unstandardised Coefficients		Standardised Coefficients				95% Confidence Interval for B	
		B	SE	Beta	*t*	*p*	$p_{corrected}$	Lower bound	Upper bound
Love and relationships	Main effect of moderator	-.02	.02	-.02	-.71	.476	1.00	-.06	.03
	Interaction with T1	.01	.02	.01	0.35	.730	1.00	-.03	.05
Achievement motivation	Main effect of moderator	.00	.02	.00	-0.04	.968	1.00	-.04	.04
	Interaction with T1	.00	.02	.00	0.01	.994	1.00	-.04	.04
Importance of Relationships	Main effect of moderator	.02	.02	.02	1.05	.293	1.00	-.02	.05
	Interaction with T1	.01	.02	.01	0.47	.638	1.00	-.03	.04
Purpose in Life	Main effect of moderator	-.04	.02	-.04	-2.31	.021	1.00	-.07	-.01
	Interaction with T1	-.01	.02	-.01	-0.42	.674	1.00	-.04	.03

Healthcare	Main effect of moderator	-.04	.02	-.04	-1.99	.047	1.00	-.08	.00
	Interaction with T1	.03	.02	.03	1.17	.241	1.00	-.02	.07
Community satisfaction	Main effect of moderator	-.03	.02	-.03	-1.33	.183	1.00	-.07	.01
	Interaction with T1	-.05	.02	-.05	-2.27	.023	1.00	-.08	-.01
Attitudes towards money	Main effect of moderator	-.04	.02	-.04	-1.93	.053	1.00	-.08	.00
	Interaction with T1	.01	.02	.01	0.65	.517	1.00	-.03	.05
Alcohol (frequency x quantity)	Main effect of moderator	-.01	.02	-.01	-0.38	.704	1.00	-.05	.03
	Interaction with T1	.02	.02	.02	0.86	.390	1.00	-.02	.06
Conduct problems	Main effect of moderator	-.01	.02	-.01	-0.57	.570	1.00	-.05	.03
	Interaction with T1	-.06	.02	-.06	-2.59	.010	1.00	-.10	-.01
Emotional problems	Main effect of moderator	.05	.02	.05	2.67	.008	1.00	.01	.09
	Interaction with T1	-.01	.02	-.01	-0.56	.572	1.00	-.05	.03
Hyperactivity	Main effect of moderator	.04	.02	.04	2.07	.039	1.00	.00	.09
	Interaction with T1	-.02	.02	-.02	-.98	.327	1.00	-.06	.02
Peer problems	Main effect of moderator	.00	.02	.00	-0.08	.940	1.00	-.04	.04
	Interaction with T1	-.05	.02	-.05	-2.37	.018	1.00	-.08	-.01
Prosocial behaviour	Main effect of moderator	.00	.02	.00	-0.18	.857	1.00	-.04	.04
	Interaction with T1	.02	.02	.02	1.01	.314	1.00	-.02	.06
General anxiety	Main effect of moderator	.06	.02	.06	2.88	.004	1.00	.02	.10
	Interaction with T1	-.01	.02	-.01	-0.62	.533	1.00	-.05	.03
Depression	Main effect of moderator	.04	.02	.04	1.95	.051	1.00	.00	.08
	Interaction with T1	.00	.02	.00	-0.23	.816	1.00	-.04	.03
Physical activity	Main effect of moderator	.03	.02	.03	1.36	.174	1.00	-.01	.07
	Interaction with T1	.00	.02	.00	0.04	.965	1.00	-.04	.04
Media use	Main effect of moderator	.08	.02	.08	3.91	.000	.657	.04	.12
	Interaction with T1	.02	.02	.02	1.06	.289	1.00	-.02	.06
Volunteering	Main effect of moderator	.02	.02	.02	0.88	.381	1.00	-.02	.06
	Interaction with T1	-.02	.02	-.02	-0.90	.366	1.00	-.06	.02

Note: Scores were corrected for mean age and sex differences (see Methods). Bonferroni correction for multiple comparisons was applied to obtain corrected p values.

Supplementary Table 25. Multiple regression analysis of phenotypic moderation of COVID-19 impact on family health and financial situation on T2 responses.

**Construct**		Unstandardised Coefficients		Standardised Coefficients				95% Confidence Interval for B	
		B	SE	Beta	*t*	*p*	$p_{corrected}$	Lower bound	Upper bound
Love and relationships	Main effect of moderator	.00	.02	.00	.14	.893	1.00	-.04	.04
	Interaction with T1	-.02	.02	-.03	-1.19	.233	1.00	-.06	.02
Achievement motivation	Main effect of moderator	.01	.02	.01	0.34	.732	1.00	-.03	.04
	Interaction with T1	.01	.02	.01	0.67	.502	1.00	-.02	.05
Importance of Relationships	Main effect of moderator	.03	.02	.03	1.74	.082	1.00	.00	.07
	Interaction with T1	.00	.02	.00	0.15	.883	1.00	-.04	.04
Purpose in Life	Main effect of moderator	-.02	.02	-.02	-1.44	.150	1.00	-.06	.01
	Interaction with T1	-.01	.02	-.01	-0.54	.589	1.00	-.04	.03
Healthcare	Main effect of moderator	-.02	.02	-.02	-1.05	.296	1.00	-.06	.02
	Interaction with T1	.01	.02	.01	0.24	.813	1.00	-.04	.05
Community satisfaction	Main effect of moderator	-.04	.02	-.04	-1.74	.082	1.00	-.08	.00
	Interaction with T1	-.04	.02	-.04	-2.04	.042	1.00	-.08	.00
Attitudes towards money	Main effect of moderator	-.04	.02	-.04	-1.73	.083	1.00	-.08	.00
	Interaction with T1	-.01	.02	-.01	-0.27	.786	1.00	-.05	.04
Alcohol (frequency x quantity)	Main effect of moderator	-.01	.02	-.01	-0.56	.575	1.00	-.06	.03
	Interaction with T1	.02	.02	.02	0.75	.455	1.00	-.03	.06
Conduct problems	Main effect of moderator	.00	.02	.00	-0.09	.929	1.00	-.04	.04
	Interaction with T1	.02	.02	.02	1.03	.304	1.00	-.02	.07
Emotional problems	Main effect of moderator	.07	.02	.07	3.56	.000	1.00	.03	.11
	Interaction with T1	.01	.02	.01	0.40	.687	1.00	-.03	.05
Hyperactivity	Main effect of moderator	.03	.02	.03	1.34	.180	1.00	-.01	.07
	Interaction with T1	-.02	.02	-.02	-.96	.339	1.00	-.06	.02
Peer problems	Main effect of moderator	-.02	.02	-.02	-1.08	.282	1.00	-.06	.02

	Interaction with T1	-.03	.02	-.03	-1.63	.104	1.00	-.07	.01
Prosocial behaviour	Main effect of moderator	.03	.02	.03	1.38	.167	1.00	-.01	.07
	Interaction with T1	-.02	.02	-.02	-0.87	.386	1.00	-.06	.02
General anxiety	Main effect of moderator	.05	.02	.05	2.76	.006	1.00	.02	.09
	Interaction with T1	-.01	.02	-.01	-0.46	.649	1.00	-.05	.03
Depression	Main effect of moderator	.05	.02	.05	2.48	.013	1.00	.01	.09
	Interaction with T1	.00	.02	.00	0.16	.871	1.00	-.04	.04
Physical activity	Main effect of moderator	.03	.02	.03	1.56	.118	1.00	-.01	.07
	Interaction with T1	.00	.02	.00	0.14	.891	1.00	-.04	.04
Media use	Main effect of moderator	.07	.02	.07	3.61	.000	1.00	.03	.11
	Interaction with T1	.02	.02	.02	0.84	.403	1.00	-.02	.06
Volunteering	Main effect of moderator	.05	.02	.05	2.03	.042	1.00	.00	.09
	Interaction with T1	-.02	.02	-.02	-0.78	.437	1.00	-.06	.02

Note: Scores were corrected for mean age and sex differences (see Methods). Bonferroni correction for multiple comparisons was applied to obtain corrected p values.

Supplementary Table 26. Multiple regression analysis of phenotypic moderation of family death due to COVID-19 on T2 responses.

**Construct**		Unstandardised Coefficients		Standardised Coefficients				95% Confidence Interval for B	
		B	SE	Beta	*t*	*p*	$p_{corrected}$	Lower bound	Upper bound
Love and relationships	Main effect of moderator	.04	.02	.04	1.67	.095	1.00	-.01	.08
	Interaction with T1	-.04	.02	-.04	-1.83	.068	1.00	-.08	.00
Achievement motivation	Main effect of moderator	.03	.02	.03	1.85	.064	1.00	.00	.07
	Interaction with T1	-.02	.02	-.02	-1.02	.310	1.00	-.07	.02
Importance of Relationships	Main effect of moderator	.07	.02	.07	3.42	.001	1.00	.03	.11
	Interaction with T1	-.04	.03	-.03	-1.27	.204	1.00	-.10	.02
Purpose in Life	Main effect of moderator	.00	.02	.00	0.25	.800	1.00	-.03	.04
	Interaction with T1	.01	.02	.01	0.39	.697	1.00	-.03	.05
Healthcare	Main effect of moderator	.01	.03	.01	0.24	.813	1.00	-.04	.06

	Interaction with T1	.00	.04	.00	0.05	.956	1.00	-.08	.08
Community satisfaction	Main effect of moderator	-.02	.02	-.02	-0.86	.392	1.00	-.06	.02
	Interaction with T1	-.03	.02	-.03	-1.43	.152	1.00	-.07	.01
Attitudes towards money	Main effect of moderator	.00	.02	.00	0.22	.822	1.00	-.04	.05
	Interaction with T1	.01	.02	.01	0.48	.630	1.00	-.03	.05
Alcohol (frequency x quantity)	Main effect of moderator	-.01	.02	-.01	-0.38	.704	1.00	-.05	.04
	Interaction with T1	-.01	.02	-.01	-0.64	.525	1.00	-.06	.03
Conduct problems	Main effect of moderator	.01	.02	.01	0.47	.638	1.00	-.03	.05
	Interaction with T1	.08	.02	.08	3.75	.000	1.00	.04	.12
Emotional problems	Main effect of moderator	.05	.02	.05	2.30	.021	1.00	.01	.09
	Interaction with T1	.02	.02	.02	1.00	.317	1.00	-.02	.06
Hyperactivity	Main effect of moderator	.02	.02	.02	.77	.439	1.00	-.03	.06
	Interaction with T1	-.02	.02	-.01	-.66	.508	1.00	-.06	.03
Peer problems	Main effect of moderator	-.02	.02	-.02	-0.82	.414	1.00	-.05	.02
	Interaction with T1	.02	.02	.02	1.05	.294	1.00	-.02	.05
Prosocial behaviour	Main effect of moderator	.04	.02	.04	1.84	.065	1.00	.00	.09
	Interaction with T1	-.02	.03	-.02	-0.80	.423	1.00	-.08	.03
General anxiety	Main effect of moderator	.04	.02	.04	1.80	.072	1.00	.00	.08
	Interaction with T1	.01	.02	.01	0.35	.728	1.00	-.03	.04
Depression	Main effect of moderator	.02	.02	.02	1.20	.229	1.00	-.02	.06
	Interaction with T1	.00	.02	.00	-0.07	.944	1.00	-.03	.03
Physical activity	Main effect of moderator	.00	.02	.00	-0.17	.866	1.00	-.04	.04
	Interaction with T1	.02	.02	.02	0.79	.433	1.00	-.02	.05
Media use	Main effect of moderator	.02	.02	.02	1.00	.316	1.00	-.02	.07
	Interaction with T1	-.02	.02	-.02	-1.08	.279	1.00	-.07	.02
Volunteering	Main effect of moderator	.00	.02	.00	0.16	.876	1.00	-.04	.05
	Interaction with T1	-.02	.02	-.02	-0.87	.382	1.00	-.06	.02

Note: Scores were corrected for mean age and sex differences (see Methods). Bonferroni correction for multiple comparisons was applied to obtain corrected p values.

Supplementary Table 27. Multiple regression analysis of phenotypic moderation of worries of being infected on T2 responses.

Construct		Unstandardised Coefficients		Standardised Coefficients				95% Confidence Interval for B	
		B	SE	Beta	*t*	*p*	$p_{corrected}$	Lower bound	Upper bound
Love and relationships	Main effect of moderator	.01	.02	.02	.69	.491	1.00	-.03	.06
	Interaction with T1	.00	.02	.00	0.15	.884	1.00	-.04	.04
Achievement motivation	Main effect of moderator	.05	.02	.05	2.50	.012	1.00	.01	.08
	Interaction with T1	-.02	.02	-.02	-1.14	.255	1.00	-.06	.01
Importance of Relationships	Main effect of moderator	.06	.02	.06	3.14	.002	1.00	.02	.09
	Interaction with T1	.00	.02	.00	-0.25	.801	1.00	-.04	.03
Purpose in Life	Main effect of moderator	.00	.02	.00	-0.29	.772	1.00	-.04	.03
	Interaction with T1	-.05	.02	-.05	-2.98	.003	1.00	-.08	-.02
Healthcare	Main effect of moderator	.04	.02	.04	1.90	.058	1.00	.00	.08
	Interaction with T1	-.04	.02	-.04	-1.75	.080	1.00	-.08	.00
Community satisfaction	Main effect of moderator	-.05	.02	-.05	-2.45	.015	1.00	-.09	-.01
	Interaction with T1	-.02	.02	-.02	-1.05	.292	1.00	-.06	.02
Attitudes towards money	Main effect of moderator	-.04	.02	-.04	-2.00	.046	1.00	-.08	.00
	Interaction with T1	.01	.02	.01	0.56	.572	1.00	-.03	.05
Alcohol (frequency x quantity)	Main effect of moderator	-.04	.02	-.04	-1.79	.074	1.00	-.08	.00
	Interaction with T1	.01	.02	.01	0.59	.555	1.00	-.03	.06
Conduct problems	Main effect of moderator	.07	.02	.07	3.16	.002	1.00	.03	.11
	Interaction with T1	.04	.02	.04	1.66	.098	1.00	-.01	.08
Emotional problems	Main effect of moderator	.24	.02	.24	12.50	.000	.000	.20	.28
	Interaction with T1	.03	.02	.03	1.76	.079	1.00	.00	.07
Hyperactivity	Main effect of moderator	.09	.02	.09	4.13	.000	.262	.05	.13
	Interaction with T1	.02	.02	.02	.91	.365	1.00	-.02	.06
Peer problems	Main effect of moderator	.03	.02	.03	1.67	.095	1.00	-.01	.07
	Interaction with T1	.03	.02	.03	1.60	.109	1.00	-.01	.07

Prosocial behaviour	Main effect of moderator	.04	.02	.04	1.88	.060	1.00	.00	.08
	Interaction with T1	.00	.02	.00	0.15	.882	1.00	-.04	.04
General anxiety	Main effect of moderator	.23	.02	.23	11.86	.000	.000	.19	.27
	Interaction with T1	.03	.02	.04	2.01	.045	1.00	.00	.07
Depression	Main effect of moderator	.12	.02	.12	6.26	.000	.000	.08	.16
	Interaction with T1	.03	.02	.03	1.81	.071	1.00	.00	.07
Physical activity	Main effect of moderator	-.06	.02	-.06	-3.08	.002	1.00	-.10	-.02
	Interaction with T1	-.05	.02	-.05	-2.55	.011	1.00	-.09	-.01
Media use	Main effect of moderator	.02	.02	.02	0.87	.387	1.00	-.02	.06
	Interaction with T1	.01	.02	.01	0.72	.473	1.00	-.03	.05
Volunteering	Main effect of moderator	.01	.02	.01	0.58	.559	1.00	-.03	.06
	Interaction with T1	-.03	.02	-.03	-1.44	.149	1.00	-.07	.01

Note: Scores were corrected for mean age and sex differences (see Methods). Bonferroni correction for multiple comparisons was applied to obtain corrected p values.

Supplementary Table 28. Multiple regression analysis of phenotypic moderation of worries of family or friends being infected on T2 responses.

**Construct**		Unstandardised Coefficients		Standardised Coefficients				95% Confidence Interval for B	
		B	SE	Beta	*t*	*p*	$p_{corrected}$	Lower bound	Upper bound
Love and relationships	Main effect of moderator	.03	.02	.03	1.51	.132	1.00	-.01	.07
	Interaction with T1	.00	.02	.00	0.09	.933	1.00	-.04	.04
Achievement motivation	Main effect of moderator	.04	.02	.04	2.11	.035	1.00	.00	.07
	Interaction with T1	-.02	.02	-.03	-1.39	.164	1.00	-.06	.01
Importance of Relationships	Main effect of moderator	.06	.02	.06	3.03	.003	1.00	.02	.09
	Interaction with T1	-.01	.02	-.01	-0.44	.658	1.00	-.04	.03
Purpose in Life	Main effect of moderator	-.02	.02	-.02	-1.40	.161	1.00	-.06	.01
	Interaction with T1	-.03	.02	-.03	-1.60	.110	1.00	-.06	.01
Healthcare	Main effect of moderator	.06	.02	.06	2.74	.006	1.00	.02	.10
	Interaction with T1	-.07	.02	-.07	-3.35	.001	1.00	-.11	-.03

Community satisfaction	Main effect of moderator	-.04	.02	-.04	-1.99	.047	1.00	-.08	.00
	Interaction with T1	-.01	.02	-.01	-0.52	.601	1.00	-.05	.03
Attitudes towards money	Main effect of moderator	-.06	.02	-.06	-2.75	.006	1.00	-.10	-.02
	Interaction with T1	-.02	.02	-.02	-0.81	.420	1.00	-.06	.02
Alcohol (frequency x quantity)	Main effect of moderator	-.01	.02	-.01	-0.48	.633	1.00	-.05	.03
	Interaction with T1	.00	.02	.00	-0.15	.884	1.00	-.05	.04
Conduct problems	Main effect of moderator	.07	.02	.07	3.06	.002	1.00	.02	.11
	Interaction with T1	.01	.02	.01	0.42	.672	1.00	-.03	.05
Emotional problems	Main effect of moderator	.23	.02	.23	12.13	.000	.000	.19	.27
	Interaction with T1	.03	.02	.03	1.64	.102	1.00	-.01	.07
Hyperactivity	Main effect of moderator	.09	.02	.09	4.30	.000	.124	.05	.13
	Interaction with T1	-.02	.02	-.02	-.99	.322	1.00	-.06	.02
Peer problems	Main effect of moderator	.01	.02	.01	0.65	.515	1.00	-.02	.05
	Interaction with T1	.02	.02	.02	0.86	.387	1.00	-.02	.05
Prosocial behaviour	Main effect of moderator	.08	.02	.08	4.01	.000	.436	.04	.12
	Interaction with T1	.02	.02	.02	0.94	.348	1.00	-.02	.06
General anxiety	Main effect of moderator	.21	.02	.20	10.54	.000	.000	.17	.25
	Interaction with T1	.03	.02	.03	1.72	.085	1.00	.00	.07
Depression	Main effect of moderator	.12	.02	.12	6.05	.000	.000	.08	.16
	Interaction with T1	.00	.02	.00	0.22	.824	1.00	-.03	.04
Physical activity	Main effect of moderator	.00	.02	.00	-0.15	.880	1.00	-.04	.04
	Interaction with T1	-.01	.02	-.01	-0.26	.795	1.00	-.04	.03
Media use	Main effect of moderator	.02	.02	.02	0.96	.339	1.00	-.02	.06
	Interaction with T1	-.01	.02	-.01	-0.64	.519	1.00	-.05	.03
Volunteering	Main effect of moderator	.06	.02	.06	2.83	.005	1.00	.02	.10
	Interaction with T1	.00	.02	.00	-0.21	.832	1.00	-.05	.04

Note: Scores were corrected for mean age and sex differences (see Methods). Bonferroni correction for multiple comparisons was applied to obtain corrected p values.

Supplementary Table 29. Multiple regression analysis of phenotypic moderation of worries of physical health being influenced by COVID-19 on T2 responses.

Construct		Unstandardised Coefficients		Standardised Coefficients				95% Confidence Interval for B	
		B	SE	Beta	*t*	*p*	$p_{corrected}$	Lower bound	Upper bound
Love and relationships	Main effect of moderator	.00	.02	.00	.06	.953	1.00	-.04	.04
	Interaction with T1	-.03	.02	-.04	-1.63	.102	1.00	-.07	.01
Achievement motivation	Main effect of moderator	.05	.02	.05	2.72	.007	1.00	.01	.08
	Interaction with T1	-.01	.02	-.02	-0.83	.405	1.00	-.05	.02
Importance of Relationships	Main effect of moderator	.04	.02	.04	2.25	.025	1.00	.01	.08
	Interaction with T1	-.01	.02	-.01	-0.78	.437	1.00	-.05	.02
Purpose in Life	Main effect of moderator	-.03	.02	-.03	-1.92	.055	1.00	-.07	.00
	Interaction with T1	-.04	.02	-.04	-2.42	.015	1.00	-.07	-.01
Healthcare	Main effect of moderator	.03	.02	.03	1.50	.134	1.00	-.01	.07
	Interaction with T1	-.03	.02	-.04	-1.62	.105	1.00	-.07	.01
Community satisfaction	Main effect of moderator	-.07	.02	-.07	-3.16	.002	1.00	-.11	-.03
	Interaction with T1	-.01	.02	-.01	-0.63	.527	1.00	-.05	.03
Attitudes towards money	Main effect of moderator	-.05	.02	-.05	-2.43	.015	1.00	-.09	-.01
	Interaction with T1	-.01	.02	-.01	-0.56	.575	1.00	-.05	.03
Alcohol (frequency x quantity)	Main effect of moderator	-.02	.02	-.02	-0.87	.384	1.00	-.06	.02
	Interaction with T1	-.01	.02	-.01	-0.23	.815	1.00	-.05	.04
Conduct problems	Main effect of moderator	.08	.02	.08	3.85	.000	.815	.04	.12
	Interaction with T1	.05	.02	.05	2.19	.028	1.00	.00	.09
Emotional problems	Main effect of moderator	.22	.02	.22	11.34	.000	.000	.18	.25
	Interaction with T1	.01	.02	.01	0.52	.604	1.00	-.03	.04
Hyperactivity	Main effect of moderator	.14	.02	.13	6.47	.000	.000	.09	.18
	Interaction with T1	-.01	.02	-.01	-.69	.490	1.00	-.05	.03
Peer problems	Main effect of moderator	.04	.02	.04	2.18	.030	1.00	.00	.08
	Interaction with T1	.02	.02	.02	1.00	.318	1.00	-.02	.05

Prosocial behaviour	Main effect of moderator	.06	.02	.06	2.98	.003	1.00	.02	.10
	Interaction with T1	-.01	.02	-.01	-0.54	.588	1.00	-.05	.03
General anxiety	Main effect of moderator	.20	.02	.19	10.11	.000	.000	.16	.23
	Interaction with T1	.01	.02	.01	0.44	.663	1.00	-.03	.04
Depression	Main effect of moderator	.15	.02	.15	7.67	.000	.000	.11	.19
	Interaction with T1	.03	.02	.03	1.48	.140	1.00	-.01	.06
Physical activity	Main effect of moderator	-.07	.02	-.07	-3.60	.000	1.00	-.11	-.03
	Interaction with T1	-.02	.02	-.02	-1.05	.293	1.00	-.06	.02
Media use	Main effect of moderator	.06	.02	.06	3.13	.002	1.00	.02	.10
	Interaction with T1	-.01	.02	-.01	-0.31	.756	1.00	-.04	.03
Volunteering	Main effect of moderator	.02	.02	.02	0.72	.469	1.00	-.03	.06
	Interaction with T1	-.04	.02	-.04	-1.79	.074	1.00	-.08	.00

Note: Scores were corrected for mean age and sex differences (see Methods). Bonferroni correction for multiple comparisons was applied to obtain corrected p values.

Supplementary Table 30. Multiple regression analysis of phenotypic moderation of worries of mental/emotional health being influenced by COVID-19 on T2 responses.

**Construct**		Unstandardised Coefficients		Standardised Coefficients				95% Confidence Interval for B	
		B	SE	Beta	*t*	*p*	$p_{\text{corrected}}$	Lower bound	Upper bound
Love and relationships	Main effect of moderator	-.08	.02	-.08	-3.90	.000	.694	-.12	-.04
	Interaction with T1	-.04	.02	-.04	-1.98	.047	1.00	-.08	.00
Achievement motivation	Main effect of moderator	.04	.02	.05	2.49	.013	1.00	.01	.08
	Interaction with T1	.00	.02	.00	-0.27	.790	1.00	-.04	.03
Importance of Relationships	Main effect of moderator	.09	.02	.10	5.24	.000	.001	.06	.13
	Interaction with T1	-.05	.02	-.05	-2.77	.006	1.00	-.08	-.01
Purpose in Life	Main effect of moderator	-.13	.02	-.13	-7.39	.000	.000	-.16	-.09
	Interaction with T1	-.02	.02	-.03	-1.54	.123	1.00	-.06	.01
Healthcare	Main effect of moderator	.04	.02	.04	1.96	.050	1.00	.00	.08

	Interaction with T1	-.01	.02	-.01	-0.46	.646	1.00	-.05	.03
Community satisfaction	Main effect of moderator	-.11	.02	-.11	-5.15	.000	.002	-.15	-.07
	Interaction with T1	-.01	.02	-.01	-0.32	.747	1.00	-.05	.03
Attitudes towards money	Main effect of moderator	-.05	.02	-.05	-2.37	.018	1.00	-.09	-.01
	Interaction with T1	-.04	.02	-.04	-1.84	.066	1.00	-.08	.00
Alcohol (frequency x quantity)	Main effect of moderator	.00	.02	.00	0.12	.907	1.00	-.04	.05
	Interaction with T1	-.01	.02	-.01	-0.38	.706	1.00	-.05	.04
Conduct problems	Main effect of moderator	.13	.02	.13	6.07	.000	.000	.09	.17
	Interaction with T1	.05	.02	.06	2.58	.010	1.00	.01	.09
Emotional problems	Main effect of moderator	.41	.02	.41	22.76	.000	.000	.38	.45
	Interaction with T1	.00	.02	.00	0.11	.910	1.00	-.03	.03
Hyperactivity	Main effect of moderator	.29	.02	.29	14.39	.000	.000	.25	.33
	Interaction with T1	-.04	.02	-.04	-2.05	.041	1.00	-.08	.00
Peer problems	Main effect of moderator	.10	.02	.10	5.41	.000	.000	.06	.14
	Interaction with T1	-.01	.02	-.01	-0.61	.543	1.00	-.05	.02
Prosocial behaviour	Main effect of moderator	.01	.02	.01	0.67	.501	1.00	-.03	.05
	Interaction with T1	-.02	.02	-.02	-0.97	.332	1.00	-.06	.02
General anxiety	Main effect of moderator	.40	.02	.40	21.82	.000	.000	.37	.44
	Interaction with T1	.05	.02	.05	2.98	.003	1.00	.02	.08
Depression	Main effect of moderator	.36	.02	.36	19.69	.000	.000	.33	.40
	Interaction with T1	.00	.02	.00	0.25	.803	1.00	-.03	.04
Physical activity	Main effect of moderator	-.05	.02	-.06	-2.72	.007	1.00	-.09	-.02
	Interaction with T1	-.03	.02	-.03	-1.46	.145	1.00	-.07	.01
Media use	Main effect of moderator	.08	.02	.08	4.03	.000	.391	.04	.12
	Interaction with T1	-.02	.02	-.02	-0.75	.455	1.00	-.05	.02
Volunteering	Main effect of moderator	.02	.02	.02	0.83	.404	1.00	-.02	.06
	Interaction with T1	-.05	.02	-.05	-2.41	.016	1.00	-.09	-.01

Note: Scores were corrected for mean age and sex differences (see Methods). Bonferroni correction for multiple comparisons was applied to obtain corrected p values.

Supplementary Table 31. Multiple regression analysis of phenotypic moderation of change in sleep habits on T2 responses.

Construct		Unstandardised Coefficients		Standardised Coefficients				95% Confidence Interval for B	
		B	SE	Beta	*t*	*p*	$p_{corrected}$	Lower bound	Upper bound
Love and relationships	Main effect of moderator	.05	.02	.05	2.17	.030	1.00	.00	.09
	Interaction with T1	-.02	.02	-.02	-1.08	.281	1.00	-.06	.02
Achievement motivation	Main effect of moderator	.01	.02	.01	0.32	.748	1.00	-.03	.04
	Interaction with T1	.00	.02	.00	-0.24	.808	1.00	-.04	.03
Importance of Relationships	Main effect of moderator	-.02	.02	-.02	-1.21	.225	1.00	-.06	.01
	Interaction with T1	.00	.02	.00	0.14	.892	1.00	-.03	.04
Purpose in Life	Main effect of moderator	.01	.02	.01	0.30	.767	1.00	-.03	.04
	Interaction with T1	-.01	.02	-.01	-0.57	.571	1.00	-.04	.02
Healthcare	Main effect of moderator	.01	.02	.01	0.35	.729	1.00	-.03	.05
	Interaction with T1	-.04	.02	-.05	-2.20	.028	1.00	-.07	.00
Community satisfaction	Main effect of moderator	.02	.02	.02	0.84	.398	1.00	-.02	.06
	Interaction with T1	.02	.02	.02	1.09	.278	1.00	-.02	.06
Attitudes towards money	Main effect of moderator	.01	.02	.01	0.34	.733	1.00	-.03	.05
	Interaction with T1	.00	.02	.00	-0.14	.889	1.00	-.04	.04
Alcohol (frequency x quantity)	Main effect of moderator	.02	.02	.02	0.88	.380	1.00	-.02	.06
	Interaction with T1	-.01	.02	-.01	-0.35	.726	1.00	-.05	.04
Conduct problems	Main effect of moderator	-.07	.02	-.08	-3.55	.000	1.00	-.12	-.03
	Interaction with T1	-.06	.02	-.07	-3.15	.002	1.00	-.10	-.02
Emotional problems	Main effect of moderator	-.10	.02	-.10	-5.16	.000	.002	-.14	-.06
	Interaction with T1	-.02	.02	-.02	-0.94	.346	1.00	-.05	.02
Hyperactivity	Main effect of moderator	-.04	.02	-.04	-1.95	.051	1.00	-.08	.00
	Interaction with T1	-.02	.02	-.03	-1.25	.213	1.00	-.06	.01
Peer problems	Main effect of moderator	-.04	.02	-.04	-2.21	.027	1.00	-.08	.00
	Interaction with T1	-.04	.02	-.04	-2.31	.021	1.00	-.07	-.01
	Main effect of moderator	-.03	.02	-.03	-1.27	.206	1.00	-.07	.01

Prosocial behaviour	Interaction with T1	.00	.02	.00	0.21	.833	1.00	-.04	.04
General anxiety	Main effect of moderator	-.13	.02	-.13	-6.80	.000	.000	-.17	-.10
	Interaction with T1	-.04	.02	-.04	-1.95	.052	1.00	-.07	.00
Depression	Main effect of moderator	-.09	.02	-.09	-4.35	.000	.096	-.12	-.05
	Interaction with T1	-.02	.02	-.02	-0.84	.402	1.00	-.05	.02
Physical activity	Main effect of moderator	.00	.02	.00	-0.23	.821	1.00	-.04	.04
	Interaction with T1	.01	.02	.01	0.55	.580	1.00	-.03	.05
Media use	Main effect of moderator	.01	.02	.01	0.70	.483	1.00	-.03	.05
	Interaction with T1	.01	.02	.01	0.56	.577	1.00	-.03	.05
Volunteering	Main effect of moderator	-.04	.02	-.04	-1.80	.072	1.00	-.08	.00
	Interaction with T1	.05	.02	.05	2.25	.024	1.00	.01	.09

Note: Scores were corrected for mean age and sex differences (see Methods). Bonferroni correction for multiple comparisons was applied to obtain corrected p values.

Supplementary Table 32. Multiple regression analysis of phenotypic moderation of having children (continuous variable) on T2 responses.

**Construct**		Unstandardised Coefficients		Standardised Coefficients				95% Confidence Interval for B	
		B	SE	Beta	*t*	*p*	$p_{corrected}$	Lower bound	Upper bound
Love and relationships	Main effect of moderator	.00	.02	.00	-.02	.983	1.00	-.05	.05
	Interaction with T1	.01	.03	.01	0.44	.663	1.00	-.04	.06
Achievement motivation	Main effect of moderator	.05	.02	.05	2.60	.010	1.00	.01	.08
	Interaction with T1	-.01	.02	-.01	-0.28	.782	1.00	-.04	.03
Importance of Relationships	Main effect of moderator	-.02	.02	-.02	-1.14	.254	1.00	-.05	.01
	Interaction with T1	-.07	.02	-.06	-3.01	.003	1.00	-.11	-.02
Purpose in Life	Main effect of moderator	.04	.02	.05	2.67	.008	1.00	.01	.08
	Interaction with T1	-.01	.02	-.01	-0.64	.520	1.00	-.04	.02
Healthcare	Main effect of moderator	.00	.02	.00	0.21	.834	1.00	-.03	.04
	Interaction with T1	-.04	.02	-.04	-1.87	.061	1.00	-.08	.00

Community satisfaction	Main effect of moderator	.00	.02	.00	-0.20	.840	1.00	-.05	.04
	Interaction with T1	-.04	.02	-.04	-1.73	.085	1.00	-.08	.00
Attitudes towards money	Main effect of moderator	.00	.02	.00	0.18	.859	1.00	-.04	.04
	Interaction with T1	-.01	.02	-.01	-0.60	.550	1.00	-.05	.03
Alcohol (frequency x quantity)	Main effect of moderator	-.04	.03	-.04	-1.62	.106	1.00	-.09	.01
	Interaction with T1	-.06	.03	-.06	-2.34	.019	1.00	-.11	-.01
Conduct problems	Main effect of moderator	.07	.02	.07	3.29	.001	1.00	.03	.11
	Interaction with T1	.04	.02	.05	2.16	.031	1.00	.00	.08
Emotional problems	Main effect of moderator	.02	.02	.02	0.92	.356	1.00	-.02	.06
	Interaction with T1	.01	.02	.02	0.83	.409	1.00	-.02	.05
Hyperactivity	Main effect of moderator	-.05	.02	-.05	-2.38	.017	1.00	-.09	-.01
	Interaction with T1	.02	.02	.02	.91	.364	1.00	-.02	.06
Peer problems	Main effect of moderator	.00	.02	.00	0.06	.956	1.00	-.04	.04
	Interaction with T1	.07	.02	.08	3.65	.000	1.00	.03	.11
Prosocial behaviour	Main effect of moderator	.00	.02	.00	-0.07	.942	1.00	-.04	.04
	Interaction with T1	.00	.02	.00	0.14	.892	1.00	-.04	.05
General anxiety	Main effect of moderator	.06	.02	.06	3.19	.001	1.00	.02	.10
	Interaction with T1	.06	.02	.07	3.32	.001	1.00	.02	.10
Depression	Main effect of moderator	.00	.02	.00	-0.01	.992	1.00	-.04	.04
	Interaction with T1	-.01	.02	-.01	-0.40	.692	1.00	-.04	.03
Physical activity	Main effect of moderator	-.08	.02	-.08	-3.76	.000	1.00	-.12	-.04
	Interaction with T1	-.02	.02	-.02	-1.09	.274	1.00	-.06	.02
Media use	Main effect of moderator	-.03	.02	-.03	-1.41	.160	1.00	-.07	.01
	Interaction with T1	-.01	.02	-.01	-0.65	.515	1.00	-.06	.03
Volunteering	Main effect of moderator	.04	.02	.04	1.75	.080	1.00	.00	.09
	Interaction with T1	.03	.03	.03	1.29	.198	1.00	-.02	.08

Note: Scores were corrected for mean age and sex differences (see Methods). Bonferroni correction for multiple comparisons was applied to obtain corrected p values.

Supplementary Table 33. Multiple regression analysis of phenotypic moderation of having children (yes/no) on T2 responses.

Construct		Unstandardised Coefficients		Standardised Coefficients				95% Confidence Interval for B	
		B	SE	Beta	*t*	*p*	$p_{corrected}$	Lower bound	Upper bound
Love and relationships	Main effect of moderator	.01	.02	.01	.34	.734	1.00	-.04	.05
	Interaction with T1	.01	.02	.01	0.32	.747	1.00	-.04	.05
Achievement motivation	Main effect of moderator	.03	.02	.04	1.90	.057	1.00	.00	.07
	Interaction with T1	-.02	.02	-.02	-1.09	.275	1.00	-.05	.02
Importance of Relationships	Main effect of moderator	-.01	.02	-.01	-0.61	.542	1.00	-.05	.02
	Interaction with T1	-.06	.02	-.06	-3.12	.002	1.00	-.10	-.02
Purpose in Life	Main effect of moderator	.05	.02	.06	3.24	.001	1.00	.02	.09
	Interaction with T1	-.02	.02	-.03	-1.50	.133	1.00	-.05	.01
Healthcare	Main effect of moderator	-.01	.02	-.01	-0.40	.687	1.00	-.05	.03
	Interaction with T1	-.05	.02	-.06	-2.50	.012	1.00	-.09	-.01
Community satisfaction	Main effect of moderator	-.01	.02	-.01	-0.36	.719	1.00	-.05	.03
	Interaction with T1	-.02	.02	-.02	-1.10	.273	1.00	-.06	.02
Attitudes towards money	Main effect of moderator	-.01	.02	-.01	-0.27	.791	1.00	-.05	.04
	Interaction with T1	-.04	.02	-.04	-1.78	.076	1.00	-.08	.00
Alcohol (frequency x quantity)	Main effect of moderator	-.04	.02	-.04	-1.89	.059	1.00	-.09	.00
	Interaction with T1	-.05	.02	-.05	-2.06	.039	1.00	-.09	.00
Conduct problems	Main effect of moderator	.07	.02	.07	3.27	.001	1.00	.03	.11
	Interaction with T1	.03	.02	.04	1.63	.103	1.00	-.01	.07
Emotional problems	Main effect of moderator	.01	.02	.01	0.49	.624	1.00	-.03	.05
	Interaction with T1	.01	.02	.01	0.69	.489	1.00	-.02	.05
Hyperactivity	Main effect of moderator	-.05	.02	-.05	-2.54	.011	1.00	-.09	-.01
	Interaction with T1	.02	.02	.02	.91	.363	1.00	-.02	.06
Peer problems	Main effect of moderator	.00	.02	.00	-0.16	.877	1.00	-.04	.04
	Interaction with T1	.07	.02	.07	3.41	.001	1.00	.03	.10
	Main effect of moderator	.00	.02	.00	0.16	.876	1.00	-.04	.04

Prosocial behaviour	Interaction with T1	.00	.02	.00	-0.17	.866	1.00	-.05	.04
General anxiety	Main effect of moderator	.05	.02	.05	2.57	.010	1.00	.01	.09
	Interaction with T1	.06	.02	.07	3.36	.001	1.00	.03	.10
Depression	Main effect of moderator	-.01	.02	-.01	-0.26	.796	1.00	-.04	.03
	Interaction with T1	-.01	.02	-.02	-0.80	.421	1.00	-.05	.02
Physical activity	Main effect of moderator	-.09	.02	-.09	-4.08	.000	.325	-.13	-.04
	Interaction with T1	-.01	.02	-.01	-0.51	.612	1.00	-.05	.03
Media use	Main effect of moderator	-.04	.02	-.04	-2.02	.043	1.00	-.08	.00
	Interaction with T1	-.02	.02	-.02	-0.78	.436	1.00	-.06	.02
Volunteering	Main effect of moderator	.04	.02	.04	1.58	.113	1.00	-.01	.08
	Interaction with T1	.03	.02	.03	1.31	.189	1.00	-.02	.08

Note: Scores were corrected for mean age and sex differences (see Methods). Bonferroni correction for multiple comparisons was applied to obtain corrected p values.

Supplementary Table 34. Twin intraclass correlations and Falconer ACE estimates for T1.

**Construct**	**T1**								
	**MZ**			**DZ**			**Falconer ACE estimates**		
	r	*p*	*n*	*r*	*p*	*n*	A	C	E
Love and relationships	.19	<.001	929	.08	.005	1351	.19	.00	.81
Achievement motivation	.37	<.001	923	.16	<.001	1361	.37	.00	.63
Importance of Relationships	.42	<.001	923	.10	<.001	1361	.42	.00	.58
Purpose in Life	.38	<.001	922	.21	<.001	1361	.34	.05	.62
Healthcare	.36	<.001	922	.16	<.001	1354	.36	.00	.65
Community satisfaction	.42	<.001	926	.35	<.001	1339	.14	.28	.58
Attitudes towards money	.26	<.001	894	.05	.059	1274	.26	.00	.74
Alcohol (frequency x quantity)	.36	<.001	793	.15	<.001	1139	.36	.00	.64
Conduct problems	.28	<.001	901	.12	<.001	1331	.28	.00	.72
Emotional problems	.36	<.001	901	.18	<.001	1331	.34	.01	.65
Hyperactivity	.39	<.001	901	.16	<.001	1331	.39	.00	.61

Peer problems	.39	<.001	901	.24	<.001	1331	.30	.09	.61
Prosocial behaviour	.28	<.001	901	.11	<.001	1331	.28	.00	.72
General anxiety	.38	<.001	847	.14	<.001	1214	.38	.00	.63
Depression	.35	<.001	901	.18	<.001	1331	.33	.02	.65
Physical activity	.41	<.001	918	.18	<.001	1325	.41	.00	.59
Media use	.41	<.001	918	.18	<.001	1325	.41	.00	.59
Volunteering	.35	<.001	900	.13	<.001	1331	.35	.00	.65

Note: MZ=monozygotic; DZ=dizygotic; and genetic (A), shared environmental (C) and non-shared environmental (E) estimates. Scores were corrected for mean age and sex differences (see Methods). ACE estimates were derived from the intraclass twin correlations using Falconer's formula (Rijsdijk & Sham, 2002).

Supplementary Table 35. Twin intraclass correlations and Falconer ACE estimates for T2.

Construct	T2								
	MZ			DZ			Falconer ACE estimates		
	r	*p*	*n*	*r*	*p*	*n*	A	C	E
Love and relationships	.25	<.001	530	.07	.077	600	.25	.00	.75
Achievement motivation	.39	<.001	548	.17	<.001	625	.39	.00	.61
Importance of Relationships	.39	<.001	548	.14	.001	625	.39	.00	.61
Purpose in Life	.43	<.001	552	.27	<.001	625	.32	.11	.57
Healthcare	.20	<.001	552	.13	.001	625	.14	.06	.80
Community satisfaction	.29	<.001	552	.11	.005	625	.29	.00	.71
Attitudes towards money	.19	<.001	552	.04	.304	625	.19	.00	.81
Alcohol (frequency x quantity)	.39	<.001	550	.23	<.001	620	.32	.07	.61
Conduct problems	.17	<.001	550	.18	<.001	620	.00	.17	.83
Emotional problems	.39	<.001	550	.20	<.001	620	.38	.01	.61
Hyperactivity	.36	<.001	550	.09	.027	620	.36	.00	.64
Peer problems	.42	<.001	550	.23	<.001	620	.38	.04	.58
Prosocial behaviour	.35	<.001	550	.19	<.001	620	.32	.03	.65
General anxiety	.42	<.001	550	.19	<.001	620	.42	.00	.58
Depression	.38	<.001	550	.18	<.001	620	.38	.00	.62
Physical activity	.40	<.001	549	.27	<.001	620	.26	.14	.60
Media use	.49	<.001	549	.14	.001	620	.49	.00	.51
Volunteering	.38	<.001	549	.09	.023	620	.38	.00	.62

Note: MZ=monozygotic; DZ=dizygotic; and genetic (A), shared environmental (C) and non-shared environmental (E) estimates. Scores were corrected for mean age and sex differences (see Methods). ACE estimates were derived from the intraclass twin correlations using Falconer's formula (Rijsdijk & Sham, 2002).

Supplementary Table 36. Twin intraclass correlations and Falconer ACE estimates for T2 change.

Construct	T2 change								
	MZ			DZ			Falconer ACE estimates		
	r	*p*	*n*	*r*	*p*	*n*	A	C	E
Love and relationships	.17	<.001	482	.05	.305	530	.17	.00	.83
Achievement motivation	.16	<.001	492	.06	.168	544	.16	.00	.84
Importance of Relationships	.12	.010	492	.00	.997	544	.12	.00	.88
Purpose in Life	.13	.003	495	.12	.007	544	.02	.11	.87
Healthcare	.11	.018	495	.07	.099	543	.08	.03	.89
Community satisfaction	.20	<.001	499	-.03	.482	547	.20	.00	.80
Attitudes towards money	.12	.008	488	-.01	.914	521	.12	.00	.88
Alcohol (frequency x quantity)	.22	<.001	446	.03	.505	476	.22	.00	.78
Conduct problems	.14	.003	484	.12	.005	538	.04	.10	.86
Emotional problems	.25	<.001	484	.06	.194	538	.25	.00	.75
Hyperactivity	.20	<.001	484	.09	.036	538	.20	.00	.80
Peer problems	.19	<.001	484	.08	.066	538	.19	.00	.81
Prosocial behaviour	.15	.001	484	.13	.003	538	.04	.11	.85
General anxiety	.25	<.001	475	.10	.021	513	.25	.00	.75
Depression	.13	.003	484	.08	.062	538	.10	.03	.87
Physical activity	.25	<.001	493	.17	<.001	538	.16	.09	.75
Media use	.32	<.001	493	.05	.244	534	.32	.00	.68
Volunteering	.31	<.001	483	.07	.106	538	.31	.00	.69

Note: MZ=monozygotic; DZ=dizygotic; and genetic (A), shared environmental (C) and non-shared environmental (E) estimates. Scores were corrected for mean age and sex differences (see Methods). ACE estimates were derived from the intraclass twin correlations using Falconer's formula (Rijsdijk & Sham, 2002).

Supplementary Table 37. Whole sample model fitting results for univariate analyses of additive genetic (A), shared environmental (C), and non-shared environmental (E) components of variance for variables (95% confidence intervals in parentheses).

Construct	T1			T2			T2 change		
	A	C	E	A	C	E	A	C	E
Love and relationships	.18 (.07-.24)	.00 (.00-.00)	.82 (.76-.87)	.23 (.09-.30)	.00 (.00-.00)	.77 (.70-.85)	.15 (.00-.23)	.00 (.00-.13)	.85 (.77-.92)
Achievement motivation	.36 (.26-.41)	.00 (.00-.00)	.64 (.59-.69)	.38 (.21-.44)	.00 (.00-.13)	.62 (.56-.68)	.15 (.07-.23)	.00 (.00-.14)	.85 (.77-.93)
Importance of Relationships	.38 (.32-.43)	.00 (.00-.02)	.62 (.57-.67)	.35 (.21-.41)	.00 (.00-.11)	.65 (.59-.71)	.09 (.05-.17)	.00 (.00-.10)	.91 (.83-.95)
Purpose in Life	.37 (.22-.45)	.03 (.00-.14)	.61 (.55-.66)	.41 (.22-.52)	.05 (.00-.20)	.54 (.48-.61)	.05 (.00-.23)	.09 (.00-.18)	.86 (.77-.93)
Healthcare	.36 (.26-.41)	.00 (.00-.07)	.64 (.59-.69)	.14 (.00-.30)	.08 (.00-.23)	.78 (.70-.87)	.08 (.00-.20)	.03 (.00-.15)	.89 (.80-.97)
Community satisfaction	.13 (.00-.26)	.29 (.18-.39)	.58 (.53-.63)	.29 (.15-.35)	.00 (.00-.10)	.71 (.64-.78)	.14 (.05-.22)	.00 (.00-.06)	.86 (.78-.94)
Attitudes towards money	.22 (.15-.28)	.00 (.00-.04)	.78 (.72-.83)	.16 (.03-.23)	.00 (.00-.00)	.84 (.77-.91)	.09 (.00-.17)	.00 (.00-.09)	.91 (.83-.99)
Alcohol (frequency x quantity)	.35 (.25-.41)	.00 (.00-.00)	.65 (.59-.70)	.40 (.20-.49)	.03 (.00-.18)	.58 (.51-.65)	.19 (.06-.28)	.00 (.00-.09)	.81 (.72-.89)
Conduct problems	.27 (.17-.33)	.00 (.00-.08)	.73 (.67-.78)	.01 (.00-.24)	.19 (.01-.25)	.81 (.72-.87)	.04 (.00-.23)	.10 (.00-.19)	.86 (.77-.93)

Emotional problems	.33 (.18-.40)	.02 (.00-.13)	.65 (.60-.71)	.39 (.20-.45)	.00 (.00-.15)	.61 (.55-.68)	.22 (.10-.30)	.00 (.00-.09)	.78 (.70-.86)
Hyperactivity	.38 (.29-.42)	.00 (.00-.07)	.62 (.58-.67)	.34 (.25-.40)	.00 (.00-.00)	.66 (.60-.73)	.20 (.00-.28)	.00 (.00-.15)	.80 (.72-.87)
Peer problems	.29 (.15-.43)	.09 (.00-.20)	.61 (.56-.67)	.36 (.16-.48)	.06 (.00-.22)	.58 (.52-.65)	.18 (.00-.26)	.00 (.00-.18)	.82 (.74-.90)
Prosocial behaviour	.27 (.15-.32)	.00 (.00-.09)	.73 (.68-.79)	.34 (.14-.42)	.01 (.00-.17)	.64 (.58-.72)	.03 (.00-.23)	.12 (.00-.20)	.85 (.77-.91)
General anxiety	.36 (.28-.41)	.00 (.00-.06)	.64 (.59-.69)	.39 (.20-.45)	.00 (.00-.15)	.61 (.55-.68)	.26 (.11-.33)	.00 (.00-.11)	.74 (.67-.82)
Depression	.29 (.13-.39)	.05 (.00-.16)	.67 (.61-.72)	.39 (.24-.45)	.00 (.00-.12)	.61 (.55-.68)	.12 (.00-.22)	.02 (.00-.16)	.86 (.78-.92)
Physical activity	.40 (.29-.44)	.00 (.00-.08)	.60 (.56-.65)	.30 (.10-.46)	.12 (.00-.27)	.58 (.52-.66)	.17 (.00-.34)	.09 (.00-.26)	.74 (.66-.83)
Media use	.45 (.35-.50)	.00 (.00-.08)	.55 (.50-.60)	.44 (.35-.49)	.00 (.00-.00)	.56 (.51-.62)	.26 (.16-.33)	.00 (.00-.08)	.74 (.67-.81)
Volunteering	.32 (.23-.37)	.00 (.00-.00)	.68 (.63-.73)	.35 (.26-.41)	.00 (.00-.00)	.65 (.59-.72)	.28 (.19-.36)	.00 (.00-.06)	.72 (.64-.79)

Note: Scores were corrected for mean age and sex differences (see Methods).

Supplementary Table 38. Bivariate Cholesky decomposition estimating the aetiology of the association between T1 (A1, C1 and E1) and T2 (A2, C2 and E2), for variable in the relationships theme (95% confidence intervals in parentheses).

Trait	A1	A2
Love and relationships	0.183 (0.070,0.236)	
Love and relationships	0.173(0.089,0.305)	0.077 (0.000,0.147)
	C1	C2
Love and relationships	0.000 (0.000,0.082)	
Love and relationships	0.000 (0.000,0.072)	0.000 (0.000,0.071)
	E1	E2
Love and relationships	0.815 (0.749,0.882)	
Love and relationships	0.083 (0.056,0.114)	0.666 (0.595,0.740)

Note: Scores were corrected for mean age and sex differences (see Methods).

Supplementary Table 39. Bivariate Cholesky decomposition estimating the aetiology of the association between T1 (A1, C1 and E1) and T2 (A2, C2 and E2), for variables in the thoughts and attitudes theme (95% confidence intervals in parentheses).

(a)		
Trait	A1	A2
Achievement motivation	0.348 (0.245,0.393)	
Achievement motivation	0.328 (0.192,0.407)	0.043 (0.000,0.101)
	C1	C2
Achievement motivation	0.000 (0.000,0.076)	
Achievement motivation	0.000 (0.000,0.137)	0.000 (0.000,0.066)
	E1	E2
Achievement motivation	0.630 (0.573,0.690)	
Achievement motivation	0.092 (0.066,0.122)	0.531 (0.471,0.594)
(b)		
Trait	A1	A2
Importance of relationships	0.376 (0.324,0.422)	
Importance of relationships	0.361 (0.285,0.412)	0.000 (0.000,0.041)
	C1	C2
Importance of relationships	0.000 (0.000,0.027)	
Importance of relationships	0.000 (0.000,0.060)	0.000 (0.000,0.029)
	E1	E2
Importance of relationships	0.626 (0.567,0.688)	
Importance of relationships	0.100 (0.075,0.129)	0.537 (0.488,0.587)
(c)		
Trait	A1	A2
Purpose in life	0.385 (0.250,0.450)	

Purpose in life	0.432 (0.265,0.516)	0.000 (0.000,0.091)
	C1	C2
Purpose in life	0.020 (0.000,0.124)	
Purpose in life	0.001 (0.000,0.154)	0.028 (0.000,0.076)
	E1	E2
Purpose in life	0.602 (0.543,0.665)	
Purpose in life	0.104 (0.077,0.138)	0.436 (0.386,0.490)
(d)		
Trait	A1	A2
Healthcare	0.376 (0.274,0.426)	
Healthcare	0.146 (0.057,0.245)	0.049 (0.000,0.150)
	C1	C2
Healthcare	0.000 (0.000,0.073)	
Healthcare	0.009 (0.000,0.144)	0.010 (0.000,0.114)
	E1	E2
Healthcare	0.678 (0.614,0.745)	
Healthcare	0.019 (0.006,0.039)	0.830 (0.741,0.918)
(e)		
Trait	A1	A2
Community satisfaction	0.128 (0.007,0.255)	
Community satisfaction	0.098 (0.000,0.279)	0.087 (0.000,0.160)
	C1	C2
Community satisfaction	0.284 (0.184,0.378)	
Community satisfaction	0.074 (0.018,0.159)	0.000 (0.000,0.051)
	E1	E2
Community satisfaction	0.566 (0.509,0.628)	
Community satisfaction	0.049 (0.027,0.078)	0.687 (0.613,0.764)
(f)		
Trait	A1	A2
Money attitudes	0.220 (0.150,0.273)	
Money attitudes	0.117 (0.046,0.200)	0.063 (0.000,0.132)
	C1	C2
Money attitudes	0.000 (0.000,0.044)	
Money attitudes	0.000 (0.000,0.098)	0.000 (0.000,0.076)
	E1	E2
Money attitudes	0.782 (0.716,0.849)	
Money attitudes	0.064 (0.040,0.094)	0.754 (0.678,0.833)

Note: Scores were corrected for mean age and sex differences (see Methods).

Supplementary Table 40. Bivariate Cholesky decomposition estimating the aetiology of the association between T1 (A1, C1 and E1) and T2 (A2, C2 and E2), for variables in the substance use theme (95% confidence intervals in parentheses).

Trait	A1	A2
Alcohol freq x quant	0.379 (0.261,0.431)	
Alcohol freq x quant	0.257 (0.127,0.337)	0.157 (0.049,0.223)
	C1	C2
Alcohol freq x quant	0.000 (0.000,0.088)	
Alcohol freq x quant	0.000 (0.000,0.139)	0.000 (0.000,0.074)
	E1	E2
Alcohol freq x quant	0.667 (0.602,0.736)	
Alcohol freq x quant	0.042 (0.024,0.066)	0.565 (0.499,0.637)

Note: Scores were corrected for mean age and sex differences (see Methods).

Supplementary Table 41. Bivariate Cholesky decomposition estimating the aetiology of the association between T1 (A1, C1 and E1) and T2 (A2, C2 and E2), for variables in the wellbeing theme (95% confidence intervals in parentheses).

(a)		
Trait	A1	A2
Conduct problems	0.308 (0.192,0.363)	
Conduct problems	0.109 (0.016,0.234)	0.000 (0.000,0.157)
	C1	C2
Conduct problems	0.000(0.000,0.083)	
Conduct problems	0.082 (0.000,0.195)	0.016 (0.000,0.150)
	E1	E2
Conduct problems	0.783(0.713,0.855)	
Conduct problems	0.038 (0.018,0.067)	0.798(0.718,0.873)
(b)		
Trait	A1	A2
Emotional problems	0.300 (0.160,0.390)	
Emotional problems	0.253 (0.122,0.403)	0.101 (0.000,0.161)
	C1	C2
Emotional problems	0.041 (0.000,0.147)	
Emotional problems	0.032 (0.000,0.148)	0.000 (0.000,0.065)
	E1	E2
Emotional problems	0.656 (0.594,0.721)	
Emotional problems	0.078 (0.053,0.110)	0.537 (0.476,0.604)
(c)		
Trait	A1	A2
Hyperactivity	0.355 (0.250,0.425)	
Hyperactivity	0.275 (0.165,0.384)	0.050 (0.000,0.185)

	C1	C2
Hyperactivity	0.024 (0.000,0.100)	
Hyperactivity	0.016 (0.000,0.091)	0.000 (0.000,0.080)
	E1	E2
Hyperactivity	0.632 (0.571,0.697)	
Hyperactivity	0.037 (0.020,0.062)	0.626 (0.554,0.702)
(d)		
Trait	A1	A2
Peer problems	0.298 (0.161,0.420)	
Peer problems	0.272 (0.133,0.449)	0.066 (0.000,0.123)
	C1	C2
Peer problems	0.088 (0.000,0.194)	
Peer problems	0.072 (0.000,0.204)	0.000 (0.000,0.085)
	E1	E2
Peer problems	0.603 (0.544,0.666)	
Peer problems	0.069 (0.045,0.099)	0.512 (0.453,0.576)
(e)		
Trait	A1	A2
Prosocial behaviour	0.272 (0.176,0.323)	
Prosocial behaviour	0.327 (0.184,0.419)	0.00 (-0.130,0.125)
	C1	C2
Prosocial behaviour	0.001 (0.000,0.072)	
Prosocial behaviour	0.036 (0.000,0.151)	0.000 (0.000,0.105)
	E1	E2
Prosocial behaviour	0.747 (0.684,0.812)	
Prosocial behaviour	0.042 (0.025,0.065)	0.597 (0.532,0.665)
(f)		
Trait	A1	A2
General anxiety	0.358 (0.271,0.407)	
General anxiety	0.332 (0.235,0.434)	0.108 (0.000,0.169)
	C1	C2
General anxiety	0.000 (0.000,0.061)	
General anxiety	0.000 (0.000,0.091)	0.000 (0.000,0.070)
	E1	E2
General anxiety	0.651 (0.589,0.716)	
General anxiety	0.084 (0.059,0.114)	0.476 (0.418,0.540)
(g)		
Trait	A1	A2
Depression	0.323 (0.202,0.393)	
Depression	0.390 (0.251,0.451)	0.000 (0.000,0.071)
	C1	C2
Depression	0.023 (0.000,0.121)	
Depression	0.004 (0.000,0.106)	0.000 (0.000,0.048)

	E1	E2
Depression	0.654 (0.596,0.714)	
Depression	0.048 (0.030,0.072)	0.560 (0.499,0.623)
(h)		
Trait	A1	A2
Physical activity	0.344 (0.239,0.420)	
Physical activity	0.107 (0.024,0.221)	0.084 (0.000,0.201)
	C1	C2
Physical activity	0.048 (0.002,0.125)	
Physical activity	0.198 (0.038,0.332)	0.000 (0.000,0.130)
	E1	E2
Physical activity	0.623 (0.564,0.687)	
Physical activity	0.056 (0.033,0.084)	0.559 (0.491,0.635)

Note: Scores were corrected for mean age and sex differences (see Methods).

Supplementary Table 42. Bivariate Cholesky decomposition estimating the aetiology of the association between T1 (A1, C1 and E1) and T2 (A2, C2 and E2), for variables in the behaviours theme (95% confidence intervals in parentheses).

(a)		
Trait	A1	A2
Media use	0.454 (0.353,0.496)	
Media use	0.262 (0.192,0.349)	0.178 (0.079,0.236)
	C1	C2
Media use	0.000 (0.000,0.078)	
Media use	0.000 (0.000,0.065)	0.000 (0.000,0.054)
	E1	E2
Media use	0.543 (0.489,0.600)	
Media use	0.031 (0.016,0.051)	0.535 (0.474,0.600)
(b)		
Trait	A1	A2
Volunteering	0.312 (0.209,0.358)	
Volunteering	0.073 (0.039,0.174)	0.255 (0.108,0.321)
	C1	C2
Volunteering	0.000 (0.000,0.076)	
Volunteering	0.00 (-0.060,0.056)	0.000 (0.000,0.055)
	E1	E2
Volunteering	0.659 (0.601,0.720)	
Volunteering	0.025 (0.012,0.044)	0.656 (0.581,0.750)

Note: Scores were corrected for mean age and sex differences (see Methods).

Supplementary Table 43. Bivariate model fit indices

	base	comparison	ep	minus2LL	df	AIC	diffLL	diffdf	p
Healthcare	Sat	<NA>	28	24260.62	8959	6342.62200			
Healthcare	Sat	ACE	11	24354.20	8976	6402.19600	93.57320	17<.00100	0.00000
Community satisfaction	Sat	<NA>	28	24346.90	8931	6484.90300			
Community satisfaction	Sat	ACE	11	24361.32	8948	6465.32100	14.41835	17<.00100	0.63730
Achievement motivation	Sat	<NA>	28	23717.14	8962	5793.14300			
Achievement motivation	Sat	ACE	11	23748.20	8979	5790.20400	31.06116	17<.00100	0.01963
Importance of relationships	Sat	<NA>	28	23417.91	8963	5491.91400			
Importance of relationships	Sat	ACE	11	23476.18	8980	5516.18100	58.26745	17<.00100	0.00000
Depression	Sat	<NA>	28	23671.89	8888	5895.89200			
Depression	Sat	ACE	11	23720.46	8905	5910.45700	48.56479	17<.00100	0.00007
Money attitudes	Sat	<NA>	28	24400.41	8810	6780.41400			
Money attitudes	Sat	ACE	11	24418.71	8827	6764.70700	18.29351	17<.00100	0.37055
Purpose in life	Sat	<NA>	28	23004.94	8966	5072.93500			
Purpose in life	Sat	ACE	11	23034.71	8983	5068.71400	29.77842	17<.00100	0.02799
Love and relationships	Sat	<NA>	28	24420.83	8883	6654.82800			
Love and relationships	Sat	ACE	11	24434.09	8900	6634.08700	13.25833	17<.00100	0.71873
Conduct problems	Sat	<NA>	28	24185.75	8891	6403.75000			
Conduct problems	Sat	ACE	11	24207.22	8908	6391.21600	21.46592	17<.00100	0.20614
Hyperactivity	Sat	<NA>	28	24153.29	8891	6371.29500			
Hyperactivity	Sat	ACE	11	24191.58	8908	6375.58100	38.28626	17<.00100	0.00224
Peer problems	Sat	<NA>	28	36520.94	13526	9468.94000			
Peer problems	Sat	ACE	11	23575.74	8908	5759.74100	13.34099	17<.00100	0.71308
Prosocial behaviour	Sat	<NA>	28	24161.20	8891	6379.19900			
Prosocial behaviour	Sat	ACE	11	24177.12	8908	6361.12100	15.92163	17<.00100	0.52940
Alcohol freq x quant	Sat	<NA>	28	22728.22	8451	5826.21600			
Alcohol freq x quant	Sat	ACE	11	22755.08	8468	5819.07900	26.86278	17<.00100	0.06011

General anxiety	Sat	<NA>	28	22822.96	8633	5556.95900			
General anxiety	Sat	ACE	11	22867.06	8650	5567.06300	44.10331	17<.00100	0.00033
Physical activity	Sat	<NA>	28	23956.38	8879	6198.38100			
Physical activity	Sat	ACE	11	23977.79	8896	6185.79100	21.40986	17<.00100	0.20849
Media use	Sat	<NA>	28	23833.70	8862	6109.69600			
Media use	Sat	ACE	11	23855.84	8879	6097.84000	22.14444	17<.00100	0.17923
Volunteering	Sat	<NA>	28	24837.20	8883	7071.20100			
Volunteering	Sat	ACE	11	24864.09	8900	7064.08800	26.88685	17<.00100	0.05975
Emotional problems	Sat	<NA>	28	23762.35	8891	5980.35500			
Emotional problems	Sat	ACE	11	23781.53	8908	5965.53100	19.17643	17<.00100	0.31850

Note: Scores were corrected for mean age and sex differences (see Methods).

Supplementary Table 44. For twins living together: T1 twin intraclass correlations and model fitting results for univariate analyses of additive genetic (A), shared environmental (C), and non-shared environmental (E) components of variance (95% confidence intervals in parentheses).

**Construct**	**T1**											
	MZ DZ combined			MZ			DZ					
	r	*p*	*n*	*r*	*p*	*n*	*r*	*p*	*n*	A	C	E
Love and relationships	.14	.011	312	.18	.008	219	-.03	.713	218	.18(.07-.24)	.00(.00-.00)	.82(.76-.87)
Achievement motivation	.25	<.001	331	.47	<.001	219	.14	.046	219	.36(.26-.41)	.00(.00-.00)	.64(.59-.69)
Importance of Relationships	.25	<.001	331	.47	<.001	219	.07	.304	219	.38(.32-.43)	.00(.00-.02)	.62(.57-.67)

Purpose in Life	.32	<.001	332	.39	<.001	218	.27	<.001	219	.37(.22-.45)	.03(.00-.14)	.61(.55-.66)
Healthcare	.25	<.001	332	.37	<.001	217	.30	<.001	216	.36(.26-.41)	.00(.00-.07)	.64(.59-.69)
Community satisfaction	.41	<.001	332	.46	<.001	218	.37	<.001	218	.13(.00-.26)	.29(.18-.39)	.58(.53-.63)
Attitudes towards money	.18	.001	332	.35	<.001	213	.07	.289	208	.22(.15-.28)	.00(.00-.04)	.78(.72-.83)
Alcohol (frequency x quantity)	.41	<.001	331	.30	<.001	428	.14	<.001	623	.35(.25-.41)	.00(.00-.00)	.65(.59-.70)
Conduct problems	.18	.001	330	.30	<.001	470	.16	<.001	726	.27(.17-.33)	.00(.00-.08)	.73(.67-.78)
Emotional problems	.37	<.001	330	.38	<.001	209	.16	.016	216	.33(.18-.40)	.02(.00-.13)	.65(.60-.71)
Hyperactivity	.25	<.001	330	.48	<.001	209	.17	.011	216	.38(.29-.42)	.00(.00-.07)	.62(.58-.67)
Peer problems	.43	<.001	330	.33	<.001	470	.25	<.001	726	.29(.15-.43)	.09(.00-.20)	.61(.56-.67)
Prosocial behaviour	.24	<.001	330	.30	<.001	470	.12	.001	726	.27(.15-.32)	.00(.00-.09)	.73(.68-.79)
General anxiety	.35	<.001	330	.33	<.001	207	.24	<.001	213	.36(.28-.41)	.00(.00-.06)	.64(.59-.69)
Depression	.34	<.001	330	.32	<.001	470	.17	<.001	726	.29(.13-.39)	.05(.00-.16)	.67(.61-.72)
Physical activity	.38	<.001	329	.55	<.001	218	.21	.002	215	.40(.29-.44)	.00(.00-.08)	.60(.56-.65)
Media use	.44	<.001	329	.64	<.001	217	.29	<.001	209	.45(.35-.50)	.00(.00-.08)	.55(.50-.60)
Volunteering	.28	<.001	329	.42	<.001	209	.21	.002	216	.32(.23-.37)	.00(.00-.00)	.68(.63-.73)

Note: Scores were corrected for mean age and sex differences (see Methods).

Supplementary Table 45. For twins living together: T2 twin intraclass correlations and model fitting results for univariate analyses of additive genetic (A), shared environmental (C), and non-shared environmental (E) components of variance (95% confidence intervals in parentheses).

**Construct**	**T2**											
	MZ DZ combined			MZ			DZ					
	r	*p*	*n*	*r*	*p*	*n*	*r*	*p*	*n*	A	C	E
Love and relationships	.14	.011	312	.24	.002	163	.04	.608	145	.23 (.00-.36)	.00 (NA-.22)	.77 (.64-.91)
Achievement motivation	.25	<.001	331	.37	<.001	176	.10	.215	151	.36 (.23-.47)	.00 (.00-.19)	.64 (.53-.77)
Importance of Relationships	.25	<.001	331	.40	<.001	176	.01	.920	151	.34 (.13-.44)	.00 (NA-.17)	.66 (.56-.78)
Purpose in Life	.32	<.001	332	.36	<.001	177	.29	<.001	151	.21 (.00-.51)	.19 (.00-.42)	.60 (.48-.74)
Healthcare	.25	<.001	332	.14	.068	177	.36	<.001	151	.00 (NA-.25)	.29 (.18-.40)	.71 (.60-.82)
Community satisfaction	.41	<.001	332	.48	<.001	177	.31	<.001	151	.28 (.00-.56)	.18 (.00-.45)	.54 (.44-.65)
Attitudes towards money	.18	.001	332	.33	<.001	177	.01	.943	151	.26 (.06-.38)	.00 (NA-.14)	.74 (.62-.87)
Alcohol (frequency x quantity)	.41	<.001	331	.50	<.001	177	.32	<.001	150	.39 (.07-.62)	.14 (.00-.40)	.47 (.38-.59)
Conduct problems	.18	.001	330	.19	.013	177	.18	.025	149	.08 (NA-.34)	.12 (.00-.28)	.80 (.66-.92)
Emotional problems	.37	<.001	330	.46	<.001	177	.26	.001	149	.40 (.06-.57)	.07 (NA-.29)	.53 (.43-.65)
Hyperactivity	.25	<.001	330	.34	<.001	177	.15	.065	149	.35 (.07-.47)	.00 (NA-.21)	.65 (.53-.78)

Peer problems	.43	<.001	330	.52	<.001	177	.29	<.001	149	.36 (.02-.58)	.14 (.00-.42)	.51 (.41-.61)
Prosocial behaviour	.24	<.001	330	.40	<.001	177	.08	.350	149	.37 (.18-.48)	.00 (.00-.13)	.63 (.52-.76)
General anxiety	.35	<.001	330	.51	<.001	177	.21	.010	149	.53 (.33-.62)	.00 (NA-.15)	.47 (.38-.58)
Depression	.34	<.001	330	.43	<.001	177	.22	.007	149	.46 (.14-.56)	.00 (.00-.26)	.54 (.44-.65)
Physical activity	.38	<.001	329	.40	<.001	176	.34	<.001	149	.17 (.00-.50)	.25 (.00-.45)	.58 (.47-.70)
Media use	.44	<.001	329	.58	<.001	176	.26	.001	149	.54 (.22-.63)	.01 (.00-.28)	.45 (.37-.55)
Volunteering	.28	<.001	329	.40	<.001	176	.13	.124	149	.38 (.12-.49)	.00 (.00-.20)	.62 (.51-.74)

Note: Scores were corrected for mean age and sex differences (see Methods).

Supplementary Table 46. For twins living together: T2 change twin intraclass correlations and model fitting results for univariate analyses of additive genetic (A), shared environmental (C), and non-shared environmental (E) components of variance (95% confidence intervals in parentheses).

**Construct**	**T2 change**											
	MZ DZ combined			MZ			DZ					
	r	*p*	*n*	*r*	*p*	*n*	*r*	*p*	*n*	A	C	E
Love and relationships	.09	.142	282	.20	.017	150	-.04	.678	129	.15 (.00-.29)	.00 (NA-.17)	.85 (.71-.99)
Achievement motivation	.09	.137	293	.17	.037	160	-.04	.680	130	.12 (.00-.26)	.00 (.00-.16)	.88 (.74-1.00)
Importance of Relationships	.05	.388	293	.18	.025	160	-.11	.218	130	.11 (NA-.24)	.00 (NA-.13)	.89 (.76-NA)

Purpose in Life	.16	.007	293	.14	.080	160	.17	.053	130	.01 (.00-.31)	.15 (.00-.26)	.85 (.69-.96)
Healthcare	.17	.003	292	.05	.573	159	.27	.002	130	.00 (NA-.25)	.18 (.00-.30)	.82 (.70-.94)
Community satisfaction	.20	<.001	294	.28	<.001	160	.08	.345	131	.26 (.00-.38)	.00 (.00-.28)	.74 (.62-.88)
Attitudes towards money	.17	.003	286	.30	<.001	156	.03	.738	127	.25 (.01-.38)	.00 (.00-.19)	.75 (.62-.89)
Alcohol (frequency x quantity)	.19	.003	254	.20	.022	135	.20	.035	117	.09 (NA-.38)	.13 (.00-.31)	.78 (.62-.93)
Conduct problems	.16	.008	288	.14	.087	156	.19	.032	129	.00 (NA-.30)	.16 (.00-.27)	.84 (.73-.96)
Emotional problems	.22	<.001	288	.32	<.001	156	.10	.256	129	.30 (.00-.42)	.00 (NA-.25)	.70 (.58-.84)
Hyperactivity	.16	.006	288	.21	.008	156	.10	.242	129	.22 (.00-.36)	.00 (NA-.25)	.78 (.64-.93)
Peer problems	.18	.003	288	.23	.004	156	.08	.378	129	.22 (.00-.35)	.00 (NA-.28)	.78 (.65-.92)
Prosocial behaviour	.13	.028	288	.23	.005	156	.04	.656	129	.19 (.00-.33)	.00 (NA-.19)	.81 (.67-.95)
General anxiety	.24	<.001	289	.38	<.001	154	.11	.215	132	.36 (.14-.49)	.00 (.00-.15)	.64 (.51-.78)
Depression	.21	<.001	288	.28	<.001	156	.11	.200	129	.27 (.00-.40)	.00 (.00-.25)	.73 (.60-.86)
Physical activity	.21	<.001	289	.22	.005	159	.18	.038	127	.12 (.00-.39)	.12 (.00-.32)	.75 (.61-.90)
Media use	.29	<.001	285	.39	<.001	158	.15	.089	124	.38 (.07-.49)	.00 (.00-.24)	.62 (.51-.75)
Volunteering	.18	.002	287	.25	.002	155	.12	.189	129	.25 (.00-.38)	.00 (NA-.23)	.75 (.62-.90)

Note: Scores were corrected for mean age and sex differences (see Methods).

Supplementary Table 47. For twins living apart: T1 twin intraclass correlations and model fitting results for univariate analyses of additive genetic (A), shared environmental (C), and non-shared environmental (E) components of variance (95% confidence intervals in parentheses).

**Construct**	**T1**											
	MZ DZ combined			MZ			DZ					
	r	p	n	r	p	n	r	p	n	A	C	E
Love and relationships	.13	<.001	810	.16	.001	482	.08	.032	736	0.16 (.00-.23)	.00 (.00-.15)	.84 (.77-.92)
Achievement motivation	.29	<.001	833	.32	<.001	483	.19	<.001	739	0.24 (.03-.39)	.08 (.00-.23)	.68 (.61-.76)
Importance of Relationships	.26	<.001	833	.44	<.001	483	.14	<.001	739	0.41 (.34-.47)	.00 (.00-.05)	.59 (.53-.66)
Purpose in Life	.34	<.001	836	.37	<.001	483	.23	<.001	739	0.32 (.12-.46)	.06 (.00-.21)	.61 (.54-.69)
Healthcare	.12	.001	836	.37	<.001	484	.17	<.001	737	0.37 (.22-.44)	.00 (.00-.11)	.63 (.56-.70)
Community satisfaction	.14	<.001	836	.41	<.001	480	.37	<.001	730	0.13 (.00-.31)	.30 (.16-.42)	.57 (.51-.65)
Attitudes towards money	.09	.012	836	.26	<.001	463	.03	.420	692	0.21 (.14-.28)	.00 (.00-.06)	.79 (.72-.86)
Alcohol (frequency x quantity)	.25	<.001	831	.34	<.001	184	.17	.003	324	0.35 (.14-.42)	.00 (.00-.15)	.65 (.58-.73)
Conduct problems	.15	<.001	831	.18	.006	222	.05	.302	389	0.28 (.07-.37)	.01 (.00-.17)	.70 (.63-.79)
Emotional problems	.24	<.001	831	.33	<.001	470	.16	<.001	726	0.33 (.12-.40)	.00 (.00-.15)	.67 (.60-.74)
Hyperactivity	.20	<.001	831	.33	<.001	470	.14	<.001	726	0.33 (.18-.40)	.00 (.00-.10)	.67 (.60-.74)

Peer problems	.29	<.001	831	.38	<.001	222	.20	<.001	389	0.20 (.00-.40)	.14 (.00-.29)	.66 (.58-.74)
Prosocial behaviour	.29	<.001	831	.24	<.001	222	.05	.341	389	0.29 (.10-.36)	.00 (NA-.14)	.71 (.64-.79)
General anxiety	.27	<.001	831	.34	<.001	446	.12	.003	666	0.32 (.24-.39)	.00 (.00-.08)	.68 (.61-.76)
Depression	.24	<.001	831	.37	<.001	222	.19	<.001	389	0.28 (.07-.39)	.03 (.00-.19)	.68 (.61-.77)
Physical activity	.31	<.001	831	.36	<.001	475	.17	<.001	723	0.36 (.29-.43)	.00 (.00-.14)	.64 (.57-.71)
Media use	.27	<.001	831	.42	<.001	475	.19	<.001	723	0.42 (.27-.48)	.00 (.00-.11)	.58 (.52-.65)
Volunteering	.20	<.001	831	.35	<.001	470	.14	<.001	726	0.32 (.18-.39)	.00 (.00-.11)	.68 (.61-.75)

Note: Scores were corrected for mean age and sex differences (see Methods).

Supplementary Table 48. For twins living apart: T2 twin intraclass correlations and model fitting results for univariate analyses of additive genetic (A), shared environmental (C), and non-shared environmental (E) components of variance (95% confidence intervals in parentheses).

**Construct**	**T2**											
	MZ DZ combined			MZ			DZ					
	r	p	n	r	p	n	r	p	n	A	C	E
Love and relationships	.13	<.001	810	.23	<.001	362	.05	.300	442	.19 (.03-.28)	.00 (.00-.12)	.81 (.72-.90)
Achievement motivation	.29	<.001	833	.40	<.001	367	.20	<.001	460	.37 (.13-.46)	.02 (.00-.20)	.61 (.54-.70)

Importance of Relationships	.26	<.001	833	.36	<.001	367	.18	<.001	460	.36 (.12-.43)	.00 (.00-.19)	.64 (.57-.72)
Purpose in Life	.34	<.001	836	.46	<.001	370	.25	<.001	460	.49 (.27-.55)	.00 (.00-.17)	.51 (.45-.59)
Healthcare	.12	.001	836	.22	<.001	370	.01	.757	460	.19 (.04-.27)	.00 (.00-.11)	.81 (.73-.90)
Community satisfaction	.14	<.001	836	.21	<.001	370	.06	.170	460	.20 (.03-.28)	.00 (.00-.11)	.80 (.72-.90)
Attitudes towards money	.09	.012	836	.11	.030	370	.06	.171	460	.11 (.00-.20)	.00 (.00-.13)	.89 (.80-.98)
Alcohol (frequency x quantity)	.25	<.001	831	.33	<.001	368	.18	<.001	457	.36 (.11-.45)	.01 (.00-.20)	.63 (.55-.73)
Conduct problems	.15	<.001	831	.16	.002	368	.14	.002	457	.00 (.00-.25)	.18 (.00-.25)	.82 (.75-.89)
Emotional problems	.24	<.001	831	.35	<.001	368	.17	<.001	457	.35 (.13-.42)	.00 (.00-.16)	.65 (.58-.73)
Hyperactivity	.20	<.001	831	.37	<.001	368	.06	.172	457	.33 (.23-.41)	.00 (.00-.06)	.67 (.59-.75)
Peer problems	.29	<.001	831	.39	<.001	368	.20	<.001	457	.33 (.08-.46)	.05 (.00-.24)	.62 (.54-.71)
Prosocial behaviour	.29	<.001	831	.33	<.001	368	.24	<.001	457	.19 (.00-.40)	.14 (.00-.31)	.67 (.59-.77)
General anxiety	.27	<.001	831	.37	<.001	368	.19	<.001	457	.39 (.19-.47)	.00 (.00-.15)	.61 (.53-.69)
Depression	.24	<.001	831	.35	<.001	368	.16	.001	457	.35 (.27-.43)	.00 (.00-.14)	.65 (.57-.73)
Physical activity	.31	<.001	831	.40	<.001	368	.24	<.001	457	.35 (.11-.49)	.07 (.00-.25)	.58 (.51-.67)
Media use	.27	<.001	831	.44	<.001	368	.09	.057	457	.38 (.28-.45)	.00 (.00-.06)	.62 (.55-.70)

Volunteering	.20	<.001	831	.37	<.001	368	.08	.082	457	.34 (.23-.42)	.00 (NA-.07)	.66 (.58-.75)

Note: Scores were corrected for mean age and sex differences (see Methods).

Supplementary Table 49. For twins living apart: T2 change twin intraclass correlations and model fitting results for univariate analyses of additive genetic (A), shared environmental (C), and non-shared environmental (E) components of variance (95% confidence intervals in parentheses).

**Construct**	**T2 change**											
	MZ DZ combined			MZ			DZ					
	r	p	n	r	p	n	r	p	n	A	C	E
Love and relationships	.10	.006	739	.15	.007	332	.06	.237	401	.13 (.00-.22)	.00 (.00-.16)	.87 (.78-.96)
Achievement motivation	.12	.001	752	.16	.004	332	.09	.066	414	.14 (.00-.25)	.02 (NA-.18)	.84 (.75-.94)
Importance of Relationships	.05	.146	752	.07	.228	332	.05	.320	414	.04 (.00-.17)	.03 (.00-.13)	.93 (.83-1.00)
Purpose in Life	.11	.003	755	.13	.015	335	.10	.050	414	.07 (.00-.23)	.06 (.00-.18)	.87 (.77-.96)
Healthcare	.06	.119	755	.13	.017	336	-.02	.767	413	.09 (.00-.19)	.00 (.00-.11)	.91 (.81-1.00)
Community satisfaction	.05	.165	761	.16	.003	339	-.05	.269	416	.10 (.00-.20)	.00 (NA-.07)	.90 (.80-1.00)
Attitudes towards money	.01	.770	731	.03	.601	332	-.01	.796	394	.01 (NA-.11)	.00 (.00-.07)	.99 (.89-1.00)

Alcohol (frequency x quantity)	.08	.037	667	.20	.001	307	-.02	.746	356	.18 (.04-.27)	.00 (.00-.09)	.82 (.73-.93)
Conduct problems	.11	.002	742	.14	.013	328	.10	.047	409	.08 (.00-.24)	.05 (.00-.18)	.86 (.76-.96)
Emotional problems	.12	.001	742	.21	<.001	328	.04	.378	409	.19 (.02-.28)	.00 (.00-.12)	.81 (.72-.91)
Hyperactivity	.14	<.001	742	.19	<.001	328	.09	.075	409	.19 (.00-.28)	.00 (.00-.18)	.81 (.72-.90)
Peer problems	.12	.001	742	.17	.002	328	.08	.118	409	.16 (NA-.25)	.00 (.00-.18)	.84 (.75-.94)
Prosocial behaviour	.14	<.001	742	.11	.054	328	.17	.001	409	.00 (.00-.18)	.14 (.00-.21)	.86 (.79-.93)
General anxiety	.13	<.001	708	.19	.001	321	.10	.051	381	.20 (.00-.29)	.00 (.00-.18)	.80 (.71-.90)
Depression	.07	.073	742	.06	.248	328	.07	.159	409	.00 (.00-.17)	.07 (.00-.14)	.93 (.86-1.00)
Physical activity	.21	<.001	751	.25	<.001	334	.17	<.001	411	.18 (.00-.36)	.08 (.00-.27)	.74 (.64-.84)
Media use	.14	<.001	751	.28	<.001	335	.00	.957	410	.21 (.13-.30)	.00 (.00-.09)	.79 (.70-.87)
Volunteering	.17	<.001	742	.34	<.001	328	.06	.245	409	.30 (.21-.39)	.00 (.00-.07)	.70 (.61-.79)

Note: Scores were corrected for mean age and sex differences (see Methods).

Supplementary Table 50. Male model fitting results for univariate analyses of additive genetic (A), shared environmental (C), and non-shared environmental (E) components of variance (95% confidence intervals in parentheses).

Construct	T1			T2			T2 change		
	A	C	E	A	C	E	A	C	E
Love and relationships	.19 (.00-.29)	.00 (.00-.16)	.81 (.71-.91)	.06 (.00-.28)	.07 (.00-.21)	.87 (.72-.99)	.00 (.00-.27)	.13 (.00-.24)	.87 (.73-.98)
Achievement motivation	.37 (.10-.46)	.01 (.00-.20)	.62 (.54-.73)	.33 (.00-.54)	.09 (.00-.36)	.58 (.46-.73)	.08 (.00-.26)	.02 (.00-.19)	.90 (.74-1.00)
Importance of Relationships	.41 (.30-.49)	.00 (.00-.05)	.59 (.51-.69)	.36 (.04-.47)	.00 (.00-.26)	.64 (.53-.76)	.16 (.00-.30)	.00 (.00-.00)	.84 (.70-.98)
Purpose in Life	.25 (.00-.47)	.14 (.00-.32)	.62 (.52-.72)	.13 (.00-.48)	.25 (.00-.42)	.62 (.50-.74)	.10 (.00-.25)	.01 (.00-.20)	.89 (.75-1.00)
Healthcare	.37 (.12-.45)	.00 (.00-.00)	.63 (.55-.73)	.00 (.00-.25)	.29 (.19-.37)	.71 (.63-.81)	.00 (.00-.24)	.20 (.00-.29)	.80 (.70-.89)
Community satisfaction	.14 (.00-.37)	.29 (.12-.43)	.57 (.48-.67)	.27 (.00-.39)	.00 (NA-.27)	.73 (.61-.89)	.08 (.00-.23)	.00 (.00-.14)	.92 (.77-1.00)
Attitudes towards money	.15 (.00-.25)	.00 (.00-.00)	.85 (.75-.94)	.19 (.00-.31)	.00 (.00-.13)	.81 (.69-.95)	.15 (.00-.29)	.00 (NA-NA)	.85 (.71-.99)
Alcohol (frequency x quantity)	.23 (.00-.42)	.09 (.00-.30)	.68 (.58-.79)	.20 (.00-.53)	.24 (.00-.45)	.56 (.45-.68)	.23 (.00-.44)	.07 (.00-.34)	.70 (.56-.87)
Conduct problems	.31 (.14-.39)	.00 (.00-.11)	.69 (.61-.79)	.00 (.00-.32)	.24 (.00-.34)	.76 (.66-.87)	.00 (.00-.24)	.10 (.00-.22)	.90 (.76-1.00)

Emotional problems	.12 (.00-.36)	.14 (.00-.28)	.74 (.64-.84)	.36 (.00-.48)	.00 (.00-.25)	.64 (.52-.80)	.00 (.00-.27)	.10 (.00-.21)	.90 (.73-1.00)
Hyperactivity	.32 (.09-.41)	.00 (.00-.16)	.68 (.59-.79)	.34 (.14-.46)	.00 (.00-.14)	.66 (.54-.79)	.23 (.00-.43)	.05 (.00-.30)	.71 (.57-.87)
Peer problems	.35 (.08-.46)	.02 (.00-.22)	.63 (.54-.74)	.09 (.00-.44)	.23 (.00-.39)	.68 (.55-.80)	.00 (.00-.23)	.12 (.00-.23)	.88 (.76-1.00)
Prosocial behaviour	.34 (.09-.42)	.00 (.00-.18)	.66 (.58-.76)	.45 (.19-.54)	.00 (.00-.19)	.55 (.46-.67)	.22 (.00-.36)	.00 (.00-.21)	.78 (.64-.94)
General anxiety	.34 (.09-.44)	.00 (.00-.17)	.66 (.56-.78)	.42 (.18-.54)	.00 (.00-.15)	.58 (.46-.73)	.03 (.00-.22)	.00 (.00-.13)	.97 (.78-1.00)
Depression	.25 (.00-.34)	.00 (.00-.19)	.75 (.66-.87)	.43 (.19-.55)	.00 (.00-.15)	.57 (.45-.72)	.30 (.00-.46)	.00 (.00-.19)	.70 (.54-.89)
Physical activity	.34 (.15-.43)	.00 (.00-.14)	.66 (.57-.75)	.32 (.00-.47)	.02 (NA-.31)	.65 (.53-.81)	.17 (.00-.33)	.02 (.00-.25)	.81 (.67-.97)
Media use	.52 (.38-.59)	.00 (.00-.10)	.48 (.41-.57)	.36 (.18-.47)	.00 (.00-.13)	.64 (.53-.76)	.19 (.00-.32)	.00 (.00-.19)	.81 (.68-.95)
Volunteering	.38 (.19-.46)	.00 (.00-.13)	.62 (.54-.72)	.47 (.27-.57)	.00 (.00-.14)	.53 (.43-.65)	.39 (.21-.51)	.00 (.00-.11)	.61 (.49-.75)

Note: Scores were corrected for mean age and sex differences (see Methods).

Supplementary Table 51. Female model fitting results for univariate analyses of additive genetic (A), shared environmental (C), and non-shared environmental (E) components of variance (95% confidence intervals in parentheses).

**Construct**	**T1**			**T2**			**T2 change**		
	**A**	**C**	**E**	**A**	**C**	**E**	**A**	**C**	**E**
Love and relationships	.18 (.03-.24)	.00 (.00-.11)	.82 (.76-.89)	.26 (.12-.34)	.00 (.00-.09)	.74 (.66-.83)	.15 (.00-.24)	.00 (.00-.11)	.85 (.76-.94)

Achievement motivation	.34 (.24-.40)	.00 (.00-.07)	.66 (.60-.72)	.36 (.28-.43)	.00 (.00-.11)	.64 (.57-.72)	.17 (.00-.26)	.00 (.00-.16)	.83 (.74-.92)
Importance of Relationships	.35 (.28-.41)	.00 (.00-.00)	.65 (.59-.71)	.34 (.17-.41)	.00 (.00-.13)	.66 (.59-.73)	.05 (.00-.14)	.00 (.00-.10)	.95 (.86-1.00)
Purpose in Life	.39 (.25-.45)	.00 (.00-.11)	.61 (.55-.67)	.49 (.42-.55)	.00 (.00-.15)	.51 (.45-.58)	.04 (.00-.25)	.11 (.00-.21)	.85 (.75-.93)
Healthcare	.34 (.23-.40)	.00 (.00-.00)	.66 (.60-.72)	.12 (.00-.23)	.00 (.00-.14)	.88 (.77-1.00)	.00 (.00-.11)	.00 (.00-.07)	1.00 (.89-1.00)
Community satisfaction	.14 (.00-.30)	.28 (.15-.40)	.58 (.52-.64)	.30 (.15-.38)	.00 (.00-.11)	.70 (.62-.79)	.17 (.05-.26)	.00 (.00-.08)	.83 (.74-.93)
Attitudes towards money	.26 (.17-.32)	.00 (.00-.05)	.74 (.68-.81)	.15 (.00-.23)	.00 (.00-.15)	.85 (.77-.94)	.06 (.00-.16)	.00 (.00-.11)	.94 (.84-1.00)
Alcohol (frequency x quantity)	.36 (.26.42)	.00 (.00.00)	.64 (.58.71)	.39 (.23-.47)	.00 (.00-.12)	.61 (.53-.69)	.14 (.00-.24)	.00 (.00-.09)	.86 (.76-.96)
Conduct problems	.26 (.08-.32)	.00 (.00-.13)	.74 (.68-.82)	.08 (.00-.29)	.11 (.00-.24)	.81 (.71-.89)	.12 (.00-.27)	.06 (.00-.21)	.83 (.73-.93)
Emotional problems	.38 (.24-.44)	.00 (.00-.11)	.62 (.56-.68)	.40 (.17-.47)	.01 (.00-.19)	.59 (.53-.67)	.25 (.13-.34)	.00 (.00-.09)	.75 (.66-.84)
Hyperactivity	.40 (.29-.46)	.00 (.00-.08)	.60 (.54-.66)	.34 (.22-.41)	.00 (.00-.08)	.66 (.59-.75)	.16 (.00-.25)	.00 (.00-.15)	.84 (.75-.93)
Peer problems	.27 (.09-.44)	.13 (.00-.26)	.61 (.55-.67)	.46 (.23-.52)	.00 (.00-.19)	.54 (.48-.61)	.21 (.03-.29)	.00 (.00-.14)	.79 (.71-.88)

Prosocial behaviour	.22 (.08-.28)	.00 (.00-.10)	.78 (.72-.84)	.24 (.00-.38)	.06 (.00-.26)	.70 (.62-.79)	.00 (.00-.19)	.14 (.00-.21)	.86 (.79-.93)
General anxiety	.37 (.27.43)	.00 (.00.08)	.63 (.57.69)	.45 (.37-.52)	.00 (.00-.15)	.55 (.48-.63)	.31 (.11-.39)	.00 (.00-.15)	.69 (.61-.78)
Depression	.27 (.09-.42)	.09 (.00-.24)	.64 (.58-.70)	.39 (.16-.46)	.00 (.00-.18)	.61 (.54-.69)	.05 (.00-.20)	.05 (.00-.16)	.89 (.80-.98)
Physical activity	.42 (.27-.48)	.00 (.00-.12)	.58 (.52-.64)	.27 (.04-.49)	.17 (.00-.35)	.56 (.49-.64)	.17 (.00-.38)	.13 (.00-.30)	.71 (.61-.81)
Media use	.42 (.26-.48)	.00 (.00-.13)	.58 (.52-.64)	.47 (.33-.53)	.00 (.00-.11)	.53 (.47-.60)	.30 (.16-.37)	.00 (.00-.10)	.70 (.63-.79)
Volunteering	.30 (.19-.35)	.00 (.00-.00)	.70 (.65-.77)	.30 (.18-.38)	.00 (.00-.07)	.70 (.62-.79)	.24 (.14-.33)	.00 (.00-.10)	.76 (.67-.86)

Note: Scores were corrected for mean age and sex differences (see Methods).

Supplementary Table 52. Model fitting results for univariate analyses of additive genetic (A), shared environmental (C), and non-shared environmental (E) components of variance (95% confidence intervals in parentheses) for variables corrected for variation in SES.

**Construct**	**T1**			**T2**			**T2 change**		
	**A**	**C**	**E**	**A**	**C**	**E**	**A**	**C**	**E**
Love and relationships	.18 (.06-.24)	.00 (.00-.09)	.82 (.76-.88)	.23 (.06-.31)	.00 (.00-.13)	.77 (.69-.84)	.16 (.00-.24)	.00 (.00-.15)	.84 (.76-.90)
Achievement motivation	.37 (.26-.42)	.00 (.00-.07)	.63 (.58-.69)	.38 (.31-.45)	.00 (.00-.14)	.62 (.55-.69)	.14 (.00-.22)	.00 (.00-.14)	.86 (.78-.95)
Importance of Relationships	.39 (.34-.44)	.00 (.00-.00)	.61 (.56-.66)	.36 (.21-.42)	.00 (.00-.12)	.64 (.58-.71)	.09 (.00-.18)	.00 (.00-.10)	.91 (.82-.99)

Purpose in Life	.36 (.21-.45)	.04 (.00-.15)	.61 (.55-.66)	.44 (.25-.54)	.04 (.00-.19)	.52 (.46-.59)	.08 (.00-.25)	.09 (.00-.20)	.84 (.75-.92)
Healthcare	.36 (.27-.42)	.00 (.00-.06)	.64 (.58-.69)	.16 (.00-.30)	.06 (.00-.22)	.78 (.70-.87)	.08 (.00-.20)	.03 (.00-.15)	.89 (.80-.97)
Community satisfaction	.13 (.00-.27)	.28 (.18-.39)	.59 (.53-.64)	.29 (.16-.36)	.00 (.00-.09)	.71 (.64-.79)	.14 (.04-.22)	.00 (.00-.06)	.86 (.78-.95)
Attitudes towards money	.22 (.15-.27)	.00 (.00-.04)	.78 (.73-.84)	.15 (.03-.23)	.00 (.00-.09)	.85 (.77-.92)	.08 (.00-.16)	.00 (.00-.09)	.92 (.84-1.00)
Alcohol (frequency x quantity)	.36 (.23-.41)	.00 (.00-.09)	.64 (.59-.70)	.43 (.23-.49)	.00 (.00-.15)	.57 (.51-.64)	.21 (.07-.29)	.00 (.00-.09)	.79 (.71-.88)
Conduct problems	.23 (.11-.29)	.00 (.00-.09)	.77 (.71-.83)	.09 (.00-.30)	.13 (.00-.25)	.78 (.69-.86)	.05 (.00-.24)	.10 (.00-.20)	.85 (.76-.93)
Emotional problems	.33 (.18-.39)	.01 (.00-.13)	.66 (.61-.72)	.38 (.25-.45)	.00 (.00-.12)	.62 (.55-.68)	.23 (.11-.31)	.00 (.00-.09)	.77 (.69-.85)
Hyperactivity	.35 (.26-.41)	.00 (.00-.07)	.65 (.59-.70)	.34 (.26-.41)	.00 (.00-.05)	.66 (.59-.74)	.20 (.02-.28)	.00 (.00-.13)	.80 (.72-.88)
Peer problems	.30 (.15-.41)	.05 (.00-.17)	.64 (.59-.70)	.40 (.19-.47)	.00 (NA-.17)	.60 (.53-.67)	.14 (.00-.24)	.02 (.00-.18)	.84 (.76-.93)
Prosocial behaviour	.26 (.15-.32)	.00 (.00-.08)	.74 (.68-.79)	.36 (.29-.42)	.00 (.00-.14)	.64 (.58-.71)	.06 (.00-.24)	.10 (.00-.20)	.85 (.76-.92)
General anxiety	.37 (.27-.42)	.00 (.00-.07)	.63 (.58-.69)	.44 (.37-.50)	.00 (.00-.08)	.56 (.50-.63)	.25 (.10-.34)	.00 (.00-.08)	.75 (.66-.83)
Depression	.27 (.11-.37)	.04 (.00-.17)	.68 (.63-.74)	.39 (.28-.46)	.00 (.00-.08)	.61 (.54-.68)	.17 (.05-.25)	.00 (.00-.15)	.83 (.75-.91)

Physical activity	.38 (.26-.43)	.00 (.00-.09)	.62 (.57-.67)	.26 (.06-.46)	.13 (.00-.29)	.60 (.53-.68)	.13 (.00-.33)	.12 (.00-.27)	.75 (.67-.84)
Media use	.47 (.40-.51)	.00 (.00-.04)	.53 (.49-.58)	.45 (.38-.50)	.00 (.00-.07)	.55 (.50-.62)	.29 (.18-.36)	.00 (.00-.08)	.71 (.64-.78)
Volunteering	.32 (.24-.37)	.00 (.00-.00)	.68 (.63-.73)	.36 (.29-.43)	.00 (.00-.05)	.64 (.57-.71)	.29 (.19-.37)	.00 (.00-.06)	.71 (.63-.79)

Note: Scores were corrected for mean age and sex differences (see Methods).

Supplementary Table 53. Model fitting results for univariate analyses of additive genetic (A), shared environmental (C), and non-shared environmental (E) components of variance (95% confidence intervals in parentheses) for variables corrected for variation in job/financial difficulties.

**Construct**	**T2**			**T2 change**		
	**A**	**C**	**E**	**A**	**C**	**E**
Love and relationships	.22 (.08-.29)	.00 (.00-.08)	.78 (.71-.86)	.15 (.00-.23)	.00 (.00-.13)	.85 (.77-.93)
Achievement motivation	.40 (.23-.46)	.00 (.00-.13)	.60 (.54-.67)	.16 (.00-.23)	.00 (.00-.14)	.84 (.77-.93)
Importance of Relationships	.36 (.21-.42)	.00 (.00-.12)	.64 (.58-.71)	.09 (.00-.17)	.00 (.00-.10)	.91 (.83-.99)
Purpose in Life	.41 (.21-.52)	.05 (.00-.21)	.54 (.48-.61)	.11 (.00-.24)	.05 (.00-.18)	.84 (.76-.93)
Healthcare	.17 (.00-.32)	.06 (.00-.23)	.76 (.68-.86)	.11 (.00-.22)	.02 (.00-.16)	.87 (.78-.96)
Community satisfaction	.28 (.20-.35)	.00 (.00-.10)	.72 (.65-.80)	.14 (.04-.22)	.00 (.00-.00)	.86 (.78-.95)
Attitudes towards money	.15 (.02-.22)	.00 (.00-.10)	.85 (.78-.92)	.07 (.00-.16)	.00 (.00-.09)	.93 (.84-1.00)

Alcohol (frequency x quantity)	.43 (.23-.49)	.00 (.00-.15)	.57 (.51-.64)	.20 (.08-.29)	.00 (.00-.00)	.80 (.71-.89)
Conduct problems	.00 (.00-.21)	.19 (.02-.24)	.81 (.76-.88)	.04 (.00-.24)	.12 (.00-.21)	.84 (.76-.92)
Emotional problems	.39 (.22-.45)	.00 (.00-.13)	.61 (.55-.68)	.23 (.10-.30)	.00 (.00-.09)	.77 (.70-.85)
Hyperactivity	.33 (.24-.40)	.00 (.00-.06)	.67 (.60-.74)	.21 (.01-.29)	.00 (.00-.00)	.79 (.71-.87)
Peer problems	.37 (.17-.47)	.05 (.00-.21)	.59 (.53-.66)	.19 (.00-.26)	.00 (.00-.00)	.81 (.74-.90)
Prosocial behaviour	.34 (.13-.42)	.02 (.00-.18)	.64 (.58-.72)	.06 (.00-.26)	.11 (.00-.21)	.83 (.74-.91)
General anxiety	.43 (.29-.49)	.00 (.00-.10)	.57 (.51-.64)	.26 (.12-.34)	.00 (.00-.00)	.74 (.66-.82)
Depression	.38 (.24-.44)	.00 (.00-.11)	.62 (.56-.69)	.13 (.00-.23)	.02 (.00-.17)	.85 (.77-.94)
Physical activity	.30 (.10-.48)	.12 (.00-.27)	.58 (.52-.65)	.14 (.00-.34)	.12 (.00-.27)	.74 (.66-.83)
Media use	.43 (.37-.49)	.00 (.00-.07)	.57 (.51-.63)	.26 (.15-.33)	.00 (.00-.08)	.74 (.67-.81)
Volunteering	.34 (.26-.40)	.00 (.00-.06)	.66 (.60-.74)	.28 (.18-.35)	.00 (.00-.06)	.72 (.65-.80)

Note: Scores were corrected for mean age and sex differences (see Methods).

Supplementary Table 54. Model fitting results for univariate analyses of additive genetic (A), shared environmental (C), and non-shared environmental (E) components of variance (95% confidence intervals in parentheses) for variables corrected for variation in garden access during the lockdown.

**Construct**	**T2**			**T2 change**		
	**A**	**C**	**E**	**A**	**C**	**E**
Love and relationships	.23 (.09-.30)	.00 (.00-.00)	.77 (.70-.84)	.15 (.00-.23)	.00 (NA-NA)	.85 (.77-.92)

Achievement motivation	.38 (.22-.44)	.00 (.00-.13)	.62 (.56-.68)	.15 (.00-.23)	.00 (.00-.14)	.85 (.77-.93)
Importance of Relationships	.36 (.22-.42)	.00 (.00-.11)	.64 (.58-.71)	.10 (.00-.17)	.00 (.00-.00)	.90 (.83-.98)
Purpose in Life	.41 (.22-.52)	.05 (.00-.20)	.54 (.48-.61)	.05 (.00-.23)	.09 (.00-.18)	.86 (.77-.94)
Healthcare	.14 (.00-.30)	.07 (.00-.23)	.79 (.70-.87)	.08 (.00-.20)	.03 (.00-.15)	.89 (.80-.97)
Community satisfaction	.28 (.14-.35)	.00 (.00-.10)	.72 (.65-.79)	.14 (.04-.22)	.00 (.00-.00)	.86 (.78-.94)
Attitudes towards money	.16 (.03-.23)	.00 (.00-.09)	.84 (.77-.91)	.09 (.00-.17)	.00 (.00-.09)	.91 (.83-.99)
Alcohol (frequency x quantity)	.40 (.20-.49)	.02 (.00-.18)	.58 (.51-.65)	.19 (.06-.28)	.00 (.00-.00)	.81 (.72-.89)
Conduct problems	.01 (.00-.24)	.19 (.01-.25)	.81 (.72-.87)	.04 (.00-.23)	.10 (.00-.19)	.86 (.77-.93)
Emotional problems	.39 (.21-.45)	.00 (.00-.15)	.61 (.55-.68)	.22 (.10-.30)	.00 (.00-.00)	.78 (.70-.85)
Hyperactivity	.34 (.25-.40)	.00 (.00-.05)	.66 (.60-.73)	.20 (.01-.28)	.00 (.00-.15)	.80 (.72-.88)
Peer problems	.35 (.16-.48)	.07 (.00-.23)	.58 (.52-.65)	.18 (.00-.26)	.00 (.00-.18)	.82 (.74-.90)
Prosocial behaviour	.35 (.14-.42)	.01 (.00-.17)	.65 (.58-.72)	.03 (.00-.23)	.12 (.00-.20)	.85 (.77-.92)
General anxiety	.44 (.32-.50)	.00 (.00-.09)	.56 (.50-.63)	.26 (.11-.33)	.00 (.00-.00)	.74 (.67-.82)
Depression	.39 (.24-.45)	.00 (.00-.11)	.61 (.55-.68)	.12 (.00-.22)	.02 (.00-.16)	.86 (.78-.95)
Physical activity	.30 (.10-.47)	.12 (.00-.27)	.58 (.52-.66)	.17 (.00-.34)	.10 (.00-.26)	.74 (.66-.83)
Media use	.44 (.35-.49)	.00 (.00-.06)	.56 (.51-.62)	.27 (.16-.33)	.00 (.00-.07)	.73 (.67-.81)
Volunteering	.35 (.26-.41)	.00 (.00-.05)	.65 (.59-.72)	.28 (.19-.36)	.00 (.00-.00)	.72 (.64-.80)

Note: Scores were corrected for mean age and sex differences (see Methods).

Supplementary Table 55. Model fitting results for univariate analyses of additive genetic (A), shared environmental (C), and non-shared environmental (E) components of variance (95% confidence intervals in parentheses) for variables corrected for variation in living conditions (number of rooms: people in home during the lockdown).

Construct	T2			T2 change		
	A	C	E	A	C	E
Love and relationships	.23 (.09-.30)	.00 (.00-.10)	.77 (.70-.85)	.15 (.00-.23)	.00 (.00-.13)	.85 (.77-.92)
Achievement motivation	.38 (.21-.44)	.00 (.00-.13)	.62 (.56-.69)	.15 (.00-.23)	.00 (.00-.14)	.85 (.77-.93)
Importance of Relationships	.36 (.21-.42)	.00 (.00-.11)	.64 (.58-.71)	.09 (.00-.17)	.00 (.00-.00)	.91 (.83-.99)
Purpose in Life	.40 (.21-.52)	.06 (.00-.21)	.54 (.48-.61)	.05 (.00-.23)	.09 (.00-.19)	.86 (.77-.93)
Healthcare	.15 (.00-.29)	.06 (.00-.21)	.79 (.71-.88)	.08 (.00-.20)	.03 (.00-.15)	.89 (.80-.97)
Community satisfaction	.29 (.15-.36)	.00 (.00-.10)	.71 (.64-.78)	.14 (.04-.22)	.00 (.00-.06)	.86 (.78-.94)
Attitudes towards money	.16 (.03-.23)	.00 (.00-.09)	.84 (.77-.91)	.09 (.00-.17)	.00 (.00-.09)	.91 (.83-.99)
Alcohol (frequency x quantity)	.40 (.20-.49)	.02 (.00-.18)	.58 (.51-.65)	.20 (.07-.28)	.00 (.00-.00)	.80 (.72-.88)
Conduct problems	.00 (.00-.23)	.18 (.01-.24)	.82 (.74-.88)	.04 (.00-.23)	.10 (.00-.19)	.86 (.77-.93)
Emotional problems	.39 (.19-.45)	.00 (.00-.16)	.61 (.55-.68)	.22 (.10-.30)	.00 (.00-.09)	.78 (.70-.86)
Hyperactivity	.34 (.25-.41)	.00 (.00-.06)	.66 (.59-.73)	.20 (.00-.28)	.00 (.00-.15)	.80 (.72-.88)
Peer problems	.36 (.17-.48)	.06 (.00-.22)	.58 (.52-.65)	.18 (.00-.25)	.00 (.00-.17)	.82 (.75-.90)
Prosocial behaviour	.34 (.14-.42)	.01 (.00-.17)	.64 (.58-.72)	.02 (.00-.22)	.11 (.00-.19)	.86 (.78-.93)

General anxiety	.43 (.30-.49)	.00 (.00-.10)	.57 (.51-.63)	.25 (.10-.33)	.00 (.00-.00)	.75 (.67-.83)
Depression	.39 (.23-.45)	.00 (.00-.12)	.61 (.55-.68)	.12 (.00-.22)	.02 (.00-.16)	.86 (.78-.94)
Physical activity	.30 (.11-.48)	.11 (.00-.27)	.58 (.52-.65)	.18 (.00-.34)	.09 (.00-.25)	.74 (.66-.83)
Media use	.44 (.36-.50)	.00 (.00-.06)	.56 (.50-.62)	.27 (.16-.34)	.00 (.00-.08)	.73 (.66-.80)
Volunteering	.35 (.26-.42)	.00 (.00-.00)	.65 (.58-.72)	.29 (.19-.36)	.00 (.00-.06)	.71 (.64-.79)

Note: Scores were corrected for mean age and sex differences (see Methods).

Supplementary Table 56. Model fitting results for univariate analyses of additive genetic (A), shared environmental (C), and non-shared environmental (E) components of variance (95% confidence intervals in parentheses) for variables corrected for variation in having been tested or suspected of having COVID-19.

**Construct**	**T2**			**T2 change**		
	**A**	**C**	**E**	**A**	**C**	**E**
Love and relationships	.23 (.09-.30)	.00 (.00-.10)	.77 (.70-.85)	.15 (.00-.23)	.00 (.00-.00)	.85 (.77-.92)
Achievement motivation	.38 (.22-.44)	.00 (.00-.12)	.62 (.56-.68)	.15 (.00-.23)	.00 (.00-.00)	.85 (.77-.93)
Importance of Relationships	.35 (.22-.41)	.00 (.00-.10)	.65 (.59-.71)	.09 (.00-.17)	.00 (.00-.10)	.91 (.83-.99)
Purpose in Life	.41 (.22-.52)	.05 (.00-.20)	.54 (.48-.60)	.06 (.00-.23)	.08 (.00-.19)	.86 (.77-.93)
Healthcare	.14 (.00-.30)	.08 (.00-.23)	.78 (.70-.87)	.08 (.00-.20)	.03 (.00-.15)	.89 (.80-.97)
Community satisfaction	.30 (.15-.36)	.00 (.00-.00)	.70 (.64-.78)	.15 (.05-.23)	.00 (.00-.06)	.85 (.77-.93)

Attitudes towards money	.16 (.03-.23)	.00 (.00-.10)	.84 (.77-.91)	.09 (.00-.17)	.00 (.00-.09)	.91 (.83-.99)
Alcohol (frequency x quantity)	.40 (.20-.49)	.03 (.00-.19)	.57 (.51-.65)	.20 (.06-.28)	.00 (.00-.09)	.80 (.72-.89)
Conduct problems	.00 (.00-.22)	.20 (.02-.25)	.80 (.72-.86)	.03 (.00-.23)	.11 (.00-.20)	.86 (.77-.93)
Emotional problems	.38 (.20-.44)	.00 (.00-.14)	.62 (.56-.68)	.21 (.09-.29)	.00 (.00-.08)	.79 (.71-.86)
Hyperactivity	.33 (.24-.40)	.00 (.00-.06)	.67 (.60-.74)	.20 (.00-.27)	.00 (.00-.16)	.80 (.73-.89)
Peer problems	.36 (.17-.48)	.06 (.00-.22)	.58 (.52-.65)	.18 (.00-.26)	.00 (.00-.00)	.82 (.74-.90)
Prosocial behaviour	.34 (.14-.42)	.01 (.00-.17)	.64 (.58-.72)	.03 (.00-.23)	.12 (.00-.20)	.85 (.77-.92)
General anxiety	.43 (.31-.49)	.00 (.00-.09)	.57 (.51-.64)	.25 (.10-.33)	.00 (.00-.11)	.75 (.67-.83)
Depression	.38 (.23-.45)	.00 (.00-.12)	.62 (.55-.69)	.10 (.00-.21)	.03 (.00-.16)	.87 (.79-.95)
Physical activity	.30 (.10-.47)	.12 (.00-.27)	.58 (.52-.66)	.16 (.00-.34)	.10 (.00-.26)	.74 (.66-.83)
Media use	.44 (.35-.49)	.00 (.00-.06)	.56 (.51-.62)	.26 (.16-.33)	.00 (.00-.08)	.74 (.67-.81)
Volunteering	.35 (.26-.41)	.00 (.00-.05)	.65 (.59-.72)	.28 (.18-.36)	.00 (.00-.06)	.72 (.64-.80)

Note: Scores were corrected for mean age and sex differences (see Methods).

Supplementary Table 57. Model fitting results for univariate analyses of additive genetic (A), shared environmental (C), and non-shared environmental (E) components of variance for variables (95% confidence intervals in parentheses) corrected for variation in number of COVID-19 symptoms.

**Construct**	**T2**			**T2 change**		
	**A**	**C**	**E**	**A**	**C**	**E**

Love and relationships	.23 (.08-.30)	.00 (.00-.10)	.77 (.70-.85)	.15 (.00-.23)	.00 (.00-.00)	.85 (.77-.93)
Achievement motivation	.38 (.22-.44)	.00 (.00-.00)	.62 (.56-.68)	.15 (.00-.23)	.00 (.00-.14)	.85 (.77-.94)
Importance of Relationships	.35 (.22-.41)	.00 (.00-.10)	.65 (.59-.71)	.09 (.00-.17)	.00 (.00-.00)	.91 (.83-.99)
Purpose in Life	.38 (.19-.51)	.07 (.00-.22)	.55 (.49-.62)	.03 (.00-.22)	.10 (.00-.18)	.87 (.78-.94)
Healthcare	.14 (.00-.30)	.08 (.00-.23)	.78 (.70-.87)	.09 (.00-.20)	.03 (.00-.15)	.88 (.80-.97)
Community satisfaction	.29 (.15-.36)	.00 (.00-.00)	.71 (.64-.78)	.14 (.05-.22)	.00 (.00-.00)	.86 (.78-.94)
Attitudes towards money	.16 (.03-.23)	.00 (.00-.00)	.84 (.77-.91)	.09 (.00-.17)	.00 (.00-.09)	.91 (.83-.99)
Alcohol (frequency x quantity)	.41 (.21-.49)	.02 (.00-.18)	.57 (.51-.65)	.20 (.06-.28)	.00 (.00-.00)	.80 (.72-.89)
Conduct problems	.00 (.00-.23)	.19 (.01-.25)	.81 (.72-.87)	.04 (.00-.23)	.10 (.00-.19)	.86 (.77-.93)
Emotional problems	.37 (.17-.43)	.00 (.00-.16)	.63 (.57-.71)	.20 (.07-.28)	.00 (.00-.09)	.80 (.72-.88)
Hyperactivity	.33 (.23-.39)	.00 (.00-.06)	.67 (.61-.74)	.20 (.00-.27)	.00 (.00-.16)	.80 (.73-.89)
Peer problems	.35 (.15-.48)	.07 (.00-.23)	.58 (.52-.65)	.18 (.00-.25)	.00 (.00-.18)	.82 (.75-.91)
Prosocial behaviour	.34 (.14-.42)	.01 (.00-.17)	.64 (.58-.72)	.03 (.00-.23)	.12 (.00-.20)	.85 (.77-.92)
General anxiety	.41 (.27-.47)	.00 (.00-.11)	.59 (.53-.66)	.25 (.08-.32)	.00 (.00-.12)	.75 (.68-.84)
Depression	.37 (.21-.43)	.00 (.00-.00)	.63 (.57-.70)	.09 (.00-.20)	.03 (.00-.16)	.88 (.80-.96)
Physical activity	.30 (.10-.47)	.12 (.00-.27)	.58 (.52-.66)	.17 (.00-.34)	.10 (.00-.26)	.74 (.66-.83)
Media use	.44 (.35-.49)	.00 (.00-.06)	.56 (.51-.62)	.26 (.16-.33)	.00 (.00-.08)	.74 (.67-.81)

Volunteering	.35 (.26-.41)	.00 (.00-.00)	.65 (.59-.72)	.28 (.19-.36)	.00 (.00-.06)	.72 (.64-.79)

Note: Scores were corrected for mean age and sex differences (see Methods).

Supplementary Table 58. Model fitting results for univariate analyses of additive genetic (A), shared environmental (C), and non-shared environmental (E) components of variance for variables (95% confidence intervals in parentheses) for variables corrected for variation in number of COVID-19 symptoms (two or more) vs none.

**Construct**	**T2**			**T2 change**		
	**A**	**C**	**E**	**A**	**C**	**E**
Love and relationships	.27 (.10-.35)	.00 (.00-.13)	.73 (.65-.81)	.22 (.00-.30)	.00 (.00-.18)	.78 (.70-.87)
Achievement motivation	.31 (.07-.45)	.06 (.00-.25)	.63 (.55-.71)	.04 (.00-.21)	.07 (.00-.17)	.89 (.79-.97)
Importance of Relationships	.35 (.16-.42)	.00 (.00-.16)	.65 (.58-.72)	.07 (.00-.15)	.00 (.00-.10)	.93 (.85-1.00)
Purpose in Life	.29 (.07-.50)	.15 (.00-.32)	.56 (.49-.64)	.00 (.00-.19)	.14 (.00-.21)	.86 (.78-.93)
Healthcare	.08 (.00-.24)	.07 (.00-.19)	.86 (.76-.95)	.00 (.00-.00)	.05 (.00-.12)	.95 (.85-1.00)
Community satisfaction	.3 (.05-.37)	.00 (.00-.19)	.7 (.63-.79)	.15 (.01-.24)	.00 (.00-.10)	.85 (.76-.94)
Attitudes towards money	.19 (.02-.27)	.00 (.00-.13)	.81 (.73-.89)	.11 (.00-.20)	.00 (.00-.00)	.89 (.80-.98)
Alcohol (frequency x quantity)	.28 (.05-.47)	.11 (.00-.29)	.61 (.53-.69)	.19 (.00-.29)	.00 (.00-.17)	.81 (.71-.9)
Conduct problems	.08 (.00-.28)	.10 (.00-.22)	.82 (.72-.92)	.12 (.00-.25)	.03 (.00-.18)	.85 (.75-.96)

Emotional problems	.22 (.00-.39)	.09 (.00-.28)	.69 (.61-.78)	.18 (.00-.26)	.00 (.00-.17)	.82 (.74-.92)
Hyperactivity	.33 (.19-.40)	.00 (.00-.10)	.67 (.60-.76)	.13 (.00-.28)	.06 (.00-.22)	.81 (.72-.90)
Peer problems	.39 (.17-.50)	.03 (.00-.22)	.57 (.50-.65)	.15 (.00-.24)	.01 (.00-.18)	.84 (.76-.94)
Prosocial behaviour	.37 (.13-.44)	.00 (.00-.19)	.63 (.56-.72)	.13 (.00-.28)	.06 (.00-.21)	.82 (.72-.92)
General anxiety	.38 (.19-.46)	.00 (.00-.15)	.62 (.54-.70)	.27 (.05-.36)	.00 (.00-.16)	.73 (.64-.82)
Depression	.31 (.07-.42)	.03 (.00-.21)	.66 (.58-.75)	.00 (.00-.19)	.10 (.00-.17)	.90 (.81-.97)
Physical activity	.26 (.03-.48)	.16 (.00-.34)	.58 (.51-.67)	.08 (.00-.33)	.16 (.00-.29)	.76 (.66-.85)
Media use	.45 (.33-.52)	.00 (.00-.10)	.55 (.48-.62)	.29 (.13-.36)	.00 (.00-.00)	.71 (.64-.80)
Volunteering	.33 (.21-.40)	.00 (.00-.08)	.67 (.60-.75)	.28 (.15-.36)	.00 (.00-.00)	.72 (.64-.81)

Note: Scores were corrected for mean age and sex differences (see Methods).

Supplementary Table 59. Model fitting results for univariate analyses of additive genetic (A), shared environmental (C), and non-shared environmental (E) components of variance for variables (95% confidence intervals in parentheses) for variables corrected for impact of COVID-19 on family health.

**Construct**	**T2**			**T2 change**		
	**A**	**C**	**E**	**A**	**C**	**E**
Love and relationships	.23 (.10-.30)	.00 (.00-.09)	.77 (.70-.85)	.15 (.00-.23)	.00 (.00-.00)	.85 (.77-.92)
Achievement motivation	.38 (.20-.44)	.00 (.00-.00)	.62 (.56-.69)	.15 (.00-.23)	.00 (.00-.14)	.85 (.77-.93)
Importance of Relationships	.36 (.21-.41)	.00 (.00-.11)	.64 (.59-.71)	.09 (.00-.17)	.00 (.00-.00)	.91 (.83-.99)

Purpose in Life	.43 (.24-.52)	.03 (.00-.18)	.54 (.48-.61)	.05 (.00-.23)	.09 (.00-.18)	.86 (.77-.93)
Healthcare	.13 (.00-.28)	.07 (.00-.22)	.80 (.72-.88)	.08 (.00-.20)	.03 (.00-.15)	.89 (.80-.97)
Community satisfaction	.29 (.15-.36)	.00 (.00-.11)	.71 (.64-.78)	.14 (.05-.22)	.00 (.00-.06)	.86 (.78-.94)
Attitudes towards money	.17 (.03-.24)	.00 (.00-.10)	.83 (.76-.90)	.09 (.00-.17)	.00 (.00-.09)	.91 (.83-.99)
Alcohol (frequency x quantity)	.41 (.20-.48)	.01 (.00-.17)	.58 (.52-.66)	.18 (.03-.26)	.00 (NA-NA)	.82 (.74-.91)
Conduct problems	.06 (.00-.27)	.13 (.00-.23)	.81 (.73-.89)	.04 (.00-.23)	.10 (.00-.19)	.86 (.77-.93)
Emotional problems	.39 (.21-.45)	.00 (.00-.14)	.61 (.55-.68)	.22 (.09-.30)	.00 (.00-.09)	.78 (.70-.86)
Hyperactivity	.34 (.25-.40)	.00 (.00-.00)	.66 (.60-.73)	.20 (.00-.28)	.00 (.00-.15)	.80 (.72-.88)
Peer problems	.38 (.19-.49)	.05 (.00-.21)	.57 (.51-.64)	.18 (.00-.26)	.00 (.00-.18)	.82 (.74-.90)
Prosocial behaviour	.34 (.13-.43)	.02 (.00-.18)	.64 (.57-.71)	.03 (.00-.23)	.12 (.00-.20)	.85 (.77-.92)
General anxiety	.44 (.30-.50)	.00 (.00-.11)	.56 (.50-.63)	.26 (.10-.34)	.00 (.00-.11)	.74 (.66-.82)
Depression	.39 (.24-.45)	.00 (.00-.00)	.61 (.55-.68)	.11 (.00-.22)	.03 (.00-.16)	.86 (.78-.95)
Physical activity	.29 (.10-.47)	.12 (.00-.28)	.58 (.52-.66)	.17 (.00-.34)	.09 (.00-.26)	.74 (.66-.83)
Media use	.44 (.35-.50)	.00 (.00-.07)	.56 (.50-.62)	.26 (.16-.33)	.00 (.00-.08)	.74 (.67-.81)
Volunteering	.35 (.26-.41)	.00 (.00-.05)	.65 (.59-.72)	.28 (.19-.36)	.00 (.00-.06)	.72 (.64-.79)

Note: Scores were corrected for mean age and sex differences (see Methods).

Supplementary Table 60. Model fitting results for univariate analyses of additive genetic (A), shared environmental (C), and non-shared environmental (E) components of variance for variables (95% confidence intervals in parentheses) for variables corrected for financial impact of COVID-19 on family.

Construct	T2			T2 change		
	A	C	E	A	C	E
Love and relationships	.23 (.10-.30)	.00 (.00-.09)	.77 (.70-.84)	.15 (.00-.23)	.00 (.00-.13)	.85 (.77-.92)
Achievement motivation	.38 (.21-.44)	.00 (.00-.14)	.62 (.56-.69)	.15 (.00-.23)	.00 (.00-.14)	.85 (.77-.93)
Importance of Relationships	.35 (.21-.41)	.00 (.00-.00)	.65 (.59-.71)	.09 (.00-.17)	.00 (.00-.10)	.91 (.83-.99)
Purpose in Life	.41 (.21-.51)	.04 (.00-.20)	.55 (.49-.62)	.05 (.00-.22)	.09 (.00-.18)	.87 (.78-.94)
Healthcare	.13 (.00-.28)	.07 (.00-.21)	.80 (.72-.88)	.08 (.00-.20)	.03 (.00-.15)	.89 (.80-.97)
Community satisfaction	.29 (.14-.36)	.00 (.00-.00)	.71 (.64-.78)	.14 (.04-.22)	.00 (.00-.06)	.86 (.78-.94)
Attitudes towards money	.17 (.03-.24)	.00 (.00-.00)	.83 (.76-.90)	.09 (.00-.17)	.00 (.00-.09)	.91 (.83-.99)
Alcohol (frequency x quantity)	.40 (.20-.48)	.01 (.00-.17)	.59 (.52-.66)	.18 (.03-.26)	.00 (.00-.00)	.82 (.74-.91)
Conduct problems	.05 (.00-.27)	.13 (.00-.23)	.81 (.73-.89)	.04 (.00-.23)	.10 (.00-.19)	.86 (.77-.93)
Emotional problems	.38 (.19-.44)	.00 (.00-.15)	.62 (.56-.69)	.22 (.09-.29)	.00 (.00-.09)	.78 (.71-.86)
Hyperactivity	.33 (.24-.40)	.00 (.00-.06)	.67 (.60-.74)	.20 (.00-.28)	.00 (.00-.16)	.80 (.72-.88)
Peer problems	.38 (.19-.49)	.05 (.00-.21)	.57 (.51-.64)	.18 (.00-.26)	.00 (.00-.18)	.82 (.74-.90)
Prosocial behaviour	.34 (.14-.43)	.02 (.00-.18)	.64 (.57-.71)	.03 (.00-.23)	.12 (.00-.20)	.85 (.77-.92)

General anxiety	.42 (.28-.48)	.00 (.00-.11)	.58 (.52-.64)	.25 (.10-.33)	.00 (.00-.11)	.75 (.67-.83)
Depression	.37 (.22-.44)	.00 (.00-.00)	.63 (.56-.69)	.11 (.00-.21)	.02 (.00-.16)	.87 (.79-.95)
Physical activity	.29 (.09-.47)	.13 (.00-.28)	.59 (.52-.66)	.16 (.00-.34)	.10 (.00-.26)	.74 (.66-.83)
Media use	.44 (.35-.50)	.00 (.00-.07)	.56 (.50-.62)	.26 (.16-.33)	.00 (.00-.07)	.74 (.67-.81)
Volunteering	.34 (.26-.41)	.00 (.00-.05)	.66 (.59-.72)	.28 (.19-.36)	.00 (.00-.00)	.72 (.64-.79)

Note: Scores were corrected for mean age and sex differences (see Methods).

Supplementary Table 61. Model fitting results for univariate analyses of additive genetic (A), shared environmental (C), and non-shared environmental (E) components of variance for variables (95% confidence intervals in parentheses) for variables corrected for health and financial impact of COVID-19 on family.

**Construct**	**T2**			**T2 change**		
	**A**	**C**	**E**	**A**	**C**	**E**
Love and relationships	.23 (.10-.30)	.00 (.00-.09)	.77 (.70-.84)	.15 (.00-.23)	.00 (.00-.13)	.85 (.77-.92)
Achievement motivation	.38 (.21-.44)	.00 (.00-.14)	.62 (.56-.69)	.15 (.00-.23)	.00 (.00-.14)	.85 (.77-.93)
Importance of Relationships	.35 (.21-.41)	.00 (.00-.11)	.65 (.59-.71)	.09 (.00-.17)	.00 (.00-.10)	.91 (.83-.99)
Purpose in Life	.42 (.23-.52)	.04 (.00-.19)	.54 (.48-.61)	.05 (.00-.22)	.09 (.00-.18)	.87 (.78-.94)
Healthcare	.13 (.00-.28)	.07 (.00-.22)	.80 (.72-.88)	.08 (.00-.20)	.03 (.00-.15)	.89 (.80-.97)
Community satisfaction	.29 (.14-.36)	.00 (.00-.11)	.71 (.64-.78)	.14 (.04-.22)	.00 (.00-.06)	.86 (.78-.94)

Attitudes towards money	.17 (.03-.24)	.00 (.00-.00)	.83 (.76-.90)	.09 (.00-.17)	.00 (.00-.09)	.91 (.83-.99)
Alcohol (frequency x quantity)	.40 (.20-.48)	.01 (.00-.17)	.59 (.52-.66)	.18 (.03-.26)	.00 (.00-.10)	.82 (.74-.91)
Conduct problems	.05 (.00-.27)	.13 (.00-.23)	.81 (.73-.89)	.04 (.00-.23)	.10 (.00-.19)	.86 (.77-.93)
Emotional problems	.38 (.19-.44)	.00 (.00-.15)	.62 (.56-.69)	.22 (.09-.29)	.00 (.00-.09)	.78 (.71-.86)
Hyperactivity	.34 (.25-.40)	.00 (.00-.06)	.66 (.60-.74)	.20 (.00-.28)	.00 (.00-.16)	.80 (.72-.88)
Peer problems	.38 (.18-.49)	.05 (.00-.21)	.57 (.51-.64)	.18 (.00-.26)	.00 (.00-.18)	.82 (.74-.90)
Prosocial behaviour	.34 (.13-.43)	.02 (.00-.18)	.64 (.57-.71)	.03 (.00-.23)	.12 (.00-.20)	.85 (.77-.92)
General anxiety	.43 (.27-.49)	.00 (.00-.12)	.57 (.51-.64)	.25 (.10-.33)	.00 (.00-.11)	.75 (.67-.83)
Depression	.38 (.23-.44)	.00 (.00-.12)	.62 (.56-.69)	.11 (.00-.21)	.02 (.00-.16)	.87 (.79-.95)
Physical activity	.29 (.10-.47)	.12 (.00-.28)	.58 (.52-.66)	.16 (.00-.34)	.10 (.00-.26)	.74 (.66-.83)
Media use	.44 (.35-.50)	.00 (.00-.07)	.56 (.50-.62)	.26 (.16-.33)	.00 (.00-.07)	.74 (.67-.81)
Volunteering	.35 (.26-.41)	.00 (.00-.06)	.65 (.59-.72)	.28 (.19-.36)	.00 (.00-.00)	.72 (.64-.79)

Note: Scores were corrected for mean age and sex differences (see Methods).

Supplementary Table 62. Model fitting results for univariate analyses of additive genetic (A), shared environmental (C), and non-shared environmental (E) components of variance for variables (95% confidence intervals in parentheses) for variables corrected for worries of being infected.

**Construct**	**T2**			**T2 change**		
	**A**	**C**	**E**	**A**	**C**	**E**

Love and relationships	.23 (.10-.30)	.00 (.00-.09)	.77 (.70-.84)	.15 (.00-.23)	.00 (.00-.00)	.85 (.77-.92)
Achievement motivation	.38 (.22-.44)	.00 (.00-.00)	.62 (.56-.69)	.15 (.00-.23)	.00 (.00-.14)	.85 (.77-.93)
Importance of Relationships	.35 (.21-.41)	.00 (.00-.00)	.65 (.59-.71)	.09 (.00-.17)	.00 (.00-.10)	.91 (.83-.98)
Purpose in Life	.43 (.24-.52)	.03 (.00-.18)	.54 (.48-.60)	.06 (.00-.23)	.09 (.00-.19)	.86 (.77-.93)
Healthcare	.12 (.00-.28)	.08 (.00-.22)	.80 (.72-.88)	.08 (.00-.20)	.04 (.00-.15)	.89 (.80-.97)
Community satisfaction	.29 (.15-.36)	.00 (.00-.11)	.71 (.64-.78)	.14 (.05-.22)	.00 (.00-.06)	.86 (.78-.94)
Attitudes towards money	.17 (.03-.24)	.00 (.00-.10)	.83 (.76-.90)	.09 (.00-.17)	.00 (.00-.00)	.91 (.83-.99)
Alcohol (frequency x quantity)	.39 (.19-.48)	.02 (.00-.18)	.59 (.52-.66)	.18 (.03-.26)	.00 (.00-.00)	.82 (.74-.91)
Conduct problems	.04 (.00-.26)	.14 (.00-.23)	.82 (.73-.89)	.02 (.00-.22)	.12 (.00-.19)	.86 (.78-.93)
Emotional problems	.34 (.13-.41)	.00 (.00-.16)	.66 (.59-.73)	.20 (.04-.27)	.00 (.00-.00)	.80 (.73-.88)
Hyperactivity	.33 (.25-.40)	.00 (.00-.05)	.67 (.60-.74)	.20 (.00-.27)	.00 (NA-.15)	.80 (.73-.88)
Peer problems	.38 (.19-.49)	.04 (.00-.20)	.57 (.51-.64)	.18 (.00-.25)	.00 (.00-.18)	.82 (.75-.91)
Prosocial behaviour	.33 (.13-.42)	.02 (.00-.18)	.65 (.58-.72)	.03 (.00-.23)	.11 (.00-.20)	.85 (.77-.92)
General anxiety	.41 (.30-.47)	.00 (.00-.00)	.59 (.53-.66)	.24 (.08-.32)	.00 (.00-.00)	.76 (.68-.85)
Depression	.37 (.22-.44)	.00 (.00-.12)	.63 (.56-.70)	.08 (.00-.21)	.04 (.00-.16)	.88 (.79-.96)
Physical activity	.27 (.07-.46)	.14 (.00-.29)	.60 (.53-.67)	.15 (.00-.34)	.11 (.00-.26)	.74 (.66-.83)
Media use	.44 (.34-.49)	.00 (.00-.07)	.56 (.51-.62)	.26 (.16-.33)	.00 (.00-.00)	.74 (.67-.81)

Volunteering	.34 (.26-.41)	.00 (.00-.00)	.66 (.59-.73)	.28 (.18-.35)	.00 (.00-.06)	.72 (.65-.80)

Note: Scores were corrected for mean age and sex differences (see Methods).

Supplementary Table 63. Model fitting results for univariate analyses of additive genetic (A), shared environmental (C), and non-shared environmental (E) components of variance for variables (95% confidence intervals in parentheses) for variables corrected for worries of friends or family being infected.

**Construct**	**T2**			**T2 change**		
	**A**	**C**	**E**	**A**	**C**	**E**
Love and relationships	.23 (.10-.30)	.00 (.00-.09)	.77 (.70-.84)	.15 (.00-.23)	.00 (.00-.00)	.85 (.77-.92)
Achievement motivation	.38 (.22-.44)	.00 (.00-.13)	.62 (.56-.68)	.15 (.00-.23)	.00 (.00-.14)	.85 (.77-.93)
Importance of Relationships	.35 (.20-.41)	.00 (.00-.00)	.65 (.59-.72)	.09 (.00-.17)	.00 (.00-.09)	.91 (.83-.99)
Purpose in Life	.43 (.24-.52)	.04 (.00-.19)	.54 (.48-.60)	.04 (.00-.23)	.10 (.00-.19)	.86 (.77-.93)
Healthcare	.11 (.00-.27)	.08 (.00-.21)	.81 (.73-.89)	.07 (.00-.20)	.04 (.00-.15)	.89 (.80-.97)
Community satisfaction	.29 (.15-.36)	.00 (.00-.11)	.71 (.64-.78)	.14 (.05-.22)	.00 (.00-.06)	.86 (.78-.94)
Attitudes towards money	.17 (.03-.24)	.00 (.00-.10)	.83 (.76-.90)	.09 (.00-.17)	.00 (.00-.00)	.91 (.83-.99)
Alcohol (frequency x quantity)	.41 (.20-.48)	.01 (.00-.17)	.58 (.52-.66)	.18 (.03-.26)	.00 (.00-.10)	.82 (.74-.91)
Conduct problems	.05 (.00-.27)	.13 (.00-.23)	.81 (.73-.89)	.03 (.00-.23)	.11 (.00-.19)	.86 (.77-.93)

Emotional problems	.29 (.08-.40)	.04 (.00-.20)	.67 (.60-.74)	.20 (.03-.28)	.00 (.00-.00)	.80 (.72-.88)
Hyperactivity	.33 (.24-.39)	.00 (.00-.06)	.67 (.61-.74)	.20 (.00-.27)	.00 (.00-.16)	.80 (.73-.89)
Peer problems	.38 (.18-.49)	.05 (.00-.21)	.57 (.51-.64)	.18 (.00-.26)	.00 (.00-.18)	.82 (.74-.90)
Prosocial behaviour	.34 (.14-.40)	.00 (.00-.00)	.66 (.60-.74)	.05 (.00-.23)	.09 (.00-.19)	.86 (.77-.93)
General anxiety	.38 (.22-.44)	.00 (.00-.00)	.62 (.56-.69)	.24 (.04-.31)	.00 (.00-.00)	.76 (.69-.85)
Depression	.36 (.17-.43)	.00 (.00-.14)	.64 (.57-.71)	.05 (.00-.20)	.07 (.00-.16)	.89 (.80-.96)
Physical activity	.29 (.09-.47)	.12 (.00-.28)	.59 (.52-.66)	.17 (.00-.34)	.09 (.00-.26)	.74 (.66-.83)
Media use	.44 (.35-.50)	.00 (.00-.07)	.56 (.50-.62)	.27 (.16-.33)	.00 (.00-.08)	.73 (.67-.81)
Volunteering	.34 (.26-.41)	.00 (.00-.05)	.66 (.59-.73)	.28 (.19-.36)	.00 (.00-.06)	.72 (.64-.80)

Note: Scores were corrected for mean age and sex differences (see Methods).

Supplementary Table 64. Model fitting results for univariate analyses of additive genetic (A), shared environmental (C), and non-shared environmental (E) components of variance for variables (95% confidence intervals in parentheses) for variables corrected for worries of mental/emotional health being influenced by COVID-19.

**Construct**	**T2**			**T2 change**		
	**A**	**C**	**E**	**A**	**C**	**E**
Love and relationships	.22 (.09-.29)	.00 (.00-.00)	.78 (.71-.86)	.15 (.00-.22)	.00 (.00-.14)	.85 (.78-.93)
Achievement motivation	.38 (.21-.44)	.00 (.00-.13)	.62 (.56-.69)	.15 (.00-.23)	.00 (.00-.14)	.85 (.77-.93)

Importance of Relationships	.35 (.19-.41)	.00 (.00-.13)	.65 (.59-.72)	.09 (.00-.17)	.00 (.00-.10)	.91 (.83-.98)
Purpose in Life	.42 (.23-.52)	.03 (.00-.19)	.54 (.48-.61)	.07 (.00-.24)	.09 (.00-.20)	.84 (.76-.92)
Healthcare	.14 (.00-.28)	.06 (.00-.21)	.80 (.72-.88)	.09 (.00-.20)	.03 (.00-.15)	.88 (.80-.97)
Community satisfaction	.29 (.15-.36)	.00 (.00-.11)	.71 (.64-.78)	.14 (.05-.22)	.00 (.00-.00)	.86 (.78-.94)
Attitudes towards money	.17 (.03-.24)	.00 (.00-.10)	.83 (.76-.91)	.09 (.00-.17)	.00 (.00-.00)	.91 (.83-.99)
Alcohol (frequency x quantity)	.41 (.20-.48)	.01 (.00-.17)	.58 (.52-.66)	.18 (.03-.26)	.00 (.00-.10)	.82 (.74-.91)
Conduct problems	.06 (.00-.27)	.13 (.00-.23)	.81 (.73-.89)	.04 (.00-.23)	.10 (.00-.19)	.85 (.77-.93)
Emotional problems	.26 (.05-.36)	.03 (.00-.20)	.71 (.64-.79)	.19 (.02-.27)	.00 (.00-.14)	.81 (.73-.88)
Hyperactivity	.3 (.22-.37)	.00 (.00-.05)	.7 (.63-.77)	.20 (.05-.27)	.00 (.00-.11)	.80 (.73-.88)
Peer problems	.39 (.19-.48)	.03 (.00-.19)	.58 (.52-.65)	.18 (.00-.26)	.00 (.00-.18)	.82 (.74-.90)
Prosocial behaviour	.34 (.13-.42)	.02 (.00-.18)	.64 (.58-.72)	.03 (.00-.23)	.12 (.00-.20)	.86 (.77-.92)
General anxiety	.31 (.16-.37)	.00 (.00-.11)	.69 (.63-.77)	.20 (.00-.27)	.00 (.00-.15)	.80 (.73-.89)
Depression	.3 (.13-.37)	.00 (.00-.00)	.7 (.63-.77)	.08 (.00-.19)	.03 (.00-.14)	.9 (.81-.98)
Physical activity	.27 (.08-.46)	.13 (.00-.29)	.59 (.53-.66)	.15 (.00-.34)	.10 (.00-.26)	.74 (.66-.83)
Media use	.42 (.33-.48)	.00 (.00-.00)	.58 (.52-.64)	.26 (.15-.32)	.00 (.00-.08)	.74 (.68-.82)
Volunteering	.34 (.26-.41)	.00 (.00-.05)	.66 (.59-.73)	.28 (.19-.36)	.00 (.00-.06)	.72 (.64-.80)

Note: Scores were corrected for mean age and sex differences (see Methods).

Supplementary Table 65. Model fitting results for univariate analyses of additive genetic (A), shared environmental (C), and non-shared environmental (E) components of variance for variables (95% confidence intervals in parentheses) for variables corrected for worries of physical health being influenced by COVID-19.

Construct	T2			T2 change		
	A	C	E	A	C	E
Love and relationships	.23 (.10-.30)	.00 (.00-.09)	.77 (.70-.85)	.15 (.00-.23)	.00 (.00-.13)	.85 (.77-.92)
Achievement motivation	.38 (.20-.44)	.00 (.00-.14)	.62 (.56-.69)	.15 (.00-.23)	.00 (.00-.15)	.85 (.77-.93)
Importance of Relationships	.36 (.22-.42)	.00 (.00-.11)	.64 (.58-.71)	.09 (.00-.17)	.00 (.00-.10)	.91 (.83-.98)
Purpose in Life	.43 (.23-.52)	.04 (.00-.19)	.54 (.48-.61)	.06 (.00-.23)	.08 (.00-.19)	.86 (.77-.93)
Healthcare	.13 (.00-.28)	.07 (.00-.21)	.80 (.72-.88)	.08 (.00-.20)	.03 (.00-.15)	.89 (.80-.97)
Community satisfaction	.29 (.15-.36)	.00 (.00-.11)	.71 (.64-.78)	.15 (.05-.22)	.00 (.00-.06)	.85 (.78-.93)
Attitudes towards money	.17 (.03-.24)	.00 (.00-.00)	.83 (.76-.91)	.09 (.00-.17)	.00 (.00-.09)	.91 (.83-.99)
Alcohol (frequency x quantity)	.41 (.20-.48)	.01 (.00-.17)	.58 (.52-.66)	.18 (.03-.26)	.00 (.00-.00)	.82 (.74-.91)
Conduct problems	.04 (.00-.26)	.15 (.00-.23)	.82 (.73-.89)	.02 (.00-.22)	.12 (.00-.19)	.86 (.77-.93)
Emotional problems	.33 (.14-.40)	.00 (.00-.15)	.67 (.60-.74)	.20 (.06-.28)	.00 (.00-.10)	.80 (.72-.88)
Hyperactivity	.32 (.23-.39)	.00 (.00-.06)	.68 (.61-.75)	.20 (.00-.27)	.00 (.00-.00)	.80 (.73-.89)

Peer problems	.36 (.16-.48)	.06 (.00-.22)	.58 (.52-.65)	.17 (.00-.25)	.01 (.00-.18)	.82 (.75-.91)
Prosocial behaviour	.34 (.14-.42)	.01 (.00-.17)	.64 (.58-.72)	.04 (.00-.23)	.11 (.00-.20)	.85 (.77-.92)
General anxiety	.39 (.27-.46)	.00 (.00-.00)	.61 (.54-.67)	.23 (.06-.31)	.00 (.00-.12)	.77 (.69-.85)
Depression	.36 (.19-.42)	.00 (.00-.13)	.64 (.58-.71)	.08 (.00-.21)	.04 (.00-.16)	.88 (.79-.95)
Physical activity	.27 (.08-.46)	.13 (.00-.29)	.60 (.53-.67)	.15 (.00-.33)	.11 (.00-.26)	.75 (.66-.83)
Media use	.43 (.34-.49)	.00 (.00-.07)	.57 (.51-.63)	.26 (.16-.33)	.00 (.00-.08)	.74 (.67-.81)
Volunteering	.34 (.26-.41)	.00 (.00-.05)	.66 (.59-.73)	.28 (.18-.35)	.00 (.00-.06)	.72 (.65-.80)

Note: Scores were corrected for mean age and sex differences (see Methods).

Supplementary Table 66. Model fitting results for univariate analyses of additive genetic (A), shared environmental (C), and non-shared environmental (E) components of variance for variables (95% confidence intervals in parentheses) for variables corrected for change in sleep habits.

Construct	T2			T2 change		
	A	C	E	A	C	E
Love and relationships	.23 (.10-.30)	.00 (.00-.09)	.77 (.70-.85)	.15 (.00-.22)	.00 (.00-.00)	.85 (.78-.93)
Achievement motivation	.38 (.21-.44)	.00 (.00-.13)	.62 (.56-.69)	.15 (.00-.23)	.00 (.00-.14)	.85 (.77-.93)
Importance of Relationships	.35 (.21-.41)	.00 (.00-.11)	.65 (.59-.71)	.09 (.00-.17)	.00 (.00-.10)	.91 (.83-.99)
Purpose in Life	.43 (.24-.52)	.03 (.00-.19)	.54 (.48-.61)	.05 (.00-.23)	.09 (.00-.19)	.86 (.77-.93)

Healthcare	.13 (.00-.28)	.07 (.00-.21)	.80 (.72-.88)	.08 (.00-.20)	.03 (.00-.15)	.89 (.80-.97)
Community satisfaction	.29 (.15-.36)	.00 (.00-.11)	.71 (.64-.78)	.14 (.05-.22)	.00 (.00-.06)	.86 (.78-.94)
Attitudes towards money	.17 (.03-.24)	.00 (.00-.10)	.83 (.76-.90)	.09 (.00-.17)	.00 (.00-.09)	.91 (.83-.99)
Alcohol (frequency x quantity)	.41 (.21-.48)	.00 (.00-.00)	.59 (.52-.66)	.17 (.04-.26)	.00 (NA-.10)	.83 (.74-.91)
Conduct problems	.06 (.00-.27)	.13 (.00-.23)	.81 (.73-.89)	.05 (.00-.23)	.10 (.00-.19)	.86 (.77-.93)
Emotional problems	.38 (.18-.44)	.00 (.00-.16)	.62 (.56-.69)	.21 (.08-.29)	.00 (.00-.00)	.79 (.71-.87)
Hyperactivity	.34 (.25-.41)	.00 (.00-.05)	.66 (.59-.73)	.20 (.00-.28)	.00 (.00-.00)	.80 (.72-.88)
Peer problems	.37 (.17-.48)	.05 (.00-.21)	.58 (.52-.65)	.18 (.00-.25)	.00 (.00-.18)	.82 (.75-.91)
Prosocial behaviour	.35 (.14-.43)	.02 (.00-.18)	.64 (.57-.71)	.03 (.00-.23)	.12 (.00-.20)	.85 (.76-.92)
General anxiety	.44 (.29-.50)	.00 (.00-.12)	.56 (.50-.63)	.26 (.08-.34)	.00 (.00-.00)	.74 (.66-.82)
Depression	.39 (.23-.45)	.00 (.00-.12)	.61 (.55-.68)	.09 (.00-.21)	.04 (.00-.16)	.87 (.79-.95)
Physical activity	.29 (.10-.47)	.12 (.00-.28)	.59 (.52-.66)	.17 (.00-.35)	.09 (.00-.26)	.74 (.65-.82)
Media use	.44 (.35-.50)	.00 (.00-.07)	.56 (.50-.62)	.27 (.16-.33)	.00 (NA-NA)	.73 (.67-.81)
Volunteering	.34 (.26-.41)	.00 (.00-.05)	.66 (.59-.73)	.28 (.18-.35)	.00 (.00-.06)	.72 (.65-.80)

Note: Scores were corrected for mean age and sex differences (see Methods).

Supplementary Figures

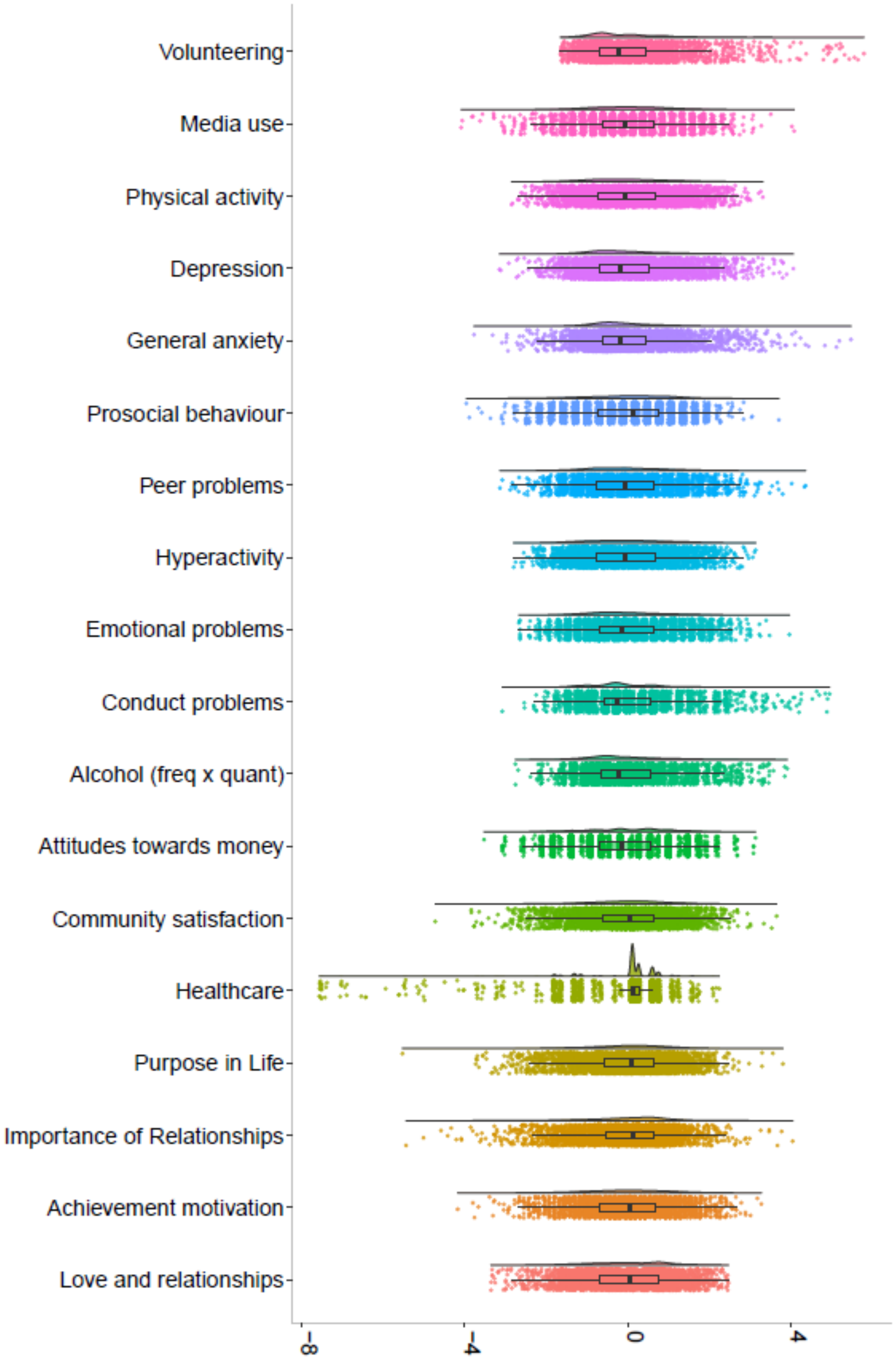

Supplementary Figure 1. Variance in T2 change scores.

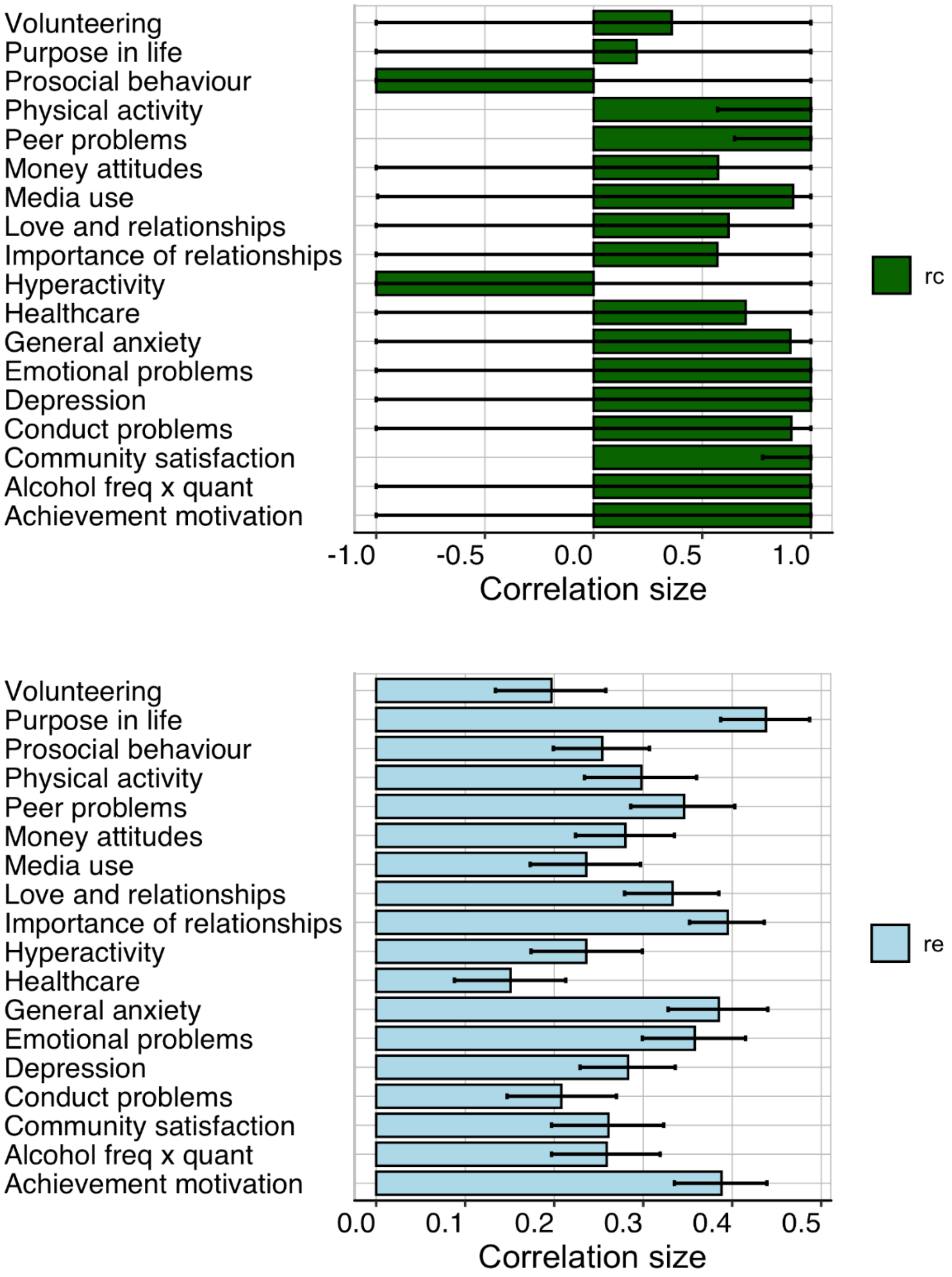

Supplementary Figure 2. Shared environmental (rC) and nonshared environmental correlations (rE).

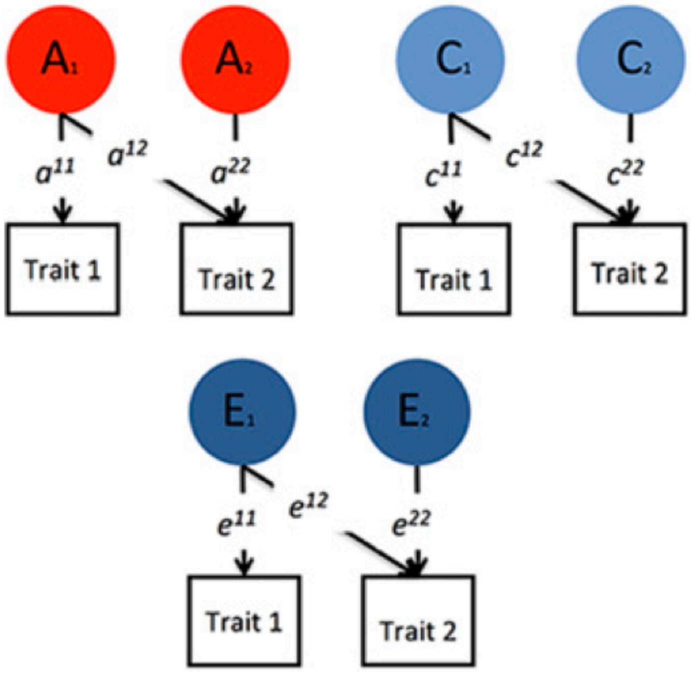

Supplementary Figure 3. The Bivariate Cholesky decomposition.
